# Identification of an immune-related risk signature for predicting prognosis in clear cell renal cell carcinoma

**DOI:** 10.18632/aging.102746

**Published:** 2020-02-06

**Authors:** Xiaoliang Hua, Juan Chen, Yang Su, Chaozhao Liang

**Affiliations:** 1 Department of Urology, The First Affiliated Hospital of Anhui Medical University, Hefei, China; 2 Anhui Province Key Laboratory of Genitourinary Diseases, Anhui Medical University, Hefei, China; 3 The Institute of Urology, Anhui Medical University, Hefei, China; 4 The Key Laboratory of Aquatic Biodiversity and Conservation of Chinese Academy of Sciences, Institute of Hydrobiology, Chinese Academy of Sciences, Wuhan, China; 5 University of Chinese Academy of Sciences, Beijing, China

**Keywords:** clear cell renal cell carcinoma, gene signature, prognostic model, immune-related gene, tumor immunology

## Abstract

Immune status affects the initiation and progression of clear cell renal cell carcinoma (ccRCC), the most common subtype of renal cell carcinoma. In this study, we identified an immune-related, five-gene signature that improves survival prediction in ccRCC. Patients were classified as high- and low-risk based on the signature risk score. Survival analysis showed differential prognosis, while principal component analysis revealed distinctly different immune phenotypes between the two risk groups. High-risk patients tended to have advanced stage, higher grade disease, and poorer prognoses. Functional enrichment analysis showed that the signature genes were mainly involved in the cytokine-cytokine receptor interaction pathway. Moreover, we found that tumors from high-risk patients had higher relative abundance of T follicular helper cells, regulatory T cells, and M0 macrophages, and higher expression of PD-1, CTLA-4, LAG3, and CD47 than low-risk patients. This suggests our gene signature may not only serve as an indicator of tumor immune status, but may be a promising tool to select high-risk patients who may benefit from immune checkpoint inhibitor therapy. Multivariate Cox regression analysis showed that the signature remained an independent prognostic factor after adjusting for clinicopathological variables, while prognostic accuracy was further improved after integrating clinical parameters into the analysis.

## INTRODUCTION

Clear cell renal cell carcinoma (ccRCC) is the most common subtype of renal cell carcinoma (RCC), accounting for approximately 80% to 90% of cases [1]. CcRCC has also more malignant characteristics and worse prognosis, and is responsible for most RCC-related deaths [2]. According to the latest global cancer statistics released in 2018, each year approximately 403,000 people are diagnosed with RCC and 175,000 patients die from the disease [3]. Patients with RCC typically undergo surgical treatment, and improved surgical methods have contributed to favorable overall prognosis [4]. However, uncontrolled tumor progression and death will still occur in approximately 30% of RCC patients despite initial curative surgery [2]. Thus, more effective systemic treatments for RCC patients are needed to improve outcomes.

Targeted therapy agents, including VEGF receptor and mTOR inhibitors, can significantly improve survival in RCC patients with metastasis [5, 6]. However, many RCC patients do not have targetable mutations. The emergence of immune checkpoint targets, such as programmed death-1 (PD-1) and programmed death-ligand-1 (PD-L1) provides another therapeutic strategy for metastatic RCC [7], perhaps more compelling than targeted therapy agents [8], which emphasizes the importance of the tumor immune status on the outcome of RCC patients. Indeed, various components of the tumor immune microenvironment have been shown to be essential during cancer initiation and progression [9]. Chevrier et al. [10] revealed that immune cell composition in the tumor microenvironment was associated with ccRCC patients’ survival. Ghatalia et al. [11] showed that T cell infiltration was associated with the recurrence of ccRCC following surgery. However, actionable immune-related biomarkers for ccRCC patients remain to be comprehensively explored regarding their prognostic potential.

The current classification system for RCC patients to estimate prognosis is mainly based on pathological stages [12]. However, this approach may be insufficient to distinguish patients with high risk of tumor progression. A number of studies have proposed gene expression signature assays to estimate survival in ccRCC patients [13–15]. However, only a few have systematically investigated the immune-related phenotype in ccRCC and its relationship with prognosis. Therefore, a more accurate classification system based on a comprehensive list of immune-related genes (IRGs) is deeply needed to improve prognosis accuracy and direct clinical practice.

In this study we used multiple gene expression datasets to develop and validate a five-IRG signature for ccRCC. We evaluated the correlation between the IRG signature and clinical characteristics and discussed possible mechanisms through which the risk signature may impact ccRCC development. Finally, we integrated the IRG signature with clinical factors to build a prognostic nomogram, which allowed improved prognosis assessment of ccRCC patients.

## RESULTS

### Identification of differently expressed IRGs in ccRCC

**Figure 1:**
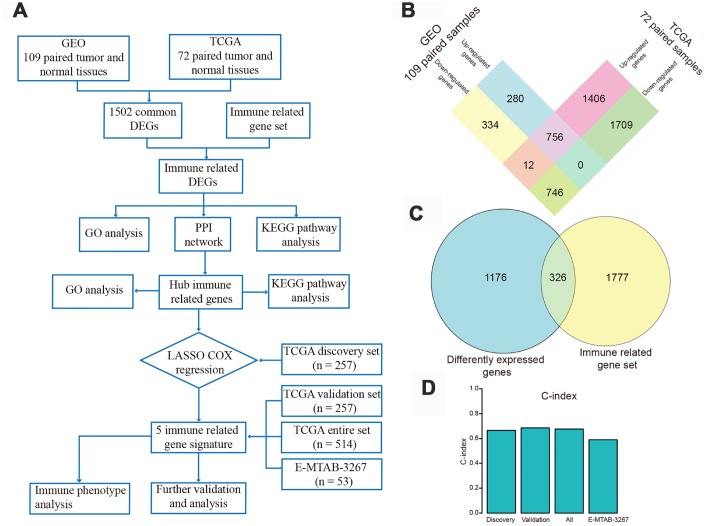
**Flow chart of the study design and identification of differentially expressed genes (DEGs).** (**A**) Flow chart of our research project. (**B**) Using gene expression data of 109 pairs of normal and tumor tissues from Gene Expression Omnibus and 72 paired samples from The Cancer Genome Atlas (TCGA) database, 756 up-regulated and 746 down-regulated DEGs were identified and extracted from the two databases. (**C**) These DEGs were intersected with a gene set including 2103 immune-related genes (IRGs), and 326 differently expressed IRGs were identified. (**D**) Harrell's concordance index of the five-gene signature in the TCGA discovery, validation, and entire sets, and in the E-MTAB-3267 dataset.

We analyzed 109 paired tumor and adjacent normal tissue samples from ccRCC patients available in the Gene Expression Omnibus (GEO) database and identified 2,128 differently expressed genes (DEGs), of which 1,036 were up-regulated and 1,092 were down-regulated. From 72 paired specimens in The Cancer Genome Atlas (TCGA), we further identified 4,629 DEGs, including 2,174 up-regulated and 2,455 down-regulated transcripts. Overlapping DEGs between GEO and TCGA datasets (756 up-regulated and 746 down-regulated transcripts) were next extracted (Figure 1B) and intersected with an IRG set composed of lists combined from ImmPort and InnateDB databases. A total of 326 differentially expressed IRGs was thus obtained (Figure 1C and Supplementary Table 1). Principal component analysis (PCA) of these IRGs showed varying distribution patterns, indicating distinct immune phenotypes in normal tissue and tumor samples both in GEO (Supplementary Figure 1A) and TCGA datasets (Supplementary Figure 1B). As expected, Gene Ontology (GO) analysis results revealed that inflammatory and immune pathways were most significantly enriched with these IRGs. On GO analysis, “inflammatory response”, “extracellular space”, and “receptor activity” were the most significant terms among biological processes, cellular components, and molecular functions, respectively (Supplementary Table 2). The top 20 GO terms are presented in Supplementary Figure 2A. Kyoto Encyclopedia of Genes and Genomes (KEGG) analysis revealed that “cytokine-cytokine receptor interaction” was the pathway most significantly enriched with the differentially expressed IRGs (Supplementary Figure 2B). Protein Analysis Through Evolutionary Relationships (PANTHER) pathway analysis revealed that immune-related pathways, such as interleukin signaling pathway, inflammation mediated by chemokine and cytokine signaling pathway, B cell activation, and T cell activation, were associated with these differentially expressed IRGs (Supplementary Table 3). Additional results from disease ontology (DO) analysis confirmed that this IRG set was associated with immune status and showed also involvement in kidney disease (Supplementary Table 4). The top 15 most significantly enriched diseases are presented in Supplementary Figure 2C.

### Identification of hub IRGs

**Figure 2:**
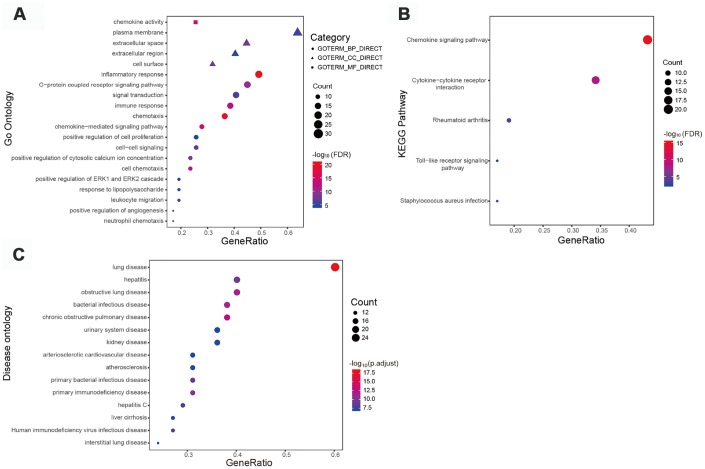
**Functional enrichment of 47 immune-related hub genes.** (**A**) Top 20 most significant Gene Ontology (GO) terms identified by GO analysis. (**B**) Kyoto Encyclopedia of Genes and Genomes pathway analysis. FDR < 0.01 indicated significant enrichment. (**C**) Top 15 most significant disease ontology (DO) terms identified by DO analysis.

We next imported the identified IRGs into the STRING database to create a protein-protein interaction (PPI) network, thus obtaining 47 hub genes defined by a connectivity degree > 20 and an interaction score > 0.7. In line with the above GO analysis results, GO analysis of these hub genes revealed that “inflammatory response”, “extracellular space”, and “chemokine activity” were the terms most significantly enriched (Supplementary Table 5). Figure 2A shows the top 20 GO terms related to the hub IRGs. On KEGG pathway analysis “chemokine signaling pathway” was the most frequently enriched process (Figure 2B), and highly concordant results were obtained for diverse immune-related pathways after data analysis using the PANTHER algorithm (Supplementary Table 6). Meanwhile, DO analysis results (Supplementary Table 7) confirmed the association of these hub IRGs with kidney disease (Figure 2C).

### Identification of a five-IRG signature in the TCGA discovery set

We used the least absolute shrinkage and selection operator (LASSO) Cox regression model to identify the most valuable prognostic IRGs based on their association with patient survival in our TCGA discovery set (Supplementary Figure 3). Five IRGs, namely SHC1, IRF7, KDR, JAK3, and CXCL5 were thus selected (see detailed information in Supplementary Table 8). Univariate Cox analysis results for these IRGs are shown in Table 1. All five IRGs were up-regulated and correlated negatively with patient survival (P < 0.05) except for KDR, which showed a positive relationship. The same results were obtained on Kaplan-Meier (K-M) analyses (Supplementary Figure 4A). The prognostic value of these 5 genes was further validated in the Gene Expression Profiling Interactive Analysis (GEPIA) database. Here, consistent results were obtained, except for SHC1 (Supplementary Figure 4B). The relative expression of each gene in our IRG signature was further verified by qRT-PCR in 35 paired ccRCC/normal specimens collected at our institution. Results corroborated significantly higher expression in tumor samples (Supplementary Figure 5), in agreement with the database validation approach.

**Table 1:** Univariate Cox analysis of the association of the five immune-related genes (IRGs) with overall survival in the TCGA discovery set (n = 257).

**Gene name**	**HR**	**95% CI**	***P* value**
SHC1	1.9220	1.317-2.806	<0.001
IRF7	1.4807	1.178-1.861	<0.001
KDR	0.7343	0.633-0.852	<0.001
JAK3	1.4136	1.152-1.735	<0.001
CXCL5	1.1115	1.040-1.189	0.002

Abbreviations: HR, hazard ratio; CI, confidence interval; IRGs, immune-related genes.

Based on the respective expression levels, we then established the following risk score formula:

Risk score = (0.08995984 * SHC1) + (0.05754872 * IRF7) + (-0.13910054 * KDR) + (0.01889022 * JAK3) + (0.02791388 * CXCL5)

**Figure 3:**
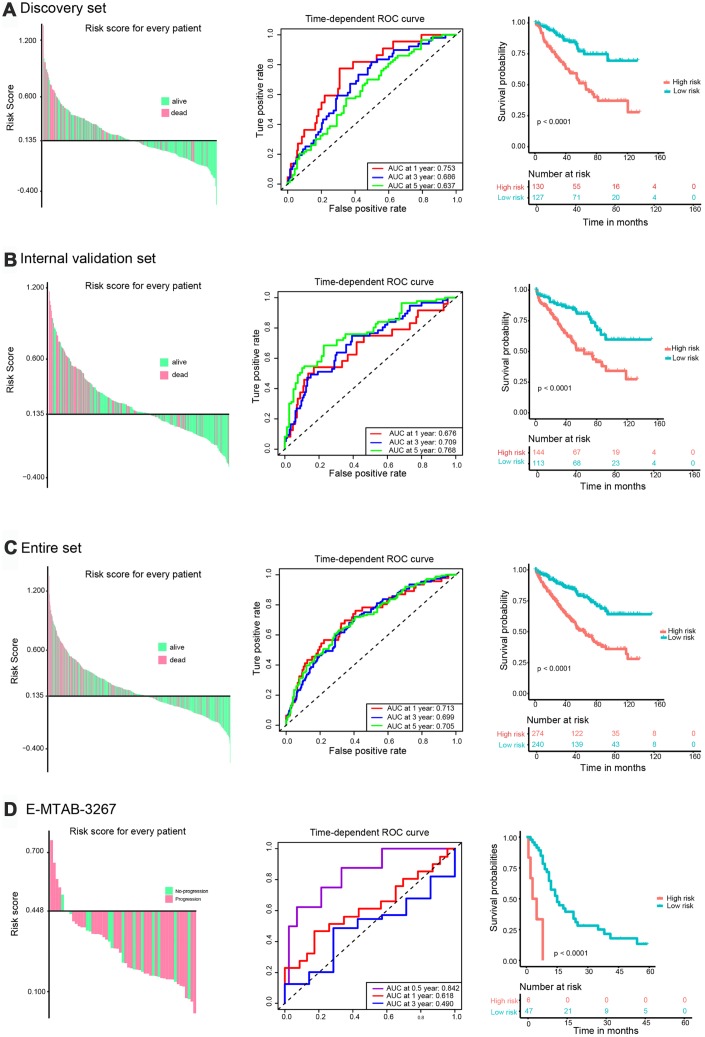
**Validation of the prognostic risk signature.** Left panel: Distribution of the risk signature based on survival status. High-risk and low-risk patients were distributed above and below the x-axis, respectively. Pink and green colors indicate dead and alive patients, respectively. Middle panel: Time-dependent ROC curves were performed to evaluate the accuracy of the risk signature. Right panel: Kaplan-Meier survival curves were performed to assess patients’ prognosis. (**A**) TCGA discovery set. (**B**) Validation set. (**C**) Entire set. (**D**) E-MTAB-3267.

The C-index of the risk score in the TCGA discovery set was 0.666 (95% confidence interval [CI], 0.603-0.729; Figure 1D). Using X-tile software, the optimum cut-off value to distinguish high-risk (n = 130) from low-risk (n = 127) patients was 0.135 (Supplementary Figure 6A). High-risk patients showed poorer prognosis, while low-risk patients had better overall survival (Figure 3A, left panel). Time-dependent receiver operating characteristic (ROC) analysis was performed to evaluate the accuracy of the five-gene signature in predicting patient survival. The area under the curve (AUC) was 0.753 at 1 year, 0.686 at 3 years, and 0.637 at 5 years (Figure 3A, middle panel). K-M analysis corroborated that high-risk patients had a significantly lower survival rate than low-risk cases (*P* < 0.001, Figure 3A, right panel).

### Validation of the five-IRG signature

The prognostic value of the five-IRG signature was further evaluated in three validation sets (TCGA internal validation set, TCGA entire set, and the E-MTAB-3267 dataset). We calculated the risk score for each patient using the same formula. Patients in TCGA internal and entire sets were classified into high-risk and low-risk groups with the cut-off value (0.135) used in TCGA discovery set. Patients in the E-MTAB-3267 dataset were divided into two groups with a cut-off value of 0.448 (Supplementary Figure 6B) determined using X-tile software. The C-indexes of risk scores in TCGA internal, TCGA entire, and E-MTAB-3267 sets were 0.686 (95% CI, 0.623-0.749), 0.677 (95% CI, 0.634-0.720), and 0.591 (95% CI, 0.481-0.701), respectively (Figure 1D). Consistent with the results from the TCGA discovery set, high-risk patients in the three validation sets had poorer prognosis than those in the low-risk group (Figure 3B–3D, left panel). The ROC curves of the five-gene signature in the three validation sets showed good performance (Figures 3B–3D, middle panel). Survival analysis in the three validation sets confirmed lower survival rate in the high-risk groups (Figures 3B–3D, right panel).

### Association with clinicopathological factors and sub-group analysis

Results of the correlation analysis between clinical factors and our IRG signature’s risk score is shown in Table 2. The risk score of the signature was correlated with gender (*P* < 0.001), stage (*P* < 0.001), and grade (*P* < 0.001), but not with age (*P* = 0.347). Indeed, we found that male gender, advanced stage, high grade, and high-risk patients tended to have higher risk score (*P* < 0.05, Supplementary Figure 7). Subgroup analysis was performed to further assess whether the five-IRG signature had prognostic value for survival within specific clinical parameters. The results showed that the our risk signature was still a powerful tool for predicting survival in younger (age < 65) and older (age ≥ 65) patients, male or female patients, low (Fuhrman grade 1 & 2) or high (Fuhrman grade 3 & 4) grade patients, and advanced disease stage (stage III & IV) patients (*P* < 0.05, Supplementary Figure 8). In the subgroup of early stage (stage I & II), the prognosis of the high-risk group also tended be poorer than the low-risk group, although this difference did not reach significance (*P* = 0.056). Overall, the risk score based on our IRG signature was able to predict overall survival independently of specific clinical factors in ccRCC patients.

**Table 2:** Association between risk score of the five-IRG signature and patients' clinical characteristics.

**Variables**	**Entire TCGA set n (%)**	**Risk score**		***P* value**
		**Low**	**High**	
Age (mean ± SD, years)	60.5 ± 12.2	59.9 ± 12.7	61.0 ± 11.7	0.347
Gender				4.777e-05
Male	337 (65.6)	135 (40.1)	202 (59.9)	
Female	177 (34.4)	105 (59.3)	72 (40.7)	
Stage				1.008e-09
I	258 (50.2)	155 (60.1)	103 (39.9)	
II	54 (10.5)	21 (38.9)	33 (61.1)	
III	120 (23.3)	47 (39.2)	73 (60.8)	
IV	82 (16.0)	17 (20.7)	65 (79.3)	
Grade				1.066e-10
G1	13 (2.5)	11 (84.6)	2 (15.4)	
G2	224 (43.6)	133 (59.4)	91 (40.6)	
G3	204 (39.7)	83 (40.7)	121 (59.3)	
G4	73 (14.2)	13 (17.8)	60 (82.2)	

### Distinct immune phenotypes characterize high-risk and low-risk ccRCC patients

**Figure 4:**
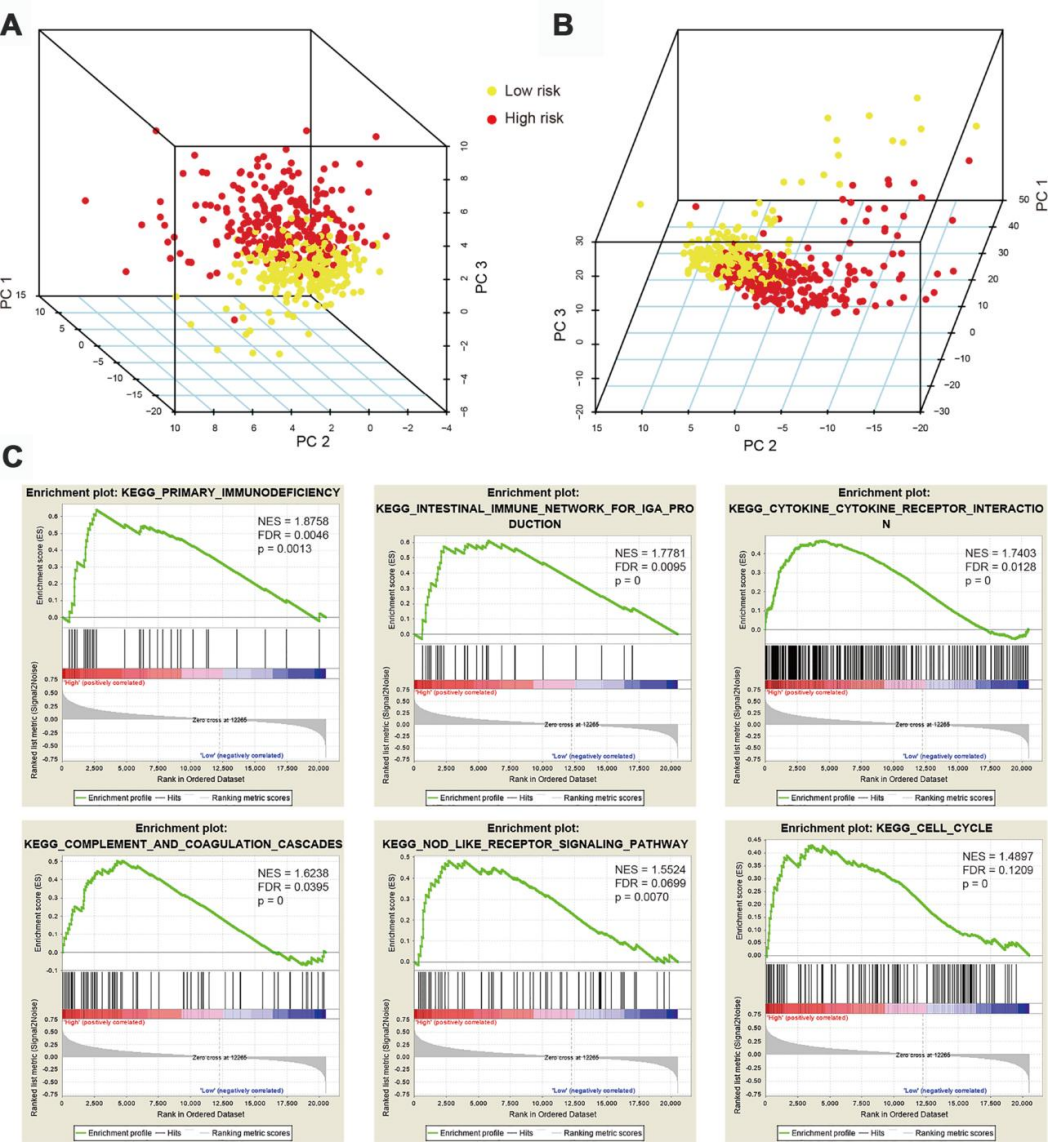
**Principal component analysis (PCA) and gene set enrichment analysis (GSEA).** (**A**) PCA based on 47 immune-related hub genes (IRGs) showing distinct immune phenotypes in high- and low-risk patient groups. (**B**) Distribution patterns for the two risk groups based on 326 differentially expressed IRGs. (**C**) GSEA results showing significant enrichment of immune-related phenotype in high-risk patients.

Considering the distinct prognosis in high-risk and low-risk ccRCC patients, we sought to explore whether phenotypic differences in immune cell populations are found upon segregation of cases based on our IRG signature’s specific risk score. PCA based on the 47 hub IRGs (Figure 4A) and the 326 differentially expressed IRGs (Figure 4B) showed different distribution patterns, indicating remarkably different immune phenotypes for high- and low-risk groups. Gene Set Enrichment Analysis (GSEA) was also performed to explore whether immunological pathways differ between risk groups. As expected, significant alterations in immunological pathways were detected in high-risk patients, compared with low-risk ones (Figure 4C). Enriched pathways and processes in tumors from high-risk ccRCC patients included primary immunodeficiency, intestinal immune network for IgA production, cytokine-cytokine receptor interaction, complement and coagulation cascades, Nod-like receptor signaling pathway, and cell cycle.

### Immune landscapes differ between low-risk and high-risk ccRCC patients

**Figure 5:**
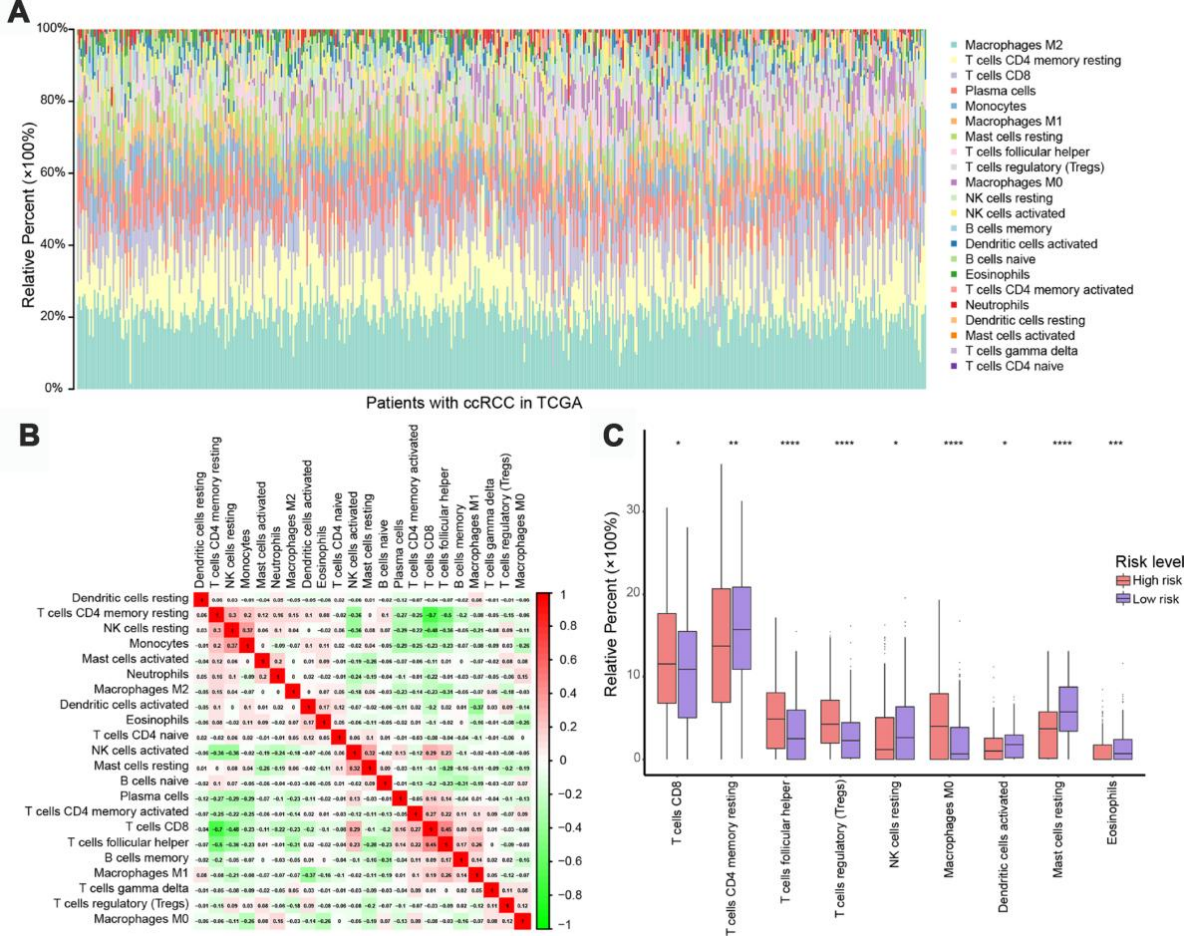
**Immune landscapes in high-risk and low-risk patients.** (**A**) Relative proportions of 22 immune cell types in high- and low-risk patients. (**B**) Correlation matrix of relative proportions of the 22 immune cell types. (**C**) Box plots showing differential immune cell infiltration status between high- and low-risk patients. *P < 0.05, **P < 0.01, ***P < 0.001, ****P < 0.0001.

To assess whether the five-IRG signature could accurately reflect the status of the tumor immune microenvironment, we estimated infiltration rates for 22 immune cell types in ccRCC patients through CIBERSOFT algorithm, and investigated potential differences between the low- and high-risk groups. As shown in Figure 5A, the distribution of immune cell types varied within and between groups, suggesting that the immune landscape in ccRCC cases might be an inherent feature of each individual.

The relative proportions of the 22 immune cell types were weakly to moderately correlated with risk classification (Figure 5B). Patients in the high-risk group had significantly higher representation of CD8 T^+^ cells, T follicular helper cells, regulatory T cells (Tregs), and M0 macrophages, and significantly lower abundance of natural killer (NK) cells, plasma cells, dendritic cells, mast cells, and eosinophils (*P* < 0.05, Figure 5C), indicating a weakened immune phenotype in high-risk patients. These data suggest that the five-IRG signature could serve as an indicator of immune status in ccRCC.

**Figure 6:**
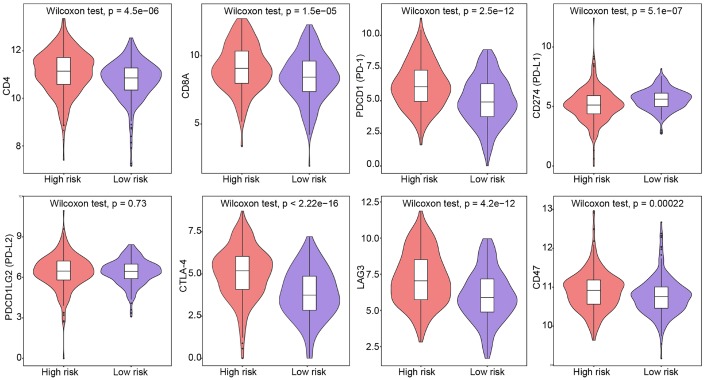
**Expression of T-cell and immune checkpoint markers in low-risk and high-risk patients.** Group differences were assessed by Wilcoxon test.

We also evaluated the correlation between the IRG signature’s risk score and the expression of T-cell markers, including CD4 and CD8A, and immune checkpoint genes such as PD-1 and its ligands (PD-L1, PD-L2), cytotoxic T-lymphocyte associated protein 4 (CTLA-4), lymphocyte-activation gene 3 (LAG3), and CD47. We found that the risk score was positively correlated with the expression of CD4, CD8A, PD-1, CTLA-4, LAG3, and CD47, and negatively correlated with PD-L1 expression (*P* < 0.05, Supplementary Figure 9). Next, we contrasted these findings in low- and high-risk ccRCC patients. In strong agreement with the risk classification derived from our five-IRG signature, we found that CD4, CD8A, PD-1, CTLA-4, LAG3, and CD47 were significantly overexpressed in high-risk patients, compared to low-risk ones (*P* < 0.05; Figure 6). These data further suggest the presence of an immunosuppressive tumor microenvironment in high-risk patients, which might help explain their poor prognosis.

### Improved prognostic prediction through integrative analysis of the IRG signature and clinicopathological factors

To assess whether inclusion of clinicopathological factors could increase the prognostic accuracy of our five-IRG signature’s risk score, patients’ age, gender, Fuhrman grade, and stage were accessed to perform univariate and multivariate Cox analyses in the discovery, validation, and entire TCGA sets. Tumor grade and stage were defined according to the Fuhrman classification system and the 7^th^ edition American Joint Committee on Cancer TNM classification, respectively [16]. Regression analysis results are shown in Table 3. The multivariate analysis showed that the risk score based on the IRG signature was an independent prognostic factor in the discovery set (hazard ratios (HR): 3.964; 95% CI: 1.852-8.486; *P* < 0.001), the validation set (HR: 3.957; 95% CI: 1.727-9.067; *P* = 0.001), and the entire TCGA set (HR: 3.896; 95% CI: 2.228-6.813; *P* < 0.001). Additionally, we found that age (HR: 1.030; 95% CI: 1.015-1.044; *P* < 0.001), grade (HR: 1.269; 95% CI: 0.999-1.612; *P* = 0.051), and stage (HR: 1.653; 95% CI: 1.124-2.432; *P* = 0.011) were independent prognostic factors in the entire set.

**Table 3:** Univariate and multivariate Cox regression analysis for predicting overall survival of ccRCC patients.

**Factor**	**TCGA discovery set**				**TCGA validation set**				**Entire set**			
	**Univariate**		**Multivariate**		**Univariate**		**Multivariate**		**Univariate**		**Multivariate**	
	**HR (95% CI)**	***P*-value**	**HR (95% CI)**	***P*-value**	**HR (95% CI)**	***P*-value**	**HR (95% CI)**	***P*-value**	**HR (95% CI)**	***P*-value**	**HR (95% CI)**	***P*-value**
Risk score	6.526 3.253-13.090	**<0.001**	3.964 1.852-8.486	**<0.001**	8.696 4.538-16.660	**<0.001**	3.957 1.727-9.067	**0.001**	7.714 4.799-12.400	**<0.001**	3.896 2.228-6.813	**<0.001**
Age	1.021 1.002-1.039	**0.028**	1.012 0.9918-1.033	0.243	1.032 1.014-1.051	**<0.001**	1.045 1.024-1.067	**<0.001**	1.027 1.014-1.040	**<0.001**	1.030 1.015-1.044	**<0.001**
Grade	2.111 1.568-2.841	**<0.001**	1.230 0.865-1.749	0.249	2.471 1.856-3.290	**<0.001**	1.368 0.995-1.881	0.054	2.267 1.846-2.785	**<0.001**	1.269 0.999-1.612	**0.051**
Stage	1.723 1.419-2.098	**<0.001**	1.490 1.197-1.854	**<0.001**	2.082 1.733- 2.500	**<0.001**	1.840 1.488-2.274	**<0.001**	1.874 1.641-2.140	**<0.001**	1.653 1.124-2.432	**0.011**
Gender (male vs female)	0.729 0.466-1.142	0.167	0.765 0.477-1.227	0.266	1.248 0.800-1.946	0.329	0.906 0.576-1.426	0.671	0.960 0.701-1.315	0.800	0.927 0.670-1.283	0.294

Bold values stand for *P* < 0.1.

**Figure 7:**
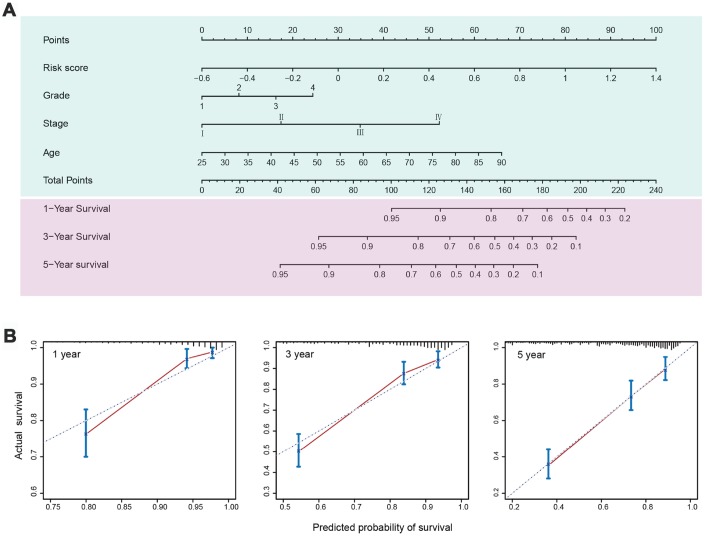
**Nomogram and calibration plots for prediction of patients’ survival in the entire TCGA set.** (**A**) Nomogram combining the five-IRG risk signature with clinical factors for prediction of 1-year, 3-year, and 5-year survival rates. (**B**) Calibration plots showing high predictive accuracy of the nomogram.

Based on the results of multivariate analysis of the entire set, C-indexes and Akaike information criterions (AICs) were calculated to evaluate the power of selected parameters, i.e. risk score, age, grade, and stage (Table 4). The combination of the five-IRG risk score with clinical factors had a higher C-index (0.772; 95% CI: 0.737-0.807) and a lower AIC than the risk score or the clinical factors alone. This indicated that combining our IRG risk score with clinical variables can improve prognostic accuracy for ccRCC. To provide a quantitative method to predict survival probability of ccRCC patients after surgery, we constructed a nomogram integrating the risk score of the IRG signature and clinical factors (Figure 7A). Calibration plots indicated that the nomogram showed good performance for predicting 1-year, 3-year, and 5-year survival probabilities (Figure 7B).

**Table 4:** Comparison of the predictive power of the prognostic models in the entire TCGA set (n = 514).

**Factor**	**Overall survival**		
	**C-index**	**95% CI**	**AIC**
Age	0.585	0.540-0.630	1880.50
Grade	0.671	0.630-0.712	1836.55
Stage	0.729	0.690-0.768	1806.80
Risk score	0.677	0.634-0.720	1834.55
Risk score + age + stage	0.769	0.734-0.804	1759.15
Age + grade + stage	0.757	0.724-0.798	1779.03
Risk score + age + grade + stage	0.772	0.737-0.807	1757.51

Abbreviations: C-index, Harrell's concordance index; CI, confidence interval; AIC, Akaike information criterion.

## DISCUSSION

Considering the importance of the immune microenvironment in neoplastic development [17], developing meaningful gene signatures to monitor the immune status of patients is significant not only to identify reliable prognostic biomarkers but, if correctly applied, to enable selection of patients at high-risk of recurrence who might benefit from additional therapy. In the present study we validated a prognostic signature, based on five-IRGs, which proved to be a reliable indicator of tumor immune status and could identify ccRCC patients with unfavorable prognosis. Moreover, our prognostic signature can further stratify ccRCC patients sharing specific clinicopathological factors (e.g., age, gender, disease grade and stage) into subgroups with different survival outcomes. Integrating these findings, we constructed a nomogram, incorporating the IRG signature’s risk score and clinical characteristics, that showed good performance for predicting survival in patients with ccRCC.

Several gene signatures representative of tumor immune status have been proposed, with potential clinical applicability in several cancers [18–20]. Although a few studies have addressed the immune microenvironment in ccRCC, and put forward immune gene-related panels with prognostic potential [21, 22], the impact of the local immune status in ccRCC progression and prognosis prediction remains to be fully explored. Association analyses combining our IRG signature’s risk score and clinicopathological parameters revealed that a higher risk score was more strongly correlated with advanced stage, higher grade and poorer prognosis. This is consistent with higher risk scores reflecting an immunosuppressive tumor microenvironment that contributes to tumor progression and recurrence. Noteworthy, we further demonstrated that the IRG signature remained an independent prognostic factor on multivariate analysis, after adjusting for clinicopathological variables. Cancer initiation and progression is often linked to a pro-inflammatory environment [23, 24]. Accordingly, dysregulated cytokine expression has been shown to contribute to the pathogenesis of RCC [25–27]. The five IRGs in our novel ccRCC signature encode cytokines or cytokine receptors with active participation in angiogenesis, chemotaxis, and inflammatory processes [23]. Accordingly, functional enrichment analysis of differentially expressed IRGs in ccRCC specimens showed that these genes are mainly involved in chemokine signaling pathways and cytokine-cytokine receptor interactions. On the other hand, GSEA showed significant alterations in immunological pathways in high-risk ccRCC patients, compared to low-risk ones. Therefore, along with previous studies, our study supports a consistent correlation between activation of inflammatory pathways, remodeling of the immune microenvironment, and ccRCC development.

Among the five IRGs, mechanistic studies of KDR, JAK3, and CXCL5 actions in ccRCC have been reported. In contrast, the impact of SHC1 and IRF7 dysregulation in ccRCC remains unclear. KDR (VEGFR-2) is overexpressed in many solid tumors and has been established as an important clinical biomarker and a key drug target in cancer research [28]. Surprisingly, the role of KDR in our signature was protective, which was further validated in the GEPIA database. This finding deserves further scrutiny, given the success of multiple VEGF receptor inhibitors in improving the prognosis of ccRCC [29]. JAK3, a cytoplasmic tyrosine kinase, is involved in the response to certain cytokines and is crucial for the function and survival of T cells [30, 31]. A role for JAK3 in the development and progression of ccRCC has been established [32]. Likewise, CXCL5 is overexpressed in ccRCC [33, 34], and based on its involvement in angiogenesis, tumor growth, and metastasis [35] has been deemed an important biomarker and a critical adjunct antiangiogenic therapy target [34]. The SHC1 gene encodes an adaptor protein that functions as a central regulator of various tyrosine kinase signaling pathways, and was proposed as a key mediator of breast cancer by promoting immune suppression [36]. Also, overexpression of SHC1 was correlated with low survival in stage IIA colon cancer [37]. In accordance with our data, a previous study indicated that SHC1, in association with dysregulated integrin expression, might be a prognostic predictor of survival in ccRCC [38]. Therefore, experimental verification of the role of SHC1 in ccRCC is clearly needed. IRF7, a central activator of the interferon type 1 immune response, has oncogenic properties and was shown to both influence tumor growth and malignant transformation in diverse tumor types [39] and to regulate myeloid-derived suppressor cell development in cancer [40]. A recent report identified IRF7 as a DEG in ccRCC [41], however, our work is the first to reveal its predictive potential as a ccRCC survival marker.

Tumor immune escape is an indispensable step to evade antitumor immune responses during cancer progression [42]. Several immunosuppressive mechanisms may be involved in the process, such as overrepresentation of immunosuppressive cells (e.g., Tregs) and increased expression of immunosuppressive molecules (e.g., PD-1, CTLA-4, LAG3, and CD47) in the tumor microenvironment. In this study, we characterized the immune cell infiltration landscapes in low-risk and high-risk ccRCC patients, and correlated our IRG signature with the expression of T-cell markers, i.e. CD4 and CD8A, and immune checkpoint genes including PD-1, PD-L1, PD-L2, CTLA-4, LAG3 [43], and CD47 [44]. Interestingly, these analyses evidenced a more immunosuppressive microenvironment in high-risk ccRCC patients, as they tended to have more T follicular helper cells, Tregs, and uncommitted (M0) macrophages, and lower abundance of NK cells, plasma cells, dendritic cells, mast cells, and eosinophils than low-risk patients. Our analysis further indicated that PD-1, CTLA-4, LAG3, and CD47 expression levels were also generally higher in high-risk ccRCC patients. These results might help explain the poor prognosis of high-risk patients, and suggest that our IRG signature may help identify those that overexpress the above markers and might hence benefit from immune checkpoint inhibitor therapies.

Previous studies confirmed that CD4^+^ T-cells can differentiate into distinct subsets with opposite functions, including blocking CD8^+^ T-cell activation and NK cell killing, recognizing cancer antigens, and aiding CD8^+^ T-cells in tumor immune responses [19, 45]. In this study, we found that high-risk ccRCC patients also tended to have more CD4^+^ and CD8^+^ T-cell infiltration rates than low-risk patients. This would suggest that the antitumor effects of high T-cell infiltration are counterbalanced by strong immunosuppressive pathways activated by overexpressed immune checkpoint proteins [46, 47]. Nevertheless, further studies are clearly needed to identify possible molecular interactions between the IRGs in our signature and cellular and molecular mediators of tumor immune status in ccRCC.

In summary, we identified a gene signature for ccRCC based on five differentially expressed IRGs, which could serve as an indicator for tumor immune status and to classify patients into two groups with distinctly different prognoses. In addition, we integrated the IRG signature with clinical factors to establish a composite prognostic nomogram for estimation of ccRCC patients’ prognosis that may be a promising tool to guide clinical practice. Limitations of our study lay in its retrospective nature, and the need to define the molecular mechanisms by which the IRGs in our signature impact ccRCC onset, progression, and outcome. Notwithstanding, besides aiding current prognostic efforts, we hope that the data presented will serve to formulate clinical studies aimed at developing novel therapeutic strategies for ccRCC.

## MATERIALS AND METHODS

### Study design and data collection

A flow chart of the study design is shown in Figure 1A. Raw data from mRNA expression microarrays profiling a total of 109 ccRCC and matched normal tissue samples were analyzed. The corresponding datasets included GSE53757 (72 pairs), GSE36895 (23 pairs), and GSE66270 (14 pairs), all downloaded from the GEO database (http://www.ncbi.nlm.nih.gov/geo/). The three GEO datasets were produced using the Affymetrix Human Genome U133 Plus 2.0 Array platform (Affymetrix, Santa Clara, CA, USA). Expression data of 72 paired ccRCC and adjacent normal tissue samples in TCGA database were obtained from UCSC Xena (https://xenabrowser.net/). The GEO and TCGA datasets were used to identify DEGs, thus increasing DEG detection reliability. IRG lists were downloaded from the ImmPort database (http://www.ncbi.nlm.nih.gov/geo/) [48] and the InnateDB database (https://www.innatedb.com/) [49], which provided a total of 1534 and 1378 human IRGs, respectively. After removal of duplicate genes, a combined gene set that included 2103 unique genes (Supplementary Table 9) was used to screen hub IRGs.

To construct an IRG signature, gene expression data of all ccRCC samples, and their associated clinical and survival information, were download from UCSC Xena. Criteria for study inclusion were: (1) Repeated tumor samples in the same patient were removed. (2) Patients with unknown survival status and follow-up information, and those who died within a follow-up period of 30 days were excluded. (3) Patients with unknown disease stage or grade were excluded. Finally, 514 patients meeting the inclusion criteria were randomly divided into two groups, the discovery set (n = 257) and the internal validation set (n = 257). The discovery set was used to establish an IRG signature to predict patients’ prognosis, while the internal validation set and the entire TCGA set were used for internal validation. The E-MTAB-3267 dataset from ArrayExpress database (https://www.ebi.ac.uk/arrayexpress/), which includes 53 ccRCC samples, was used for external validation. Detailed information is presented in Supplementary Table 10.

### Data preprocessing

For the three GEO datasets, Robust Multi-array Averaging (RMA) and the ComBat algorithm were used for background correction, data normalization, and to remove batch effects. Then, we carried out PCA and generated a heat map to visualize expression patterns and correction effects (Supplementary Figure 10); normalization was sequentially applied. For E-MTAB-3267 microarray data, RMA background correction and normalization were performed for processed signals. The “affy’’ R package and Affymetrix annotation files were used, respectively, to summarize and annotate the probes. For TCGA datasets, gene expression values were presented as the log2(x+1) transformed RSEM-normalized counts.

### Collection of clinical samples

A total of 35 matched tumor/normal tissue specimens were collected from ccRCC patients after surgery at The First Affiliated Hospital of Anhui Medical University (Hefei, China). All patients signed informed consent forms. Histological diagnosis of ccRCC was confirmed by two pathologists. Samples were immediately frozen in liquid nitrogen and stored at -80°C until RNA extraction. The study was approved by the Ethics Committee of Human Research of The First Affiliated Hospital of Anhui Medical University (No. PJ2019-14-22).

### Reverse-transcription and qRT-PCR

Total RNA from ccRCC tissues was extracted using Trizol Reagent BD (Invitrogen, Carlsbad, CA). The reverse transcription reactions were performed using a PrimeScriptTM RT reagent kit (Takara, Kusatsu, Japan) according to the manufacturer’s instructions, and qRT-PCR was prepared at a final volume of 20 μl using a SYBR Green Mix (Takara, Kusatsu, Japan) with primers synthesized by Sangon Biotech (Sangon, Shanghai, China). The reactions were measured on an ABI7500 platform (Thermo, Massachusetts, USA). The 2−ΔΔCT method was used to determine relative gene expression levels, and GAPDH was used as an internal control to normalize the data. Each reaction was performed in triplicate. The primers used for these reactions are presented in Supplementary Table 11.

### Screening of differentially expressed IRGs

Differentially expressed mRNAs between tumor and adjacent normal tissue samples were screened by the “limma” package of R software 3.4.2 (https://www.r-project.org/). |log2 fold change| > 1 and false discovery rate (FDR) < 0.05 were set as the cut-off criteria. Overlapping DEGs in paired samples from GEO and TCGA datasets, characterized by upregulation and downregulation, were extracted and intersected with an IRG set to obtain differentially expressed IRGs.

### Identification of hub IRGs

The differentially expressed IRGs were imported into the STRING database (https://string-db.org/) to construct a PPI network. Genes with a connectivity degree of > 20 and interaction score > 0.7 in the PPI were defined as hub genes for further analysis.

### Construction and validation of an IRG signature

After identifying hub IRGs, we performed LASSO Cox regression analysis [50] in the TCGA discovery set to select the best gene model for predicting prognosis in ccRCC patients. LASSO Cox regression analysis was performed using the R package “glmnet” and the optimal values of penalty parameters were determined by 10-fold cross-validation. Subsequently, we performed univariate Cox regression and survival analysis for the selected genes, and their prognostic values for overall survival were further validated using the GEPIA database (http://gepia.cancer-pku.cn/) based on available ccRCC data [51]. The IRG risk score model for each patient was determined by a linear combination of gene expression weighted by the regression coefficient from LASSO Cox regression analysis [52, 53]. Then, we used X-tile software version 3.6.1 (Yale University, New Haven, CT, USA) [54] to select the optimum cut-off value for the risk score of the IRG signature based on the patients’ survival information in the TCGA discovery set. This cut-off value was next used to divide patients into high- and low-risk groups.

To assess the predictive accuracy of the IRG signature, Harrell's concordance index (C-index) was calculated and time-dependent ROC analysis was performed. The AUC at different cut-off times was calculated using the “survival ROC” package in R [55]. K-M survival curves were used to assess survival differences between high- and low-risk groups using the “survminer” package in R. PCA was performed to assess gene expression patterns.

### Estimation of relative abundance of immune cell types

The CIBERSORT algorithm (https://cibersort.stanford.edu) [56], an approach to quantify the relative abundance of immune cell types based on specific gene expression profiles, was used to assess the distribution (normalized to 1) of 22 immune cell types in ccRCC samples from TCGA. Moreover, the P-value, correlation coefficient and root mean squared error were also presented to evaluate the accuracy of the results in each patient. A total of 500 ccRCC Patients with P-value < 0.05 were retained, and were then used to compare immune cell composition between low-risk and high-risk patients.

### Gene enrichment analysis

The Database for Annotation, Visualization and Integrated Discovery (DAVID; https://david.ncifcrf.gov/) [57] was used to perform GO and KEGG pathway analysis with the cut-off criterion of FDR < 0.01. PANTHER (http://pantherdb.org) was also used to performed pathway analysis [58]. Moreover, we performed DO analysis using the “cluster Profiler” package in R with the cut-off criteria of P < 0.05 and Q < 0.05.

### Gene set enrichment analysis (GSEA)

According to the risk score of the IRG signature, 514 ccRCC samples downloaded from TCGA were divided into high- and low-risk groups. To compare immune phenotypes between the two groups and to unmask potential functions of gene signature members, GSEA (http://software.broadinstitute.org/gsea/index.jsp) was carried out. The annotated gene set list c2.cp.kegg. v5.2.symbols.gmt was selected as the reference gene set. FDR < 0.25 and a nominal P < 0.01 were considered as cut-off criteria.

### Nomogram construction and validation

A nomogram integrating the IRG signature and various clinicopathological factors was established for predicting patients’ prognosis using the “rms” package in R [59]. Calibration curves were established to assess the accuracy of the nomogram. The C-index and AIC, representing the effect of prognosis factors, were calculated and compared to assess the predictive accuracy of the model.

### Statistical analysis

Boxplots and PCA plots were generated using the “ggplot2” package in R. The heat map was generated using the “pheatmap” package in R. Student’s t test was used to compare subgroups and paired data. Pearson’s chi-square test was applied to analyze differences between the discovery and validation sets and the association between the risk score and clinical parameters. Pearson’s correlation test was used to analyze the correlation between the IRG signature and the expression of immune checkpoint genes. K-M survival curves were compared using log-rank test. Univariate Cox regression was conducted to estimate the HR for different factors. Multivariate Cox regression was performed to determine independent factors. All statistical analyses were conducted on R software. P < 0.05 was considered significant.

## Supplementary Material

Supplementary Figures

Supplementary Tables

Supplementary Table 2

Supplementary Table 3

Supplementary Table 4

Supplementary Table 7

Supplementary Table 9

## References

[1] Ljungberg B, Bensalah K, Canfield S, Dabestani S, Hofmann F, Hora M, Kuczyk MA, Lam T, Marconi L, Merseburger AS, Mulders P, Powles T, Staehler M, et al. EAU guidelines on renal cell carcinoma: 2014 update. Eur Urol. 2015; 67:913–24. 10.1016/j.eururo.2015.01.005 25616710

[2] Hsieh JJ, Purdue MP, Signoretti S, Swanton C, Albiges L, Schmidinger M, Heng DY, Larkin J, Ficarra V. Renal cell carcinoma. Nat Rev Dis Primers. 2017; 3:17009. 10.1038/nrdp.2017.9 28276433 PMC5936048

[3] Bray F, Ferlay J, Soerjomataram I, Siegel RL, Torre LA, Jemal A. Global cancer statistics 2018: GLOBOCAN estimates of incidence and mortality worldwide for 36 cancers in 185 countries. CA Cancer J Clin. 2018; 68:394–424. 10.3322/caac.21492 30207593

[4] Pierorazio PM, Johnson MH, Patel HD, Sozio SM, Sharma R, Iyoha E, Bass EB, Allaf ME. Management of Renal Masses and Localized Renal Cancer: Systematic Review and Meta-Analysis. J Urol. 2016; 196:989–99. 10.1016/j.juro.2016.04.081 27157369 PMC5593254

[5] Motzer RJ, Haas NB, Donskov F, Gross-Goupil M, Varlamov S, Kopyltsov E, Lee JL, Melichar B, Rini BI, Choueiri TK, Zemanova M, Wood LA, Reaume MN, et al, and PROTECT investigators. Randomized Phase III Trial of Adjuvant Pazopanib Versus Placebo After Nephrectomy in Patients With Localized or Locally Advanced Renal Cell Carcinoma. J Clin Oncol. 2017; 35:3916–23. 10.1200/JCO.2017.73.5324 28902533 PMC6018511

[6] Motzer RJ, Escudier B, Oudard S, Hutson TE, Porta C, Bracarda S, Grünwald V, Thompson JA, Figlin RA, Hollaender N, Urbanowitz G, Berg WJ, Kay A, et al, and RECORD-1 Study Group. Efficacy of everolimus in advanced renal cell carcinoma: a double-blind, randomised, placebo-controlled phase III trial. Lancet. 2008; 372:449–56. 10.1016/S0140-6736(08)61039-9 18653228

[7] Weinstock M, McDermott D. Targeting PD-1/PD-L1 in the treatment of metastatic renal cell carcinoma. Ther Adv Urol. 2015; 7:365–77. 10.1177/1756287215597647 26622321 PMC4647139

[8] Ghali F, Patel SH, Derweesh IH. Current Status of Immunotherapy for Localized and Locally Advanced Renal Cell Carcinoma. J Oncol. 2019; 2019:7309205. 10.1155/2019/7309205 31057615 PMC6463563

[9] Gentles AJ, Newman AM, Liu CL, Bratman SV, Feng W, Kim D, Nair VS, Xu Y, Khuong A, Hoang CD, Diehn M, West RB, Plevritis SK, Alizadeh AA. The prognostic landscape of genes and infiltrating immune cells across human cancers. Nat Med. 2015; 21:938–45. 10.1038/nm.3909 26193342 PMC4852857

[10] Chevrier S, Levine JH, Zanotelli VRT, Silina K, Schulz D, Bacac M, Ries CH, Ailles L, Jewett MAS, Moch H, van den Broek M, Beisel C, Stadler MB, et al. An Immune Atlas of Clear Cell Renal Cell Carcinoma. Cell. 2017; 169:736–49.e18. 10.1016/j.cell.2017.04.016 28475899 PMC5422211

[11] Ghatalia P, Gordetsky J, Kuo F, Dulaimi E, Cai KQ, Devarajan K, Bae S, Naik G, Chan TA, Uzzo R, Hakimi AA, Sonpavde G, Plimack E. Prognostic impact of immune gene expression signature and tumor infiltrating immune cells in localized clear cell renal cell carcinoma. J Immunother Cancer. 2019; 7:139. 10.1186/s40425-019-0621-1 31138299 PMC6540413

[12] Frank I, Blute ML, Cheville JC, Lohse CM, Weaver AL, Zincke H. An outcome prediction model for patients with clear cell renal cell carcinoma treated with radical nephrectomy based on tumor stage, size, grade and necrosis: the SSIGN score. J Urol. 2002; 168:2395–400. 10.1016/S0022-5347(05)64153-5 12441925

[13] Brooks SA, Brannon AR, Parker JS, Fisher JC, Sen O, Kattan MW, Hakimi AA, Hsieh JJ, Choueiri TK, Tamboli P, Maranchie JK, Hinds P, Miller CR, et al. ClearCode34: A prognostic risk predictor for localized clear cell renal cell carcinoma. Eur Urol. 2014; 66:77–84. 10.1016/j.eururo.2014.02.035 24613583 PMC4058355

[14] Rini B, Goddard A, Knezevic D, Maddala T, Zhou M, Aydin H, Campbell S, Elson P, Koscielny S, Lopatin M, Svedman C, Martini JF, Williams JA, et al. A 16-gene assay to predict recurrence after surgery in localised renal cell carcinoma: development and validation studies. Lancet Oncol. 2015; 16:676–85. 10.1016/S1470-2045(15)70167-1 25979595

[15] Wu J, Jin S, Gu W, Wan F, Zhang H, Shi G, Qu Y, Ye D. Construction and Validation of a 9-Gene Signature for Predicting Prognosis in Stage III Clear Cell Renal Cell Carcinoma. Front Oncol. 2019; 9:152. 10.3389/fonc.2019.00152 30941304 PMC6433707

[16] Edge SB, Compton CC. The American Joint Committee on Cancer: the 7th edition of the AJCC cancer staging manual and the future of TNM. Ann Surg Oncol. 2010; 17:1471–4. 10.1245/s10434-010-0985-4 20180029

[17] Chen DS, Mellman I. Elements of cancer immunity and the cancer-immune set point. Nature. 2017; 541:321–30. 10.1038/nature21349 28102259

[18] Li B, Cui Y, Diehn M, Li R. Development and Validation of an Individualized Immune Prognostic Signature in Early-Stage Nonsquamous Non-Small Cell Lung Cancer. JAMA Oncol. 2017; 3:1529–37. 10.1001/jamaoncol.2017.1609 28687838 PMC5710196

[19] Long J, Wang A, Bai Y, Lin J, Yang X, Wang D, Yang X, Jiang Y, Zhao H. Development and validation of a TP53-associated immune prognostic model for hepatocellular carcinoma. EBioMedicine. 2019; 42:363–74. 10.1016/j.ebiom.2019.03.022 30885723 PMC6491941

[20] Wang Z, Song Q, Yang Z, Chen J, Shang J, Ju W. Construction of immune-related risk signature for renal papillary cell carcinoma. Cancer Med. 2019; 8:289–304. 10.1002/cam4.1905 30516029 PMC6346237

[21] Geissler K, Fornara P, Lautenschläger C, Holzhausen HJ, Seliger B, Riemann D. Immune signature of tumor infiltrating immune cells in renal cancer. Oncoimmunology. 2015; 4:e985082. 10.4161/2162402X.2014.985082 25949868 PMC4368143

[22] Giraldo NA, Becht E, Vano Y, Petitprez F, Lacroix L, Validire P, Sanchez-Salas R, Ingels A, Oudard S, Moatti A, Buttard B, Bourass S, Germain C, et al. Tumor-Infiltrating and Peripheral Blood T-cell Immunophenotypes Predict Early Relapse in Localized Clear Cell Renal Cell Carcinoma. Clin Cancer Res. 2017; 23:4416–28. 10.1158/1078-0432.CCR-16-2848 28213366

[23] Lin WW, Karin M. A cytokine-mediated link between innate immunity, inflammation, and cancer. J Clin Invest. 2007; 117:1175–83. 10.1172/JCI31537 17476347 PMC1857251

[24] Lippitz BE. Cytokine patterns in patients with cancer: a systematic review. Lancet Oncol. 2013; 14:e218–28. 10.1016/S1470-2045(12)70582-X 23639322

[25] Romero JM, Aptsiauri N, Vazquez F, Cozar JM, Canton J, Cabrera T, Tallada M, Garrido F, Ruiz-Cabello F. Analysis of the expression of HLA class I, proinflammatory cytokines and chemokines in primary tumors from patients with localized and metastatic renal cell carcinoma. Tissue Antigens. 2006; 68:303–10. 10.1111/j.1399-0039.2006.00673.x 17026465

[26] Cabillic F, Bouet-Toussaint F, Toutirais O, Rioux-Leclercq N, Fergelot P, de la Pintière CT, Genetet N, Patard JJ, Catros-Quemener V. Interleukin-6 and vascular endothelial growth factor release by renal cell carcinoma cells impedes lymphocyte-dendritic cell cross-talk. Clin Exp Immunol. 2006; 146:518–23. 10.1111/j.1365-2249.2006.03212.x 17100773 PMC1810419

[27] Alberti L, Thomachot MC, Bachelot T, Menetrier-Caux C, Puisieux I, Blay JY. IL-6 as an intracrine growth factor for renal carcinoma cell lines. Int J Cancer. 2004; 111:653–61. 10.1002/ijc.20287 15252833

[28] Rapisarda A, Melillo G. Role of the VEGF/VEGFR axis in cancer biology and therapy. Adv Cancer Res. 2012; 114:237–67. 10.1016/B978-0-12-386503-8.00006-5 22588059

[29] Lee SH, Jeong D, Han YS, Baek MJ. Pivotal role of vascular endothelial growth factor pathway in tumor angiogenesis. Ann Surg Treat Res. 2015; 89:1–8. 10.4174/astr.2015.89.1.1 26131438 PMC4481026

[30] Kolenko V, Wang Q, Riedy MC, O’Shea J, Ritz J, Cathcart MK, Rayman P, Tubbs R, Edinger M, Novick A, Bukowski R, Finke J. Tumor-induced suppression of T lymphocyte proliferation coincides with inhibition of Jak3 expression and IL-2 receptor signaling: role of soluble products from human renal cell carcinomas. J Immunol. 1997; 159:3057–67. 9300731

[31] Gigante M, Pontrelli P, Herr W, Gigante M, D’Avenia M, Zaza G, Cavalcanti E, Accetturo M, Lucarelli G, Carrieri G, Battaglia M, Storkus WJ, Gesualdo L, Ranieri E. miR-29b and miR-198 overexpression in CD8+ T cells of renal cell carcinoma patients down-modulates JAK3 and MCL-1 leading to immune dysfunction. J Transl Med. 2016; 14:84. 10.1186/s12967-016-0841-9 27063186 PMC4827202

[32] de Martino M, Gigante M, Cormio L, Prattichizzo C, Cavalcanti E, Gigante M, Ariano V, Netti GS, Montemurno E, Mancini V, Battaglia M, Gesualdo L, Carrieri G, Ranieri E. JAK3 in clear cell renal cell carcinoma: mutational screening and clinical implications. Urol Oncol. 2013; 31:930–37. 10.1016/j.urolonc.2011.07.001 21868263

[33] Begley LA, Kasina S, Mehra R, Adsule S, Admon AJ, Lonigro RJ, Chinnaiyan AM, Macoska JA. CXCL5 promotes prostate cancer progression. Neoplasia. 2008; 10:244–54. 10.1593/neo.07976 18320069 PMC2262133

[34] Li A, King J, Moro A, Sugi MD, Dawson DW, Kaplan J, Li G, Lu X, Strieter RM, Burdick M, Go VL, Reber HA, Eibl G, Hines OJ. Overexpression of CXCL5 is associated with poor survival in patients with pancreatic cancer. Am J Pathol. 2011; 178:1340–49. 10.1016/j.ajpath.2010.11.058 21356384 PMC3069811

[35] Guan Z, Li C, Fan J, He D, Li L. Androgen receptor (AR) signaling promotes RCC progression via increased endothelial cell proliferation and recruitment by modulating AKT → NF-κB → CXCL5 signaling. Sci Rep. 2016; 6:37085. 10.1038/srep37085 27848972 PMC5111066

[36] Ahn R, Sabourin V, Bolt AM, Hébert S, Totten S, De Jay N, Festa MC, Young YK, Im YK, Pawson T, Koromilas AE, Muller WJ, Mann KK, et al. The Shc1 adaptor simultaneously balances Stat1 and Stat3 activity to promote breast cancer immune suppression. Nat Commun. 2017; 8:14638. 10.1038/ncomms14638 28276425 PMC5347092

[37] Grossman SR, Lyle S, Resnick MB, Sabo E, Lis RT, Rosinha E, Liu Q, Hsieh CC, Bhat G, Frackelton AR Jr, Hafer LJ. p66 Shc tumor levels show a strong prognostic correlation with disease outcome in stage IIA colon cancer. Clin Cancer Res. 2007; 13:5798–804. 10.1158/1078-0432.CCR-07-0073 17908971

[38] Lu X, Wan F, Zhang H, Shi G, Ye D. ITGA2B and ITGA8 are predictive of prognosis in clear cell renal cell carcinoma patients. Tumour Biol. 2016; 37:253–62. 10.1007/s13277-015-3792-5 26198048

[39] Yanai H, Negishi H, Taniguchi T. The IRF family of transcription factors: Inception, impact and implications in oncogenesis. Oncoimmunology. 2012; 1:1376–86. 10.4161/onci.22475 23243601 PMC3518510

[40] Yang Q, Li X, Chen H, Cao Y, Xiao Q, He Y, Wei J, Zhou J. IRF7 regulates the development of granulocytic myeloid-derived suppressor cells through S100A9 transrepression in cancer. Oncogene. 2017; 36:2969–80. 10.1038/onc.2016.448 28092673

[41] Wang Y, Wei H, Song L, Xu L, Bao J, Liu J. Gene Expression Microarray Data Meta-Analysis Identifies Candidate Genes and Molecular Mechanism Associated with Clear Cell Renal Cell Carcinoma. Cell J. 2020; 22:386–93. 10.22074/cellj.2020.6561 31863665 PMC6947001

[42] Dunn GP, Bruce AT, Ikeda H, Old LJ, Schreiber RD. Cancer immunoediting: from immunosurveillance to tumor escape. Nat Immunol. 2002; 3:991–98. 10.1038/ni1102-991 12407406

[43] Woo SR, Turnis ME, Goldberg MV, Bankoti J, Selby M, Nirschl CJ, Bettini ML, Gravano DM, Vogel P, Liu CL, Tangsombatvisit S, Grosso JF, Netto G, et al. Immune inhibitory molecules LAG-3 and PD-1 synergistically regulate T-cell function to promote tumoral immune escape. Cancer Res. 2012; 72:917–27. 10.1158/0008-5472.CAN-11-1620 22186141 PMC3288154

[44] Casey SC, Tong L, Li Y, Do R, Walz S, Fitzgerald KN, Gouw AM, Baylot V, Gütgemann I, Eilers M, Felsher DW. MYC regulates the antitumor immune response through CD47 and PD-L1. Science. 2016; 352:227–31. 10.1126/science.aac9935 26966191 PMC4940030

[45] Pinto MP, Balmaceda C, Bravo ML, Kato S, Villarroel A, Owen GI, Roa JC, Cuello MA, Ibañez C. Patient inflammatory status and CD4+/CD8+ intraepithelial tumor lymphocyte infiltration are predictors of outcomes in high-grade serous ovarian cancer. Gynecol Oncol. 2018; 151:10–17. 10.1016/j.ygyno.2018.07.025 30078505

[46] Matsushita H, Sato Y, Karasaki T, Nakagawa T, Kume H, Ogawa S, Homma Y, Kakimi K. Neoantigen Load, Antigen Presentation Machinery, and Immune Signatures Determine Prognosis in Clear Cell Renal Cell Carcinoma. Cancer Immunol Res. 2016; 4:463–71. 10.1158/2326-6066.CIR-15-0225 26980598

[47] Chen YP, Zhang Y, Lv JW, Li YQ, Wang YQ, He QM, Yang XJ, Sun Y, Mao YP, Yun JP, Liu N, Ma J. Genomic Analysis of Tumor Microenvironment Immune Types across 14 Solid Cancer Types: immunotherapeutic Implications. Theranostics. 2017; 7:3585–94. 10.7150/thno.21471 28912897 PMC5596445

[48] Bhattacharya S, Andorf S, Gomes L, Dunn P, Schaefer H, Pontius J, Berger P, Desborough V, Smith T, Campbell J, Thomson E, Monteiro R, Guimaraes P, et al. ImmPort: disseminating data to the public for the future of immunology. Immunol Res. 2014; 58:234–39. 10.1007/s12026-014-8516-1 24791905

[49] Breuer K, Foroushani AK, Laird MR, Chen C, Sribnaia A, Lo R, Winsor GL, Hancock RE, Brinkman FS, Lynn DJ. InnateDB: systems biology of innate immunity and beyond—recent updates and continuing curation. Nucleic Acids Res. 2013; 41:D1228–33. 10.1093/nar/gks1147 23180781 PMC3531080

[50] Tibshirani R. The lasso method for variable selection in the Cox model. Stat Med. 1997; 16:385–95. 10.1002/(SICI)1097-0258(19970228)16:4<385::AID-SIM380>3.0.CO;2-3 9044528

[51] Tang Z, Li C, Kang B, Gao G, Li C, Zhang Z. GEPIA: a web server for cancer and normal gene expression profiling and interactive analyses. Nucleic Acids Res. 2017; 45:W98–102. 10.1093/nar/gkx247 28407145 PMC5570223

[52] Lossos IS, Czerwinski DK, Alizadeh AA, Wechser MA, Tibshirani R, Botstein D, Levy R. Prediction of survival in diffuse large-B-cell lymphoma based on the expression of six genes. N Engl J Med. 2004; 350:1828–37. 10.1056/NEJMoa032520 15115829

[53] Chen HY, Yu SL, Chen CH, Chang GC, Chen CY, Yuan A, Cheng CL, Wang CH, Terng HJ, Kao SF, Chan WK, Li HN, Liu CC, et al. A five-gene signature and clinical outcome in non-small-cell lung cancer. N Engl J Med. 2007; 356:11–20. 10.1056/NEJMoa060096 17202451

[54] Camp RL, Dolled-Filhart M, Rimm DL. X-tile: a new bio-informatics tool for biomarker assessment and outcome-based cut-point optimization. Clin Cancer Res. 2004; 10:7252–59. 10.1158/1078-0432.CCR-04-0713 15534099

[55] Heagerty PJ, Lumley T, Pepe MS. Time-dependent ROC curves for censored survival data and a diagnostic marker. Biometrics. 2000; 56:337–44. 10.1111/j.0006-341X.2000.00337.x 10877287

[56] Newman AM, Liu CL, Green MR, Gentles AJ, Feng W, Xu Y, Hoang CD, Diehn M, Alizadeh AA. Robust enumeration of cell subsets from tissue expression profiles. Nat Methods. 2015; 12:453–57. 10.1038/nmeth.3337 25822800 PMC4739640

[57] Huang W, Sherman BT, Lempicki RA. Systematic and integrative analysis of large gene lists using DAVID bioinformatics resources. Nat Protoc. 2009; 4:44–57. 10.1038/nprot.2008.211 19131956

[58] Mi H, Muruganujan A, Thomas PD. PANTHER in 2013: modeling the evolution of gene function, and other gene attributes, in the context of phylogenetic trees. Nucleic Acids Res. 2013; 41:D377–86. 10.1093/nar/gks1118 23193289 PMC3531194

[59] Balachandran VP, Gonen M, Smith JJ, DeMatteo RP. Nomograms in oncology: more than meets the eye. Lancet Oncol. 2015; 16:e173–80. 10.1016/S1470-2045(14)71116-7 25846097 PMC4465353

