# Supplementary material for: Identification of an immune-related risk signature for predicting prognosis in clear cell renal cell carcinoma
Source: Aging (Albany NY). 2020 Feb 6;12(3):2302–32. doi: 10.18632/aging.102746 (PMC7041771; doi:10.18632/aging.102746)
Supplement: Supplementary Figures [file aging-12-102746-s007..pdf]

SUPPLEMENTARY FIGURES

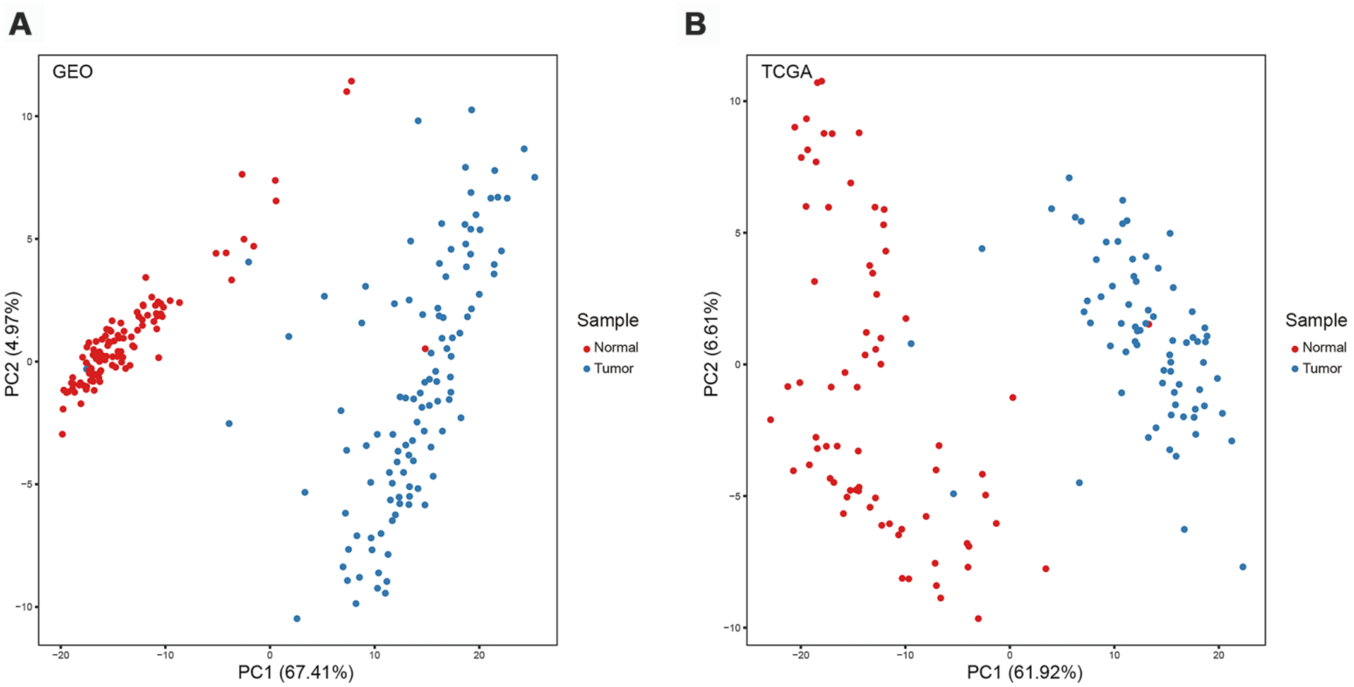

Supplementary Figure 1. Principal component analysis highlighting different immune phenotypes in normal and ccRCC tissue samples. (**A**) Samples from the GEO database. (**B**) Samples from the TCGA database.

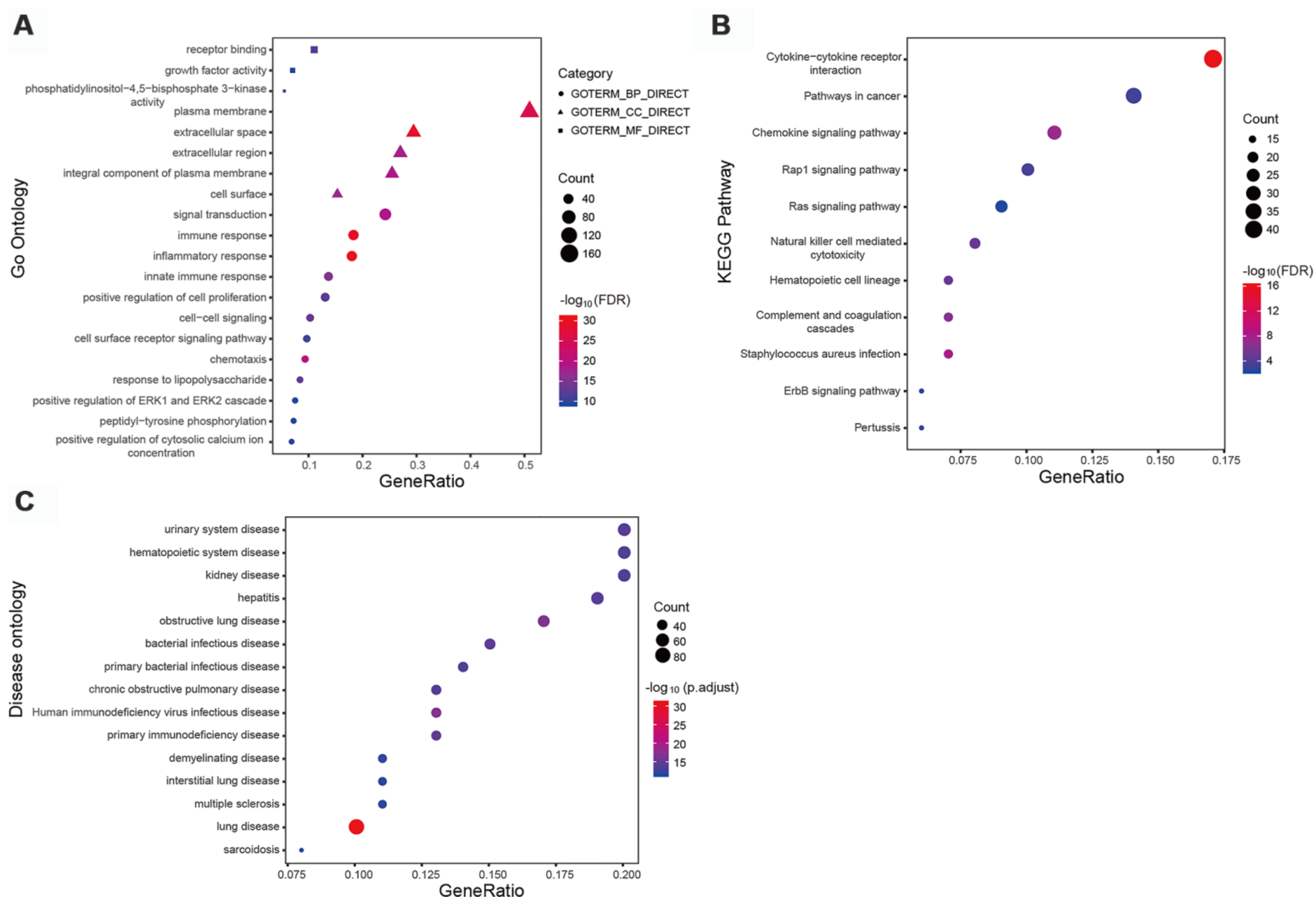

Supplementary Figure 2. Functional enrichment of 326 differentially expressed IRGs. (**A**) Top 20 most significant Gene Ontology (GO) terms. (**B**) Kyoto Encyclopedia of Genes and Genomes pathway analysis. FDR < 0.01 indicated significant enrichment. (**C**) Top 15 most significant disease ontology (DO) terms identified by DO analysis.

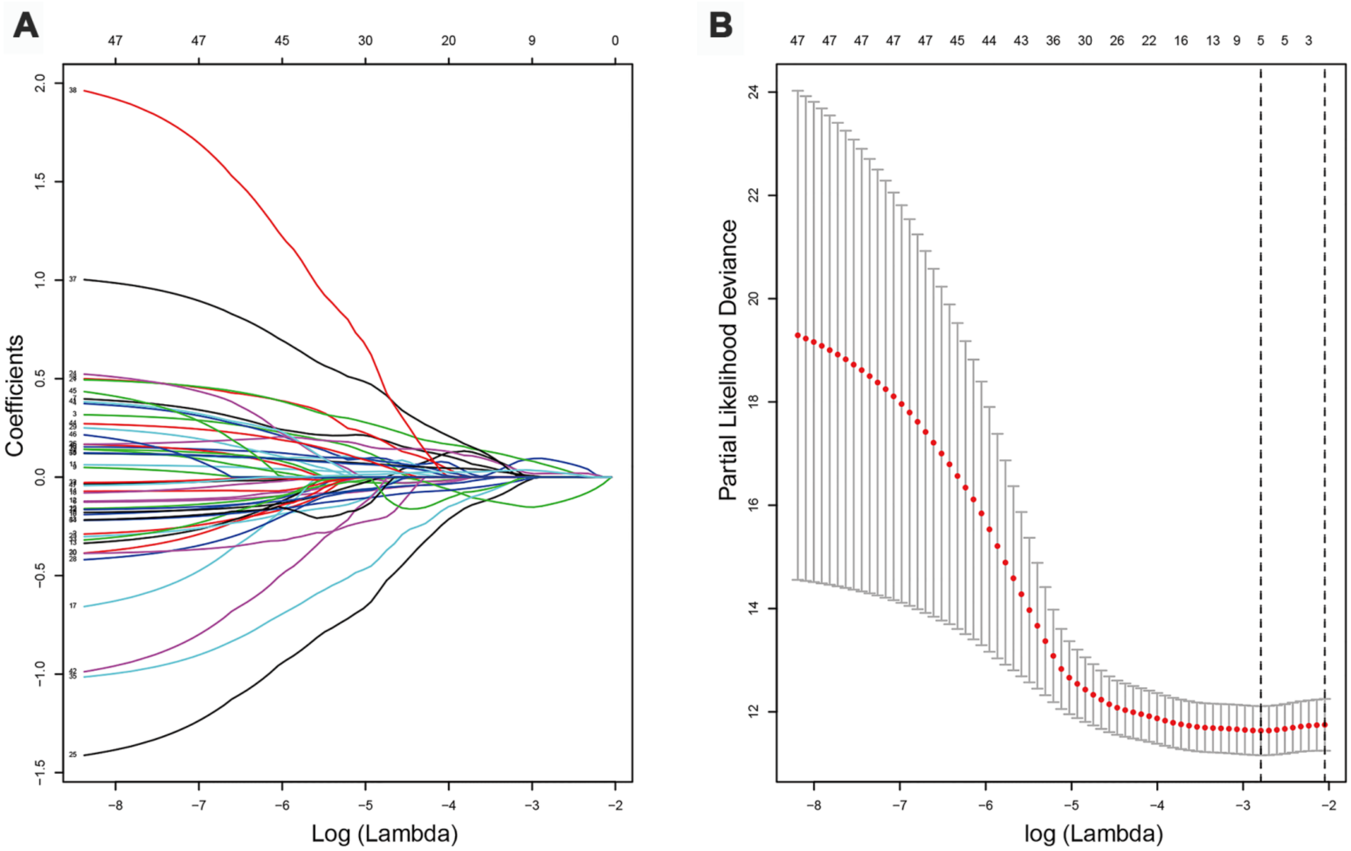

Supplementary Figure 3. LASSO Cox regression model. (**A**) Plot of LASSO coefficient profiles. (**B**) Plot of partial likelihood deviance for the 47 immune-related hub genes in the TCGA discovery set.

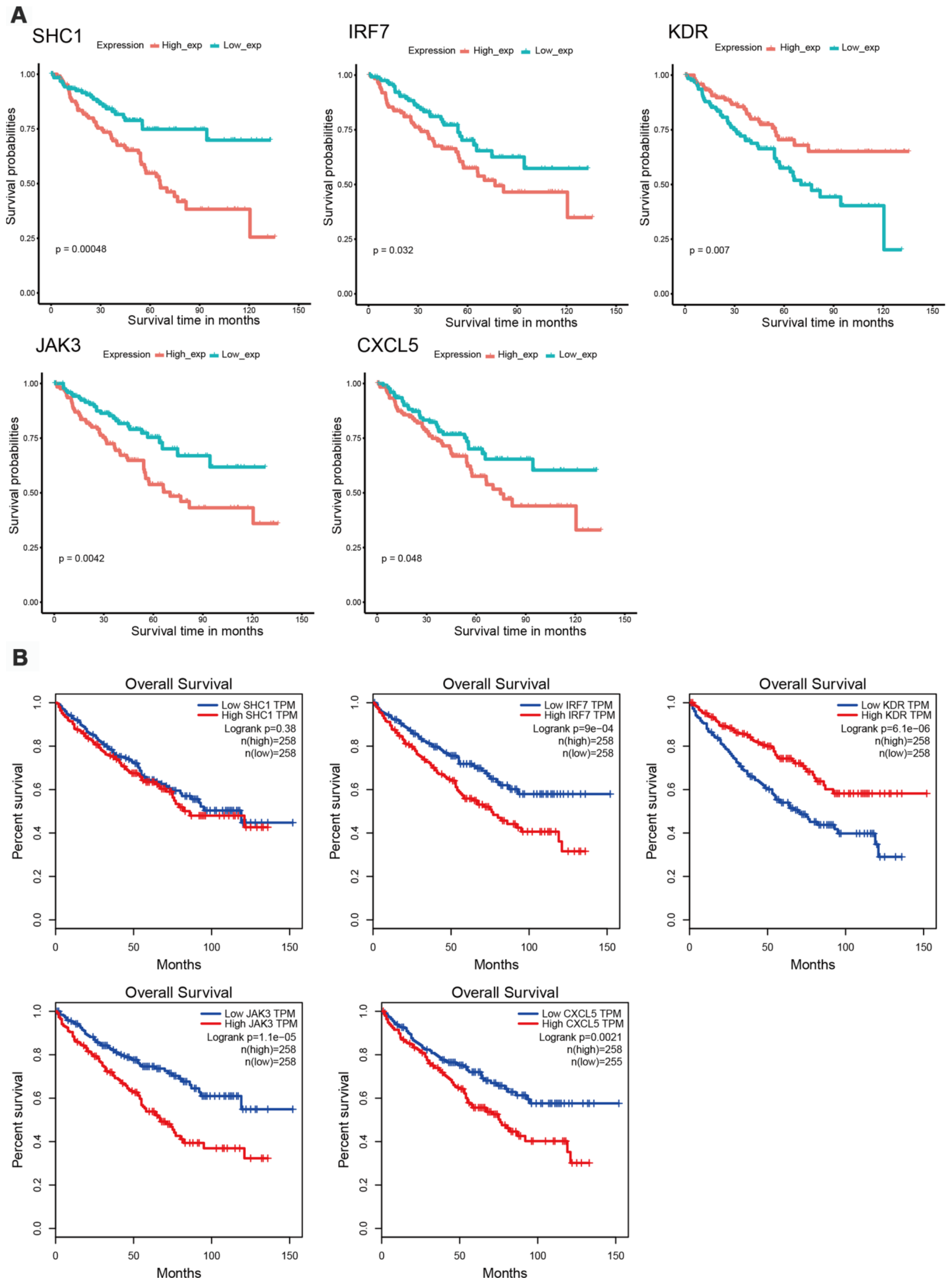

Supplementary Figure 4. Kaplan-Meier analysis of the 5 genes used to construct the immune-related risk signature for ccRCC. (**A**) Analysis in the TCGA discovery set. (**B**) Further validation in the GEPIA database.

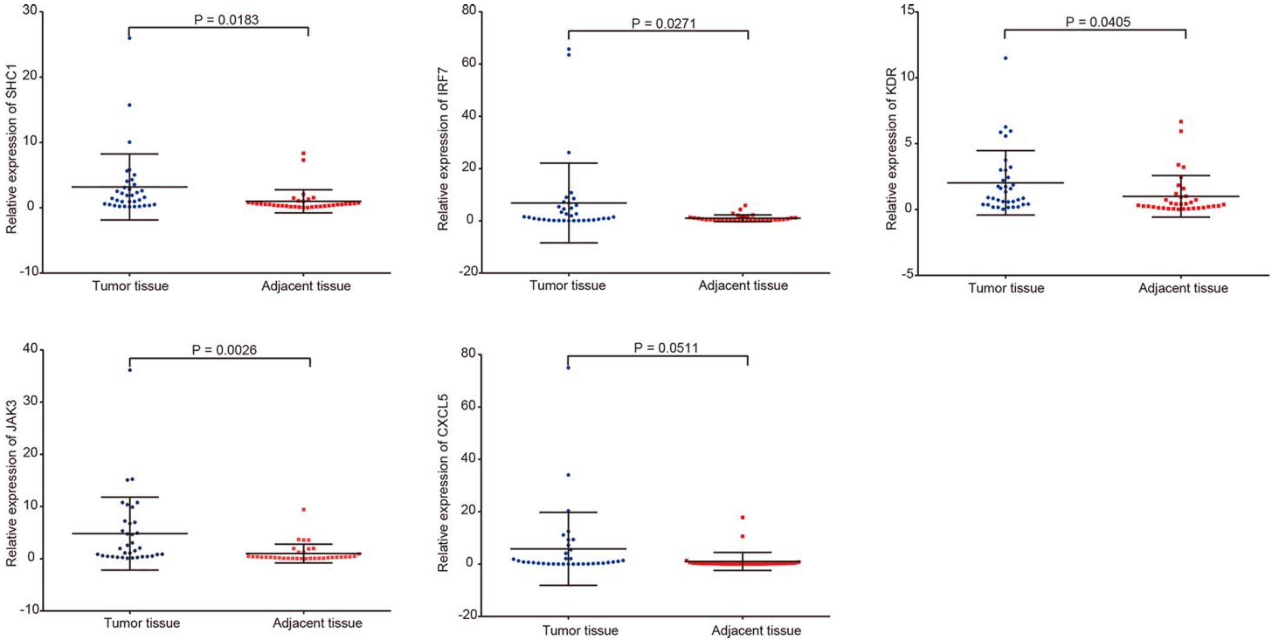

Supplementary Figure 5. Validation of the five-IRG signature in matched normal and ccRCC specimens harvested at The First Affiliated Hospital of Anhui Medical University (n = 35). In agreement with results from the other databases analyzed, the five genes were up-regulated in ccRCC samples compared with adjacent, normal ones.

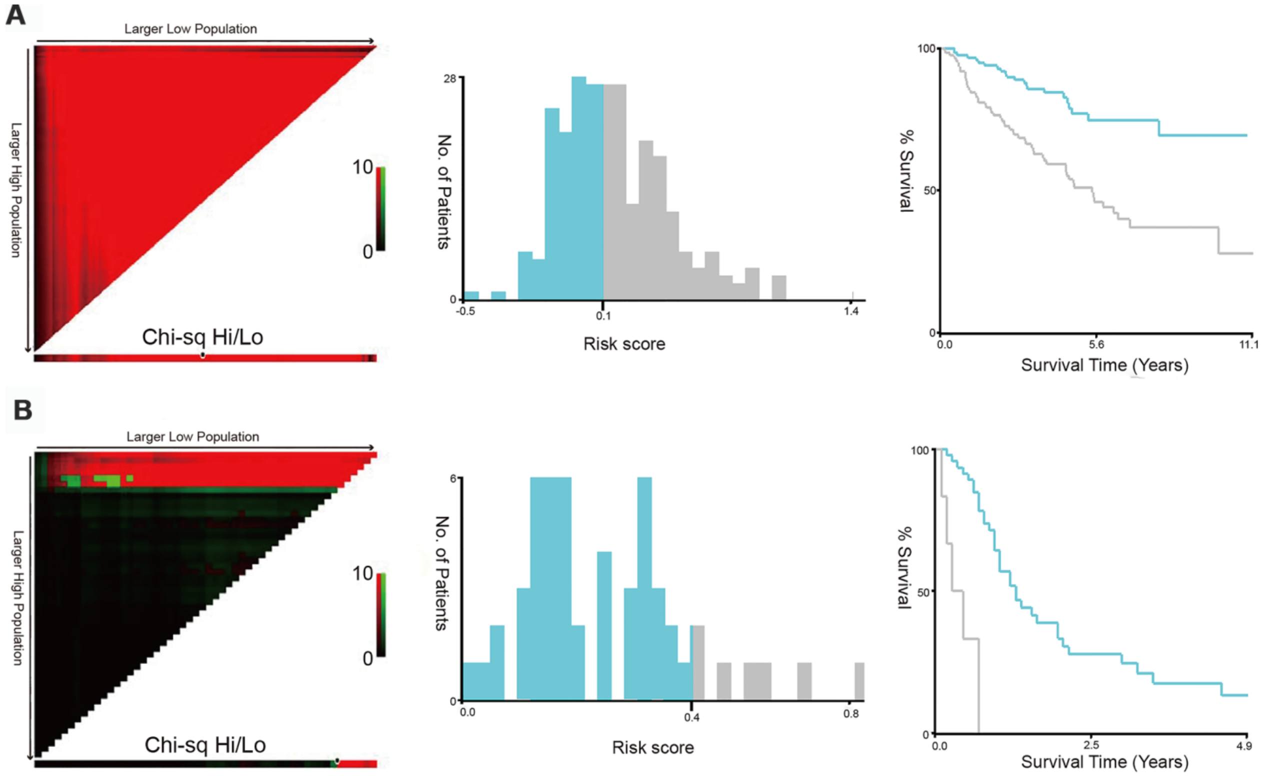

Supplementary Figure 6. X-tile analysis for selection of optimum cut-off value for the IRG signature's risk score. Left panels indicate an inverse association (red) between the risk score and overall survival. Middle panels show risk score distribution. Right panels show Kaplan-Meier survival plots for high-risk and low-risk groups. (**A**) Analysis of the TCGA discovery set. (**B**) Analysis of the E-MTAB-3267 dataset.

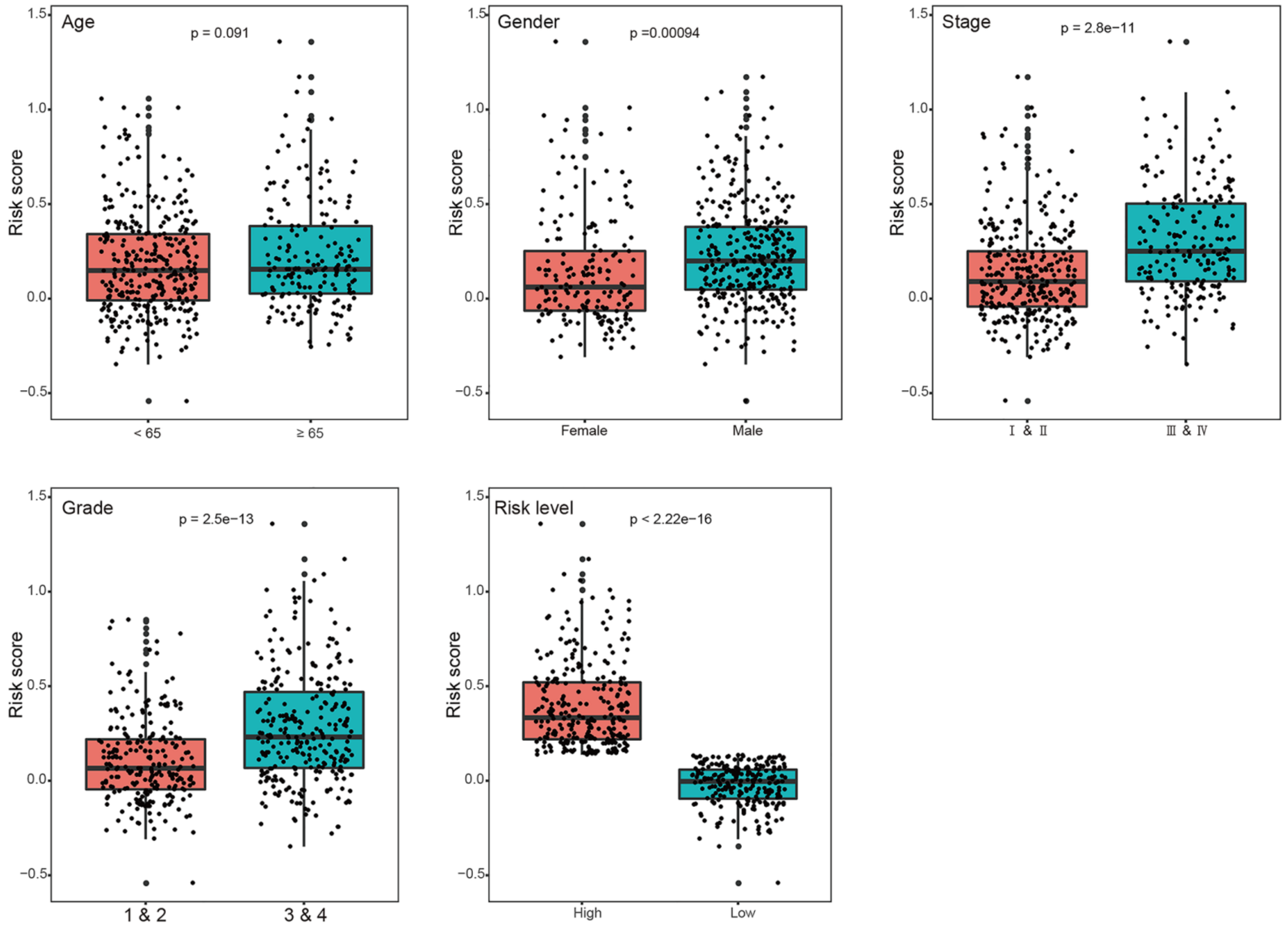

Supplementary Figure 7. Correlation between clinical factors and IRG signature's risk score. The bar chart shows a trend toward higher risk score for male gender, advanced stage, high grade, and high-risk classification groups.

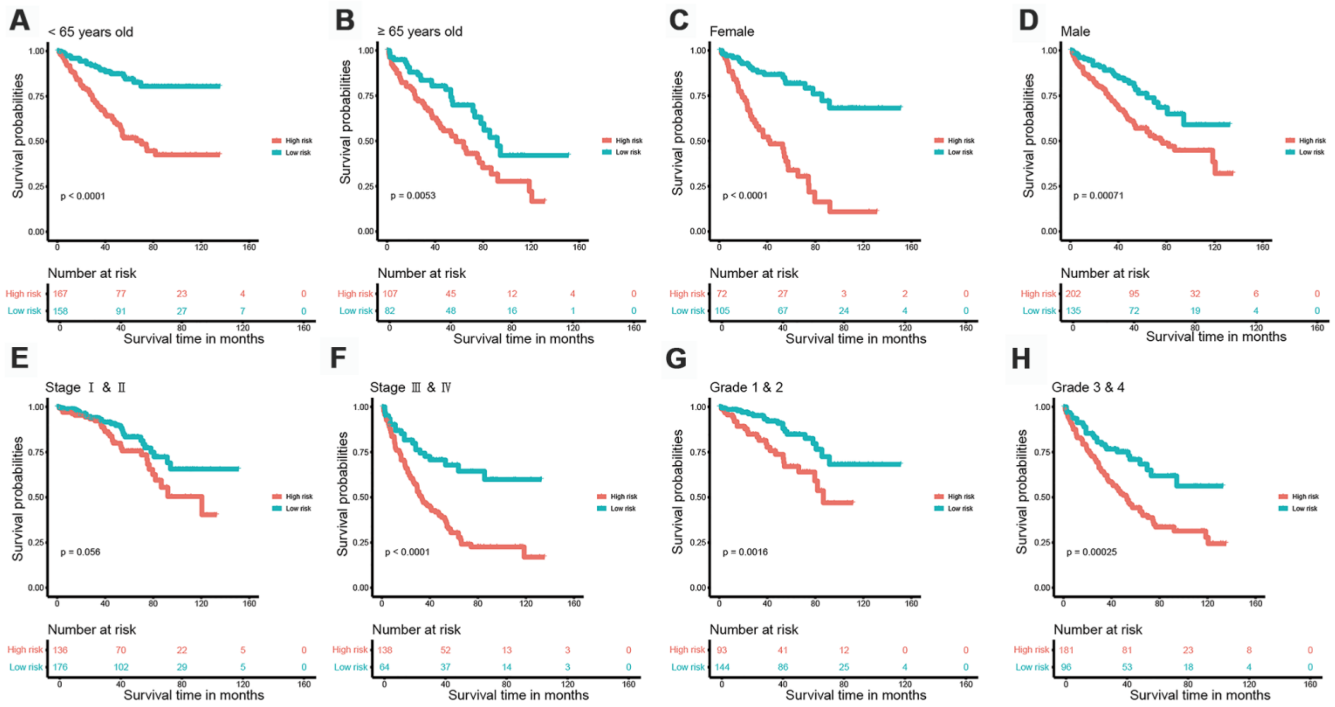

Supplementary Figure 8. IRG signature-based sub-group analysis. Kaplan-Meier analysis of the risk signature in patients (**A**) aged < 65 years, (**B**) aged ≥ 65 years, (**C**) female, (**D**) male, (**E**) stage I & II, (**F**) stage III & IV, (**G**) grade 1 & 2, and (**H**) grade 3 & 4.

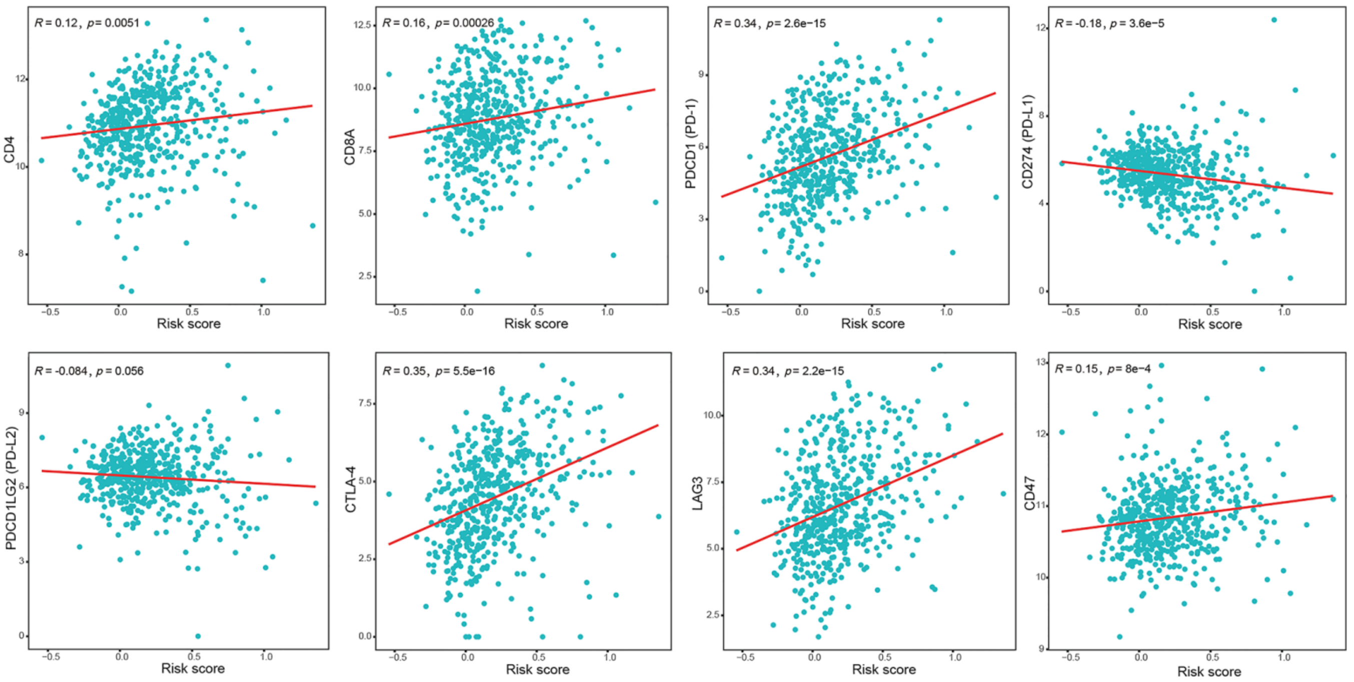

Supplementary Figure 9. Pearson's correlation analysis of the risk score of the signature and the expression of T-cell and immune checkpoint genes.

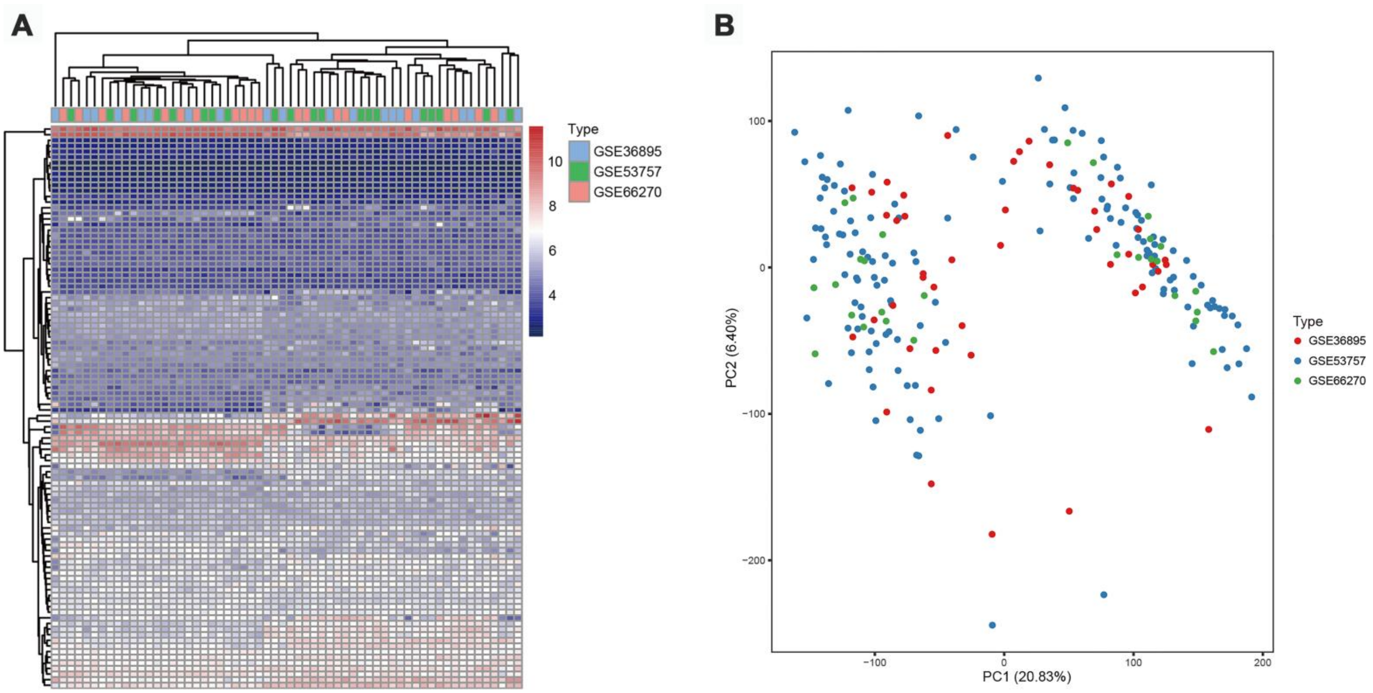

Supplementary Figure 10. Assessment of correction effects for the 3 GEO datasets. (**A**) A heat map based on the top 100 genes from 20 random samples in the 3 datasets shows no distinctly different sample clustering in each dataset. (**B**) Principal component analysis showing random sample distribution in the 3 datasets.
