# Supplementary material for: Identification of an immune-related risk signature for predicting prognosis in clear cell renal cell carcinoma
Source: Aging (Albany NY). 2020 Feb 6;12(3):2302–32. doi: 10.18632/aging.102746 (PMC7041771; doi:10.18632/aging.102746)
Supplement: Supplementary Tables [file aging-12-102746-s006..pdf]

SUPPLEMENTARY TABLES

Supplementary Table 1. Differently expressed immune-related genes between normal and ccRCC samples.

KIT	ADRB1	C1QB	LCP2	CD300LF
FGFR2	C5	BIRC3	LY86	CFD
AQP3	NR1I3	CXCL11	FCGR3B	LAT2
NKIRAS1	TAC1	INHBB	EGFR	C1S
BTC	FREM1	SCARB1	HCK	ERAP2
ERBB2	GP2	CTSS	IL20RB	IFI27
AMFR	ANGPTL1	IDO1	SLC15A4	BTN3A3
NR2F1	VDR	CXCL10	PYCARD	IL7
PGR	KLK1	SEMA5B	CCL28	ITGAM
VAV3	OLFM4	TRIB3	ADCY7	TRIM22
GDF7	ACAA1	CXCL9	GJA1	GMFG
PTGER1	UCHL1	C1QC	MYC	ITK
CTNNAL1	USP2	MSR1	LAIR1	CD48
PLCG2	CHGB	CCL20	ISG20	SAMHD1
MUC1	KL	TLR8	CCL4	IL27RA
SDC1	HSPA2	TNFSF13B	LGALS1	CD200
FGFR3	PTGER3	STC2	TGFA	CD247
CAT	PDE1A	TRIM9	CSF2RB	TNFAIP3
CR1	SLIT2	ITGB2	TAP1	NLRC3
IRF6	CR2	CD86	PSMB8	EDNRA
CXCL12	SEMA6D	TYROBP	EDN1	AREG
SEMA3G	PDGFRA	TLR3	RUNX3	RARRES2
SORT1	BMPR1B	PTPRC	JAK3	PIK3R5
NUDT6	DCN	C1QA	RAC2	MEF2C
PRKAR2B	GATA3	TLR7	TGFB1	ELMO1
PLXNB1	PLCL1	SLAMF8	CX3CR1	CCR2
RORB	PTH1R	APOBEC3G	CD70	IL2RB
CYFIP2	DEFB1	GBP2	FCGR2B	CCDC88A
AVPR1A	APOH	CCL5	CXCL5	CLEC5A
PALM3	ESRRG	ADM	CD37	RHBDF2
NR0B2	FAM3B	LY96	TREM2	LST1
PRKCA	PRLR	FCER1G	PDGFD	OAS2
KITLG	C7	ABCG1	GZMB	ETS1
TRIM50	ANGPTL3	OSMR	NCF2	C5AR1
PELI2	AZGP1	CD1D	APOL1	NR5A2
TYRO3	FGF1	BTN3A2	VEGFA	SERPINB9
TRIM71	FGF9	CD300A	TYMP	GRK5
CMTM4	EGF	BCL2A1	CD14	ICAM1
TGFBR3	ERBB4	SLAMF7	TCF4	TNFRSF1B
AGR2	PLG	AXL	C3AR1	FPR1
SIGIRR	HRG	CD8A	LCK	ANGPTL2
BPI	ANGPTL4	CD53	PRF1	CCNA2
F11	C3	IL10RA	TLR2	ARHGAP15
TNFSF15	CD36	CXCL13	SIGLEC10	NOD2
PAK6	CXCR4	IFI16	FLI1	CSF1R
THRB	CAV1	CORO1A	PROCR	TAPBP
SEMA3B	PTHLH	CARD16	ANXA1	IRF7
PPARGC1A	PDK1	SERPINE1	CCR5	ISG15
CTSH	P2RX7	CLEC7A	MICB	BTN3A1
NR3C2	ESM1	CASP1	NLRC5	MET
CXCL16	CCL18	CD3D	BIRC5	IL4R
PLXND1	FLT1	TNFSF9	NCKAP1L	NLRC4
PLXNC1	MCHR1	IL12RB1	TRPV2	CMTM3
NMB	TNFRSF9	MASP1	TNFRSF10B	TRIM55
CSF2RA	CYSLTR1	HMOX1	DUSP4	RFTN1
SIRPA	SCG2	UBE2L6	CEBPB	WIPF1
FSTL1	JAG2	AKNA	BST2	FGR
CYTIP	INPP5D	IRF1	VAV1	NRP2
CD72	CD27	LGALS9	CASP4	LRRK2
ANXA4	AIM2	S100A8	CALCRL	SDC3
KDR	VEGFC	FZD1	IL16	S100A9
IL15RA	NRG3	S100A10	CD4	BMP2

ABCA1	APLN	CCRL2	NFKBIE	IL18R1
REG1A	TRAT1	OAS3	SEMA6A	DUSP10
IL7R	FAS	IL32	PLAUR	NR3C1
SHC1				

Please browse Full Text version to see the data of Supplementary Tables 2 to 4

Supplementary Table 2. Results of GO analysis based on 326 differentially expressed immune-related genes.

Supplementary Table 3. PANTHER pathway analysis based on 326 differentially expressed immune-related genes.

Supplementary Table 4. Disease ontology analysis based on 326 differentially expressed immune-related genes.

Supplementary Table 5. Results of GO analysis based on 47 immune-related hub genes.

ID	Description	Count	GeneRatio	FDR
GO:0006954	inflammatory response	23	0.489362	2.36E-21
GO:0006935	chemotaxis	17	0.361702	2.73E-20
GO:0008009	chemokine activity	12	0.255319	5.49E-16
GO:0070098	chemokine-mediated signaling pathway	13	0.276596	6.49E-16
GO:0006955	immune response	18	0.382979	5.11E-13
GO:0060326	cell chemotaxis	11	0.234043	2.04E-12
GO:0007186	G-protein coupled receptor signaling pathway	21	0.446809	6.97E-11
GO:0007204	positive regulation of cytosolic calcium ion concentration	11	0.234043	3.65E-09
GO:0005615	extracellular space	21	0.446809	1.98E-08
GO:0009986	cell surface	15	0.319149	3.94E-08
GO:0007267	cell-cell signaling	12	0.255319	9.81E-08
GO:0030593	neutrophil chemotaxis	8	0.170213	7.61E-07
GO:0007165	signal transduction	19	0.404255	7.73E-07
GO:0050900	leukocyte migration	9	0.191489	1.96E-06
GO:0005886	plasma membrane	30	0.638298	5.27E-06
GO:0032496	response to lipopolysaccharide	9	0.191489	2.04E-05
GO:0005576	extracellular region	19	0.404255	2.73E-05
GO:0070374	positive regulation of ERK1 and ERK2 cascade	9	0.191489	3.39E-05
GO:0045766	positive regulation of angiogenesis	8	0.170213	3.87E-05
GO:0008284	positive regulation of cell proliferation	12	0.255319	5.70E-05
GO:0007166	cell surface receptor signaling pathway	10	0.212766	7.17E-05
GO:0048248	CXCR3 chemokine receptor binding	4	0.085106	2.28E-04
GO:0005102	receptor binding	10	0.212766	4.65E-04
GO:0048015	phosphatidylinositol-mediated signaling	7	0.148936	6.43E-04
GO:0046934	phosphatidylinositol-4,5-bisphosphate 3-kinase activity	6	0.12766	8.38E-04
GO:0071222	cellular response to lipopolysaccharide	7	0.148936	9.38E-04
GO:0009897	external side of plasma membrane	8	0.170213	0.001079
GO:0006968	cellular defense response	6	0.12766	0.00109
GO:0004713	protein tyrosine kinase activity	7	0.148936	0.001869
GO:0008360	regulation of cell shape	7	0.148936	0.003297
GO:0014066	regulation of phosphatidylinositol 3-kinase signaling	6	0.12766	0.003443
GO:2000406	positive regulation of T cell migration	4	0.085106	0.003497
GO:0018108	peptidyl-tyrosine phosphorylation	7	0.148936	0.00552
GO:0005887	integral component of plasma membrane	15	0.319149	0.006709
GO:0046854	phosphatidylinositol phosphorylation	6	0.12766	0.008666
GO:0007169	transmembrane receptor protein tyrosine kinase signaling pathway	6	0.12766	0.009611
GO:0009986	cell surface	15	0.319149	3.94E-08
GO:0007267	cell-cell signaling	12	0.255319	9.81E-08
GO:0030593	neutrophil chemotaxis	8	0.170213	7.61E-07

Supplementary Table 6. PANTHER pathway analysis based on 47 immune-related hub genes.

Pathway Accession	Pathway Name	Components	Subfamilies	Associated Sequence
P00005	Angiogenesis	77	246	1254
P00026	Heterotrimeric G-protein signaling pathway-Gi alpha and Gs alpha mediated pathway	27	217	1104
P04394	Thyrotropin-releasing hormone receptor signaling pathway	9	95	535
P00048	PI3 kinase pathway	35	78	425
P05916	Opioid prodynorphin pathway	12	47	303
P05913	Enkephalin release	10	47	314
P05917	Opioid proopiomelanocortin pathway	9	46	309
P00038	JAK/STAT signaling pathway	8	18	113
P00047	PDGF signaling pathway	36	214	1032
P05915	Opioid proenkephalin pathway	11	46	302
P00036	Interleukin signaling pathway	36	90	555
P00012	Cadherin signaling pathway	16	239	1058
P00034	Integrin signalling pathway	46	251	1296
P06959	CCKR signaling map	290	171	171
P04385	Histamine H1 receptor mediated signaling pathway	5	66	395
P00019	Endothelin signaling pathway	27	149	727
P00031	Inflammation mediated by chemokine and cytokine signaling pathway	58	353	1652
P00054	Toll receptor signaling pathway	46	72	308
P00021	FGF signaling pathway	26	236	1135
P04374	5HT2 type receptor mediated signaling pathway	9	103	569
P00009	Axon guidance mediated by netrin	11	47	228
P04393	Ras Pathway	39	104	602
P04376	5HT4 type receptor mediated signaling pathway	7	47	287
P04391	Oxytocin receptor mediated signaling pathway	6	88	515
P00018	EGF receptor signaling pathway	28	278	1161
P05731	GABA-B_receptor_II_signaling	9	41	238
P00008	Axon guidance mediated by Slit/Robo	14	39	173
P04377	Beta1 adrenergic receptor signaling pathway	7	64	392
P05912	Dopamine receptor mediated signaling pathway	27	85	422
P04378	Beta2 adrenergic receptor signaling pathway	7	64	393
P00010	B cell activation	37	80	428
P04379	Beta3 adrenergic receptor signaling pathway	4	41	257
P00049	Parkinson disease	37	212	1128
P00056	VEGF signaling pathway	25	98	491
P06664	Gonadotropin releasing hormone receptor pathway	216	233	235
P04386	Histamine H2 receptor mediated signaling pathway	4	39	233
P00053	T cell activation	45	97	561
P00057	Wnt signaling pathway	49	483	2308
P04373	5HT1 type receptor mediated signaling pathway	8	62	390

Please browse Full Text version to see the data of Supplementary Table 7

Supplementary Table 7. Disease ontology analysis based on 47 immune-related hub genes.

Supplementary Table 8. Basic characteristics of the five immune-related genes.

Gene	Description	Function	Cytoband
SHC1	SHC (Src homology 2 domain containing) transforming protein 1	ephrin receptor binding	1q21.3
IRF7	interferon regulatory factor 7	protein binding	11p15.5
KDR	kinase insert domain receptor	transferase activity	4q12
JAK3	Janus kinase 3	transferase activity	19p13.11
CXCL5	chemokine (C-X-C motif) ligand 5	chemokine activity	4q13.3

Please browse Full Text version to see the data of Supplementary Table 9

Supplementary Table 9. Immune gene sets from ImmPort and InnateDB databases.

Supplementary Table 10. Clinical characteristics of patients in each dataset.

Variables	Discovery set n (%)	Internal validation set n (%)	*P* value	E-MTAB-3267 n (%)
Age (mean ± SD, years)	60.1 ± 12.3	60.8 ± 12.0	0.498	59.8 ± 8.2
Age			0.464	
≥ 65	90 (35.0)	99 (38.5)		15
< 65	167 (65.0)	158 (61.5)		38
Gender			1	
Male	168 (65.4)	169 (65.8)		37
Female	89 (34.6)	88 (34.2)		16
Stage			0.797	
I	125 (48.6)	133 (51.8)		NA
II	29 (11.3)	25 (9.7)		NA
III	59 (23.0)	61 (23.7)		NA
IV	44 (17.1)	38 (14.8)		NA
Grade			0.988	
G1	7 (2.7)	6 (2.3)		NA
G2	113 (44.0)	111 (43.2)		NA
G3	101 (39.3)	103 (40.1)		NA
G4	36 (14.0)	37 (14.4)		NA
Status			0.453	
Dead	80 (31.1)	89 (34.6)		39*
Alive	177 (68.9)	168 (65.4)		14**

*progression ** no-progression

Supplementary Table 11. Primers for q-RT-PCR analysis of the 5 IRGs.

Symbol	Forward primer	Reverse primer
SHC1	5'-ATCACTCTCACCGTCTCCACCAG-3'	5'-TCTTTGGCAACATAGGCGACATACTC-3'
IRF7	5'-CCCACGCTATACCATCTACCT-3'	5'-GATGTCGTCATAGAGGCTGTTG-3'
KDR	5'-GGAGCTTAAGAATGCATCCTTG-3'	5'-GATGCTTTCCCCAATACTTGTC-3'
JAK3	5'-CCTGATCGTGGTCCAGAGAG-3'	5'-GCAGGGATCTTGTGAAATGTCAT-3'
CXCL5	5'-ATCAGTAATCTGCAAGTGTTCG-3'	5'-CAAGACAAATTTCCTTCCCGTT-3'
GAPDH	5'-CAGGAGGCATTGCTGATGAT-3'	5'-GAAGGCTGGGGCTCATTT-3'
