# Supplementary material for: Identification of an immune-related risk signature for predicting prognosis in clear cell renal cell carcinoma
Source: Aging (Albany NY). 2020 Feb 6;12(3):2302–32. doi: 10.18632/aging.102746 (PMC7041771; doi:10.18632/aging.102746)
Supplement: Supplementary Table 2 [file aging-12-102746-s002..docx]

Supplementary Table 2. Results of GO analysis based on 326 differentially expressed immune-related genes.

| ID | Description | Count | GeneRatio | FDR |
| --- | --- | --- | --- | --- |
| GO:0006954 | inflammatory response | 58 | 0.177914 | 1.44E-31 |
| GO:0006955 | immune response | 59 | 0.180982 | 4.75E-30 |
| GO:0005615 | extracellular space | 96 | 0.294479 | 8.64E-30 |
| GO:0005886 | plasma membrane | 166 | 0.509202 | 2.93E-26 |
| GO:0006935 | chemotaxis | 30 | 0.092025 | 1.23E-20 |
| GO:0007165 | signal transduction | 78 | 0.239264 | 1.37E-19 |
| GO:0005887 | integral component of plasma membrane | 83 | 0.254601 | 2.41E-19 |
| GO:0005576 | extracellular region | 88 | 0.269939 | 9.41E-19 |
| GO:0009986 | cell surface | 50 | 0.153374 | 4.31E-18 |
| GO:0045087 | innate immune response | 44 | 0.134969 | 5.12E-16 |
| GO:0007267 | cell-cell signaling | 33 | 0.101227 | 3.12E-14 |
| GO:0032496 | response to lipopolysaccharide | 27 | 0.082822 | 2.00E-13 |
| GO:0008284 | positive regulation of cell proliferation | 42 | 0.128834 | 4.00E-13 |
| GO:0005102 | receptor binding | 36 | 0.110429 | 9.77E-13 |
| GO:0046934 | phosphatidylinositol-4,5-bisphosphate 3-kinase activity | 18 | 0.055215 | 1.31E-12 |
| GO:0007166 | cell surface receptor signaling pathway | 31 | 0.095092 | 1.77E-11 |
| GO:0007204 | positive regulation of cytosolic calcium ion concentration | 22 | 0.067485 | 1.75E-10 |
| GO:0018108 | peptidyl-tyrosine phosphorylation | 23 | 0.070552 | 2.77E-10 |
| GO:0008083 | growth factor activity | 23 | 0.070552 | 4.99E-10 |
| GO:0070374 | positive regulation of ERK1 and ERK2 cascade | 24 | 0.07362 | 5.44E-10 |
| GO:0004713 | protein tyrosine kinase activity | 21 | 0.064417 | 8.64E-10 |
| GO:0014066 | regulation of phosphatidylinositol 3-kinase signaling | 17 | 0.052147 | 2.19E-09 |
| GO:0046854 | phosphatidylinositol phosphorylation | 18 | 0.055215 | 3.75E-09 |
| GO:0005515 | protein binding | 225 | 0.690184 | 3.80E-09 |
| GO:0005088 | Ras guanyl-nucleotide exchange factor activity | 19 | 0.058282 | 6.33E-09 |
| GO:0009897 | external side of plasma membrane | 24 | 0.07362 | 6.50E-09 |
| GO:0042803 | protein homodimerization activity | 45 | 0.138037 | 8.38E-09 |
| GO:0045766 | positive regulation of angiogenesis | 19 | 0.058282 | 1.08E-08 |
| GO:0000165 | MAPK cascade | 27 | 0.082822 | 1.11E-08 |
| GO:0030335 | positive regulation of cell migration | 23 | 0.070552 | 1.24E-08 |
| GO:0004872 | receptor activity | 24 | 0.07362 | 2.73E-08 |
| GO:0048015 | phosphatidylinositol-mediated signaling | 18 | 0.055215 | 2.83E-08 |
| GO:0007169 | transmembrane receptor protein tyrosine kinase signaling pathway | 17 | 0.052147 | 6.18E-08 |
| GO:0071222 | cellular response to lipopolysaccharide | 18 | 0.055215 | 8.12E-08 |
| GO:0001525 | angiogenesis | 24 | 0.07362 | 8.77E-08 |
| GO:0051607 | defense response to virus | 21 | 0.064417 | 8.94E-08 |
| GO:0004714 | transmembrane receptor protein tyrosine kinase activity | 12 | 0.03681 | 9.09E-08 |
| GO:0070098 | chemokine-mediated signaling pathway | 15 | 0.046012 | 1.01E-07 |
| GO:0008009 | chemokine activity | 13 | 0.039877 | 1.06E-07 |
| GO:0043406 | positive regulation of MAP kinase activity | 14 | 0.042945 | 1.12E-07 |
| GO:0005125 | cytokine activity | 21 | 0.064417 | 1.67E-07 |
| GO:0045121 | membrane raft | 22 | 0.067485 | 1.67E-07 |
| GO:0006915 | apoptotic process | 37 | 0.113497 | 4.39E-07 |
| GO:0001948 | glycoprotein binding | 13 | 0.039877 | 3.55E-06 |
| GO:0070062 | extracellular exosome | 92 | 0.282209 | 4.44E-06 |
| GO:0060326 | cell chemotaxis | 13 | 0.039877 | 5.41E-06 |
| GO:0030593 | neutrophil chemotaxis | 13 | 0.039877 | 6.50E-06 |
| GO:0050679 | positive regulation of epithelial cell proliferation | 12 | 0.03681 | 2.69E-05 |
| GO:0006968 | cellular defense response | 12 | 0.03681 | 3.85E-05 |
| GO:0060337 | type I interferon signaling pathway | 12 | 0.03681 | 5.44E-05 |
| GO:0050776 | regulation of immune response | 18 | 0.055215 | 9.70E-05 |
| GO:0003707 | steroid hormone receptor activity | 11 | 0.033742 | 1.09E-04 |
| GO:0043552 | positive regulation of phosphatidylinositol 3-kinase activity | 9 | 0.027607 | 1.52E-04 |
| GO:0048010 | vascular endothelial growth factor receptor signaling pathway | 12 | 0.03681 | 1.93E-04 |
| GO:0050729 | positive regulation of inflammatory response | 12 | 0.03681 | 2.24E-04 |
| GO:0043547 | positive regulation of GTPase activity | 32 | 0.09816 | 2.40E-04 |
| GO:0009615 | response to virus | 14 | 0.042945 | 3.07E-04 |
| GO:0001666 | response to hypoxia | 17 | 0.052147 | 3.45E-04 |
| GO:0051781 | positive regulation of cell division | 10 | 0.030675 | 3.79E-04 |
| GO:0043235 | receptor complex | 14 | 0.042945 | 5.70E-04 |
| GO:0014068 | positive regulation of phosphatidylinositol 3-kinase signaling | 11 | 0.033742 | 6.90E-04 |
| GO:0004716 | receptor signaling protein tyrosine kinase activity | 6 | 0.018405 | 7.54E-04 |
| GO:0042127 | regulation of cell proliferation | 17 | 0.052147 | 9.34E-04 |
| GO:0001618 | virus receptor activity | 11 | 0.033742 | 9.64E-04 |
| GO:0050900 | leukocyte migration | 14 | 0.042945 | 0.001038 |
| GO:0043066 | negative regulation of apoptotic process | 27 | 0.082822 | 0.001199 |
| GO:0010628 | positive regulation of gene expression | 20 | 0.06135 | 0.001242 |
| GO:0000187 | activation of MAPK activity | 13 | 0.039877 | 0.001629 |
| GO:0001934 | positive regulation of protein phosphorylation | 14 | 0.042945 | 0.00165 |
| GO:0016064 | immunoglobulin mediated immune response | 6 | 0.018405 | 0.001819 |
| GO:0005154 | epidermal growth factor receptor binding | 8 | 0.02454 | 0.001945 |
| GO:0016020 | membrane | 70 | 0.214724 | 0.002041 |
| GO:0043401 | steroid hormone mediated signaling pathway | 10 | 0.030675 | 0.002118 |
| GO:0030217 | T cell differentiation | 8 | 0.02454 | 0.00212 |
| GO:0008201 | heparin binding | 15 | 0.046012 | 0.002733 |
| GO:0051092 | positive regulation of NF-kappaB transcription factor activity | 14 | 0.042945 | 0.002796 |
| GO:0019835 | cytolysis | 7 | 0.021472 | 0.003459 |
| GO:0010595 | positive regulation of endothelial cell migration | 9 | 0.027607 | 0.003931 |
| GO:0004888 | transmembrane signaling receptor activity | 17 | 0.052147 | 0.004065 |
| GO:0071526 | semaphorin-plexin signaling pathway | 8 | 0.02454 | 0.004235 |
| GO:0008360 | regulation of cell shape | 14 | 0.042945 | 0.004988 |
| GO:0043410 | positive regulation of MAPK cascade | 11 | 0.033742 | 0.005529 |
| GO:0004896 | cytokine receptor activity | 8 | 0.02454 | 0.005698 |
| GO:0042981 | regulation of apoptotic process | 17 | 0.052147 | 0.006033 |
| GO:0006919 | activation of cysteine-type endopeptidase activity involved in apoptotic process | 11 | 0.033742 | 0.006922 |
| GO:0002250 | adaptive immune response | 14 | 0.042945 | 0.009255 |
| GO:0050852 | T cell receptor signaling pathway | 14 | 0.042945 | 0.009255 |
| GO:0046777 | protein autophosphorylation | 15 | 0.046012 | 0.009835 |
