# Supplementary material for: Identification of an immune-related risk signature for predicting prognosis in clear cell renal cell carcinoma
Source: Aging (Albany NY). 2020 Feb 6;12(3):2302–32. doi: 10.18632/aging.102746 (PMC7041771; doi:10.18632/aging.102746)
Supplement: Supplementary Table 3 [file aging-12-102746-s001..docx]

Supplementary Table 3. PANTHER pathway analysis based on 326 differentially expressed immune-related genes.

| Pathway Accession | Pathway Name | Components | Subfamilies | Associated Sequence |
| --- | --- | --- | --- | --- |
| P00005 | Angiogenesis | 77 | 246 | 1254 |
| P00011 | Blood coagulation | 59 | 47 | 206 |
| P00052 | TGF-beta signaling pathway | 20 | 168 | 830 |
| P00026 | Heterotrimeric G-protein signaling pathway-Gi alpha and Gs alpha mediated pathway | 27 | 217 | 1104 |
| P00059 | p53 pathway | 70 | 107 | 591 |
| P04394 | Thyrotropin-releasing hormone receptor signaling pathway | 9 | 95 | 535 |
| P00048 | PI3 kinase pathway | 35 | 78 | 425 |
| P05913 | Enkephalin release | 10 | 47 | 314 |
| P05917 | Opioid proopiomelanocortin pathway | 9 | 46 | 309 |
| P00038 | JAK/STAT signaling pathway | 8 | 18 | 113 |
| P00025 | Hedgehog signaling pathway | 20 | 37 | 171 |
| P05911 | Angiotensin II-stimulated signaling through G proteins and beta-arrestin | 21 | 40 | 261 |
| P00050 | Plasminogen activating cascade | 22 | 19 | 82 |
| P00036 | Interleukin signaling pathway | 36 | 90 | 555 |
| P00016 | Cytoskeletal regulation by Rho GTPase | 20 | 174 | 824 |
| P00019 | Endothelin signaling pathway | 27 | 149 | 727 |
| P00031 | Inflammation mediated by chemokine and cytokine signaling pathway | 58 | 353 | 1652 |
| P00021 | FGF signaling pathway | 26 | 236 | 1135 |
| P00009 | Axon guidance mediated by netrin | 11 | 47 | 228 |
| P00002 | Alpha adrenergic receptor signaling pathway | 6 | 34 | 158 |
| P04393 | Ras Pathway | 39 | 104 | 602 |
| P00007 | Axon guidance mediated by semaphorins | 14 | 25 | 138 |
| P04391 | Oxytocin receptor mediated signaling pathway | 6 | 88 | 515 |
| P00008 | Axon guidance mediated by Slit/Robo | 14 | 39 | 173 |
| P00027 | Heterotrimeric G-protein signaling pathway-Gq alpha and Go alpha mediated pathway | 21 | 154 | 837 |
| P05912 | Dopamine receptor mediated signaling pathway | 27 | 85 | 422 |
| P00046 | Oxidative stress response | 20 | 80 | 358 |
| P04378 | Beta2 adrenergic receptor signaling pathway | 7 | 64 | 393 |
| P00003 | Alzheimer disease-amyloid secretase pathway | 31 | 118 | 532 |
| P00010 | B cell activation | 37 | 80 | 428 |
| P00049 | Parkinson disease | 37 | 212 | 1128 |
| P04379 | Beta3 adrenergic receptor signaling pathway | 4 | 41 | 257 |
| P06664 | Gonadotropin releasing hormone receptor pathway | 216 | 233 | 235 |
| P00056 | VEGF signaling pathway | 25 | 98 | 491 |
| P00029 | Huntington disease | 60 | 306 | 1510 |
| P05918 | p38 MAPK pathway | 36 | 47 | 228 |
| P00020 | FAS signaling pathway | 31 | 51 | 244 |
| P00006 | Apoptosis signaling pathway | 72 | 171 | 844 |
| P04386 | Histamine H2 receptor mediated signaling pathway | 4 | 39 | 233 |
| P00057 | Wnt signaling pathway | 49 | 483 | 2308 |
| P00053 | T cell activation | 45 | 97 | 561 |
| P04373 | 5HT1 type receptor mediated signaling pathway | 8 | 62 | 390 |
| P00033 | Insulin/IGF pathway-protein kinase B signaling cascade | 18 | 55 | 286 |
| P04398 | p53 pathway feedback loops 2 | 32 | 67 | 412 |
| P00039 | Metabotropic glutamate receptor group III pathway | 12 | 103 | 560 |
| P05916 | Opioid prodynorphin pathway | 12 | 47 | 303 |
| P00047 | PDGF signaling pathway | 36 | 214 | 1032 |
| P04396 | Vitamin D metabolism and pathway | 11 | 17 | 86 |
| P05915 | Opioid proenkephalin pathway | 11 | 46 | 302 |
| P00042 | Muscarinic acetylcholine receptor 1 and 3 signaling pathway | 12 | 97 | 508 |
| P00012 | Cadherin signaling pathway | 16 | 239 | 1058 |
| P06959 | CCKR signaling map | 290 | 171 | 171 |
| P00034 | Integrin signalling pathway | 46 | 251 | 1296 |
| P04385 | Histamine H1 receptor mediated signaling pathway | 5 | 66 | 395 |
| P00043 | Muscarinic acetylcholine receptor 2 and 4 signaling pathway | 11 | 114 | 524 |
| P00060 | Ubiquitin proteasome pathway | 6 | 127 | 565 |
| P00054 | Toll receptor signaling pathway | 46 | 72 | 308 |
| P04374 | 5HT2 type receptor mediated signaling pathway | 9 | 103 | 569 |
| P00004 | Alzheimer disease-presenilin pathway | 70 | 210 | 1042 |
| P04376 | 5HT4 type receptor mediated signaling pathway | 7 | 47 | 287 |
| P00018 | EGF receptor signaling pathway | 28 | 278 | 1161 |
| P05731 | GABA-B_receptor_II_signaling | 9 | 41 | 238 |
| P00055 | Transcription regulation by bZIP transcription factor | 25 | 89 | 509 |
| P04377 | Beta1 adrenergic receptor signaling pathway | 7 | 64 | 392 |
| P00040 | Metabotropic glutamate receptor group II pathway | 7 | 73 | 392 |
| P00045 | Notch signaling pathway | 23 | 68 | 275 |
