# Supplementary material for: Identification of an immune-related risk signature for predicting prognosis in clear cell renal cell carcinoma
Source: Aging (Albany NY). 2020 Feb 6;12(3):2302–32. doi: 10.18632/aging.102746 (PMC7041771; doi:10.18632/aging.102746)
Supplement: Supplementary Table 4 [file aging-12-102746-s004..docx]

Supplementary Table 4. Disease ontology analysis based on 326 differentially expressed immune-related genes.

| ID | Description | GeneRatio | p.adjust | Count |
| --- | --- | --- | --- | --- |
| DOID:850 | lung disease | 82/282 | 1.25E-31 | 82 |
| DOID:526 | Human immunodeficiency virus infectious disease | 38/282 | 9.39E-18 | 38 |
| DOID:2320 | obstructive lung disease | 49/282 | 3.12E-17 | 49 |
| DOID:612 | primary immunodeficiency disease | 37/282 | 3.43E-15 | 37 |
| DOID:104 | bacterial infectious disease | 43/282 | 4.78E-15 | 43 |
| DOID:2237 | hepatitis | 54/282 | 1.11E-14 | 54 |
| DOID:18 | urinary system disease | 57/282 | 1.12E-14 | 57 |
| DOID:3083 | chronic obstructive pulmonary disease | 38/282 | 1.39E-14 | 38 |
| DOID:74 | hematopoietic system disease | 56/282 | 2.63E-14 | 56 |
| DOID:0050338 | primary bacterial infectious disease | 39/282 | 2.70E-14 | 39 |
| DOID:557 | kidney disease | 55/282 | 2.70E-14 | 55 |
| DOID:3213 | demyelinating disease | 32/282 | 5.33E-13 | 32 |
| DOID:2377 | multiple sclerosis | 31/282 | 1.22E-12 | 31 |
| DOID:3082 | interstitial lung disease | 31/282 | 1.35E-12 | 31 |
| DOID:11335 | sarcoidosis | 22/282 | 3.80E-12 | 22 |
| DOID:3856 | male reproductive organ cancer | 49/282 | 9.55E-12 | 49 |
| DOID:2916 | hypersensitivity reaction type IV disease | 22/282 | 9.55E-12 | 22 |
| DOID:10283 | prostate cancer | 48/282 | 1.42E-11 | 48 |
| DOID:4451 | renal carcinoma | 45/282 | 1.53E-11 | 45 |
| DOID:3770 | pulmonary fibrosis | 25/282 | 2.79E-11 | 25 |
| DOID:1247 | blood coagulation disease | 30/282 | 2.79E-11 | 30 |
| DOID:3996 | urinary system cancer | 52/282 | 2.79E-11 | 52 |
| DOID:2789 | parasitic protozoa infectious disease | 25/282 | 5.20E-11 | 25 |
| DOID:1398 | parasitic infectious disease | 27/282 | 5.76E-11 | 27 |
| DOID:4481 | allergic rhinitis | 22/282 | 8.64E-11 | 22 |
| DOID:263 | kidney cancer | 47/282 | 1.09E-10 | 47 |
| DOID:2163 | nasal cavity disease | 22/282 | 1.15E-10 | 22 |
| DOID:2825 | nose disease | 22/282 | 1.15E-10 | 22 |
| DOID:4483 | rhinitis | 22/282 | 1.15E-10 | 22 |
| DOID:399 | tuberculosis | 27/282 | 1.22E-10 | 27 |
| DOID:16 | integumentary system disease | 44/282 | 1.38E-10 | 44 |
| DOID:37 | skin disease | 41/282 | 1.68E-10 | 41 |
| DOID:28 | endocrine system disease | 44/282 | 2.00E-10 | 44 |
| DOID:3388 | periodontal disease | 25/282 | 2.90E-10 | 25 |
| DOID:4450 | renal cell carcinoma | 40/282 | 3.02E-10 | 40 |
| DOID:635 | acquired immunodeficiency syndrome | 18/282 | 5.88E-10 | 18 |
| DOID:1883 | hepatitis C | 32/282 | 5.88E-10 | 32 |
| DOID:5082 | liver cirrhosis | 31/282 | 6.13E-10 | 31 |
| DOID:2349 | arteriosclerosis | 40/282 | 9.62E-10 | 40 |
| DOID:120 | female reproductive organ cancer | 46/282 | 1.09E-09 | 46 |
| DOID:974 | upper respiratory tract disease | 23/282 | 1.22E-09 | 23 |
| DOID:1936 | atherosclerosis | 39/282 | 1.23E-09 | 39 |
| DOID:2348 | arteriosclerotic cardiovascular disease | 39/282 | 1.31E-09 | 39 |
| DOID:1176 | bronchial disease | 25/282 | 1.97E-09 | 25 |
| DOID:3908 | non-small cell lung carcinoma | 44/282 | 1.97E-09 | 44 |
| DOID:824 | periodontitis | 22/282 | 2.66E-09 | 22 |
| DOID:1091 | tooth disease | 25/282 | 6.53E-09 | 25 |
| DOID:403 | mouth disease | 27/282 | 6.53E-09 | 27 |
| DOID:2213 | hemorrhagic disease | 24/282 | 8.93E-09 | 24 |
| DOID:3910 | lung adenocarcinoma | 26/282 | 9.30E-09 | 26 |
| DOID:2841 | asthma | 22/282 | 2.17E-08 | 22 |
| DOID:50 | thyroid gland disease | 24/282 | 2.17E-08 | 24 |
| DOID:10952 | nephritis | 23/282 | 2.32E-08 | 23 |
| DOID:3459 | breast carcinoma | 39/282 | 2.36E-08 | 39 |
| DOID:0060100 | musculoskeletal system cancer | 42/282 | 3.41E-08 | 42 |
| DOID:2394 | ovarian cancer | 34/282 | 5.03E-08 | 34 |
| DOID:2043 | hepatitis B | 26/282 | 7.57E-08 | 26 |
| DOID:5295 | intestinal disease | 23/282 | 7.93E-08 | 23 |
| DOID:201 | connective tissue cancer | 37/282 | 9.84E-08 | 37 |
| DOID:10534 | stomach cancer | 31/282 | 1.01E-07 | 31 |
| DOID:854 | collagen disease | 26/282 | 1.09E-07 | 26 |
| DOID:1037 | lymphoblastic leukemia | 41/282 | 1.28E-07 | 41 |
| DOID:5844 | myocardial infarction | 31/282 | 1.48E-07 | 31 |
| DOID:12365 | malaria | 18/282 | 2.13E-07 | 18 |
| DOID:5517 | stomach carcinoma | 22/282 | 2.20E-07 | 22 |
| DOID:9500 | leukocyte disease | 17/282 | 2.20E-07 | 17 |
| DOID:3326 | purpura | 15/282 | 2.32E-07 | 15 |
| DOID:4766 | embryoma | 35/282 | 2.65E-07 | 35 |
| DOID:688 | embryonal cancer | 36/282 | 3.50E-07 | 36 |
| DOID:0070004 | myeloma | 33/282 | 3.50E-07 | 33 |
| DOID:7998 | hyperthyroidism | 17/282 | 3.98E-07 | 17 |
| DOID:2994 | germ cell cancer | 38/282 | 3.98E-07 | 38 |
| DOID:8398 | osteoarthritis | 24/282 | 4.14E-07 | 24 |
| DOID:1575 | rheumatic disease | 24/282 | 4.42E-07 | 24 |
| DOID:418 | systemic scleroderma | 24/282 | 4.42E-07 | 24 |
| DOID:419 | scleroderma | 24/282 | 4.42E-07 | 24 |
| DOID:4138 | bile duct disease | 16/282 | 4.56E-07 | 16 |
| DOID:4960 | bone marrow cancer | 33/282 | 4.58E-07 | 33 |
| DOID:9538 | multiple myeloma | 29/282 | 4.66E-07 | 29 |
| DOID:2723 | dermatitis | 24/282 | 4.66E-07 | 24 |
| DOID:9741 | biliary tract disease | 16/282 | 5.14E-07 | 16 |
| DOID:3393 | coronary artery disease | 34/282 | 5.53E-07 | 34 |
| DOID:75 | lymphatic system disease | 17/282 | 7.45E-07 | 17 |
| DOID:3310 | atopic dermatitis | 21/282 | 7.45E-07 | 21 |
| DOID:12361 | Graves' disease | 15/282 | 9.97E-07 | 15 |
| DOID:9074 | systemic lupus erythematosus | 15/282 | 1.17E-06 | 15 |
| DOID:5409 | lung small cell carcinoma | 14/282 | 1.64E-06 | 14 |
| DOID:7166 | thyroiditis | 12/282 | 2.20E-06 | 12 |
| DOID:865 | vasculitis | 18/282 | 2.28E-06 | 18 |
| DOID:2151 | malignant ovarian surface epithelial-stromal neoplasm | 29/282 | 2.33E-06 | 29 |
| DOID:2152 | ovary epithelial cancer | 29/282 | 2.33E-06 | 29 |
| DOID:4001 | ovarian carcinoma | 29/282 | 2.33E-06 | 29 |
| DOID:0060005 | autoimmune disease of endocrine system | 15/282 | 2.86E-06 | 15 |
| DOID:8857 | lupus erythematosus | 15/282 | 2.86E-06 | 15 |
| DOID:5679 | retinal disease | 33/282 | 3.02E-06 | 33 |
| DOID:1074 | kidney failure | 20/282 | 4.63E-06 | 20 |
| DOID:9970 | obesity | 30/282 | 4.63E-06 | 30 |
| DOID:1588 | thrombocytopenia | 13/282 | 5.01E-06 | 13 |
| DOID:11054 | urinary bladder cancer | 16/282 | 8.13E-06 | 16 |
| DOID:4007 | bladder carcinoma | 12/282 | 8.13E-06 | 12 |
| DOID:654 | overnutrition | 30/282 | 8.15E-06 | 30 |
| DOID:2452 | thrombophilia | 8/282 | 8.65E-06 | 8 |
| DOID:552 | pneumonia | 16/282 | 8.92E-06 | 16 |
| DOID:229 | female reproductive system disease | 22/282 | 8.96E-06 | 22 |
| DOID:1107 | esophageal carcinoma | 17/282 | 1.09E-05 | 17 |
| DOID:184 | bone cancer | 25/282 | 1.10E-05 | 25 |
| DOID:11077 | brucellosis | 10/282 | 1.16E-05 | 10 |
| DOID:9415 | allergic asthma | 11/282 | 1.20E-05 | 11 |
| DOID:10591 | pre-eclampsia | 27/282 | 1.23E-05 | 27 |
| DOID:6000 | congestive heart failure | 24/282 | 1.25E-05 | 24 |
| DOID:3347 | osteosarcoma | 23/282 | 1.45E-05 | 23 |
| DOID:8469 | influenza | 16/282 | 1.52E-05 | 16 |
| DOID:219 | colon cancer | 27/282 | 1.57E-05 | 27 |
| DOID:9778 | irritable bowel syndrome | 13/282 | 1.63E-05 | 13 |
| DOID:10155 | intestinal cancer | 28/282 | 1.64E-05 | 28 |
| DOID:1793 | pancreatic cancer | 29/282 | 1.80E-05 | 29 |
| DOID:374 | nutrition disease | 30/282 | 1.94E-05 | 30 |
| DOID:438 | autoimmune disease of the nervous system | 11/282 | 1.98E-05 | 11 |
| DOID:326 | ischemia | 22/282 | 2.00E-05 | 22 |
| DOID:0050589 | inflammatory bowel disease | 14/282 | 2.03E-05 | 14 |
| DOID:9256 | colorectal cancer | 27/282 | 2.05E-05 | 27 |
| DOID:1040 | chronic lymphocytic leukemia | 23/282 | 2.12E-05 | 23 |
| DOID:5672 | large intestine cancer | 27/282 | 2.16E-05 | 27 |
| DOID:13406 | pulmonary sarcoidosis | 7/282 | 2.40E-05 | 7 |
| DOID:1115 | sarcoma | 21/282 | 2.81E-05 | 21 |
| DOID:12894 | Sjogren's syndrome | 12/282 | 3.06E-05 | 12 |
| DOID:12206 | dengue hemorrhagic fever | 6/282 | 3.08E-05 | 6 |
| DOID:1602 | lymphadenitis | 11/282 | 3.14E-05 | 11 |
| DOID:9942 | lymph node disease | 11/282 | 3.14E-05 | 11 |
| DOID:15 | reproductive system disease | 32/282 | 3.43E-05 | 32 |
| DOID:1205 | hypersensitivity reaction type I disease | 16/282 | 3.63E-05 | 16 |
| DOID:2218 | blood platelet disease | 14/282 | 4.03E-05 | 14 |
| DOID:5327 | retinal detachment | 5/282 | 4.03E-05 | 5 |
| DOID:10747 | lymphoid leukemia | 12/282 | 4.56E-05 | 12 |
| DOID:640 | encephalomyelitis | 7/282 | 4.57E-05 | 7 |
| DOID:627 | severe combined immunodeficiency | 10/282 | 4.95E-05 | 10 |
| DOID:0060122 | integumentary system cancer | 14/282 | 4.95E-05 | 14 |
| DOID:4159 | skin cancer | 14/282 | 4.95E-05 | 14 |
| DOID:4606 | bile duct cancer | 17/282 | 4.98E-05 | 17 |
| DOID:4897 | bile duct carcinoma | 17/282 | 4.98E-05 | 17 |
| DOID:0060049 | autoimmune disease of urogenital tract | 12/282 | 4.99E-05 | 12 |
| DOID:12236 | primary biliary cirrhosis | 12/282 | 4.99E-05 | 12 |
| DOID:26 | pancreas disease | 19/282 | 5.30E-05 | 19 |
| DOID:11394 | adult respiratory distress syndrome | 8/282 | 5.30E-05 | 8 |
| DOID:4914 | esophagus adenocarcinoma | 7/282 | 5.94E-05 | 7 |
| DOID:437 | myasthenia gravis | 9/282 | 5.97E-05 | 9 |
| DOID:628 | combined T cell and B cell immunodeficiency | 10/282 | 6.64E-05 | 10 |
| DOID:12205 | dengue disease | 6/282 | 6.64E-05 | 6 |
| DOID:14256 | adult-onset Still's disease | 5/282 | 6.95E-05 | 5 |
| DOID:9008 | psoriatic arthritis | 5/282 | 6.95E-05 | 5 |
| DOID:439 | neuromuscular junction disease | 9/282 | 7.12E-05 | 9 |
| DOID:9455 | lipid storage disease | 16/282 | 7.17E-05 | 16 |
| DOID:5041 | esophageal cancer | 17/282 | 7.32E-05 | 17 |
| DOID:6432 | pulmonary hypertension | 12/282 | 8.12E-05 | 12 |
| DOID:4607 | biliary tract cancer | 19/282 | 8.74E-05 | 19 |
| DOID:13378 | Kawasaki disease | 10/282 | 8.89E-05 | 10 |
| DOID:14069 | cerebral malaria | 7/282 | 0.000101 | 7 |
| DOID:2921 | glomerulonephritis | 12/282 | 0.000103 | 12 |
| DOID:9408 | acute myocardial infarction | 13/282 | 0.000103 | 13 |
| DOID:1781 | thyroid cancer | 21/282 | 0.000103 | 21 |
| DOID:9352 | type 2 diabetes mellitus | 21/282 | 0.000118 | 21 |
| DOID:4045 | muscle cancer | 14/282 | 0.000118 | 14 |
| DOID:3963 | thyroid carcinoma | 20/282 | 0.00013 | 20 |
| DOID:200 | giant cell tumor | 6/282 | 0.000131 | 6 |
| DOID:1520 | colon carcinoma | 18/282 | 0.000131 | 18 |
| DOID:1485 | cystic fibrosis | 16/282 | 0.00014 | 16 |
| DOID:4195 | hyperglycemia | 16/282 | 0.000153 | 16 |
| DOID:2462 | retinal vascular disease | 10/282 | 0.000158 | 10 |
| DOID:8947 | diabetic retinopathy | 10/282 | 0.000158 | 10 |
| DOID:8692 | myeloid leukemia | 16/282 | 0.000196 | 16 |
| DOID:9446 | cholangitis | 7/282 | 0.000209 | 7 |
| DOID:4896 | bile duct adenocarcinoma | 15/282 | 0.000213 | 15 |
| DOID:4947 | cholangiocarcinoma | 15/282 | 0.000213 | 15 |
| DOID:13241 | Behcet's disease | 12/282 | 0.000225 | 12 |
| DOID:8466 | retinal degeneration | 23/282 | 0.000229 | 23 |
| DOID:4074 | pancreas adenocarcinoma | 17/282 | 0.00025 | 17 |
| DOID:10887 | lepromatous leprosy | 4/282 | 0.00025 | 4 |
| DOID:12177 | common variable immunodeficiency | 7/282 | 0.000257 | 7 |
| DOID:2473 | opportunistic mycosis | 8/282 | 0.00026 | 8 |
| DOID:4989 | pancreatitis | 14/282 | 0.000293 | 14 |
| DOID:715 | T-cell leukemia | 10/282 | 0.000309 | 10 |
| DOID:3702 | cervical adenocarcinoma | 6/282 | 0.000315 | 6 |
| DOID:1564 | fungal infectious disease | 11/282 | 0.000317 | 11 |
| DOID:2893 | cervix carcinoma | 13/282 | 0.000334 | 13 |
| DOID:3211 | lysosomal storage disease | 16/282 | 0.000352 | 16 |
| DOID:4362 | cervical cancer | 13/282 | 0.000365 | 13 |
| DOID:2583 | agammaglobulinemia | 7/282 | 0.000385 | 7 |
| DOID:620 | blood protein disease | 7/282 | 0.000385 | 7 |
| DOID:4029 | gastritis | 10/282 | 0.00039 | 10 |
| DOID:0050136 | systemic mycosis | 8/282 | 0.000426 | 8 |
| DOID:1319 | brain cancer | 8/282 | 0.000426 | 8 |
| DOID:3247 | rhabdomyosarcoma | 11/282 | 0.000434 | 11 |
| DOID:655 | inherited metabolic disorder | 26/282 | 0.000466 | 26 |
| DOID:3717 | gastric adenocarcinoma | 12/282 | 0.000491 | 12 |
| DOID:1036 | chronic leukemia | 20/282 | 0.000509 | 20 |
| DOID:3620 | central nervous system cancer | 14/282 | 0.000509 | 14 |
| DOID:1686 | glaucoma | 13/282 | 0.000509 | 13 |
| DOID:9452 | fatty liver disease | 12/282 | 0.000533 | 12 |
| DOID:3070 | malignant glioma | 19/282 | 0.00055 | 19 |
| DOID:2115 | B cell deficiency | 7/282 | 0.000554 | 7 |
| DOID:350 | mastocytosis | 8/282 | 0.000572 | 8 |
| DOID:0060084 | cell type benign neoplasm | 33/282 | 0.000574 | 33 |
| DOID:2871 | endometrial carcinoma | 11/282 | 0.000579 | 11 |
| DOID:0060085 | organ system benign neoplasm | 22/282 | 0.000591 | 22 |
| DOID:1380 | endometrial cancer | 12/282 | 0.000631 | 12 |
| DOID:4043 | skeletal muscle cancer | 11/282 | 0.000638 | 11 |
| DOID:4251 | conjunctival disease | 7/282 | 0.000654 | 7 |
| DOID:363 | uterine cancer | 12/282 | 0.000687 | 12 |
| DOID:870 | neuropathy | 18/282 | 0.000689 | 18 |
| DOID:13042 | persistent fetal circulation syndrome | 4/282 | 0.000718 | 4 |
| DOID:3451 | skin carcinoma | 9/282 | 0.000743 | 9 |
| DOID:13207 | proliferative diabetic retinopathy | 7/282 | 0.000774 | 7 |
| DOID:10871 | age related macular degeneration | 10/282 | 0.00083 | 10 |
| DOID:2007 | degeneration of macula and posterior pole | 10/282 | 0.00083 | 10 |
| DOID:615 | leukopenia | 8/282 | 0.000866 | 8 |
| DOID:1542 | head and neck carcinoma | 20/282 | 0.000944 | 20 |
| DOID:0002116 | pterygium | 6/282 | 0.000954 | 6 |
| DOID:10139 | conjunctival degeneration | 6/282 | 0.000954 | 6 |
| DOID:10526 | conjunctival pterygium | 6/282 | 0.000954 | 6 |
| DOID:3192 | neurilemmoma | 6/282 | 0.000954 | 6 |
| DOID:0060058 | lymphoma | 11/282 | 0.001007 | 11 |
| DOID:4448 | macular degeneration | 10/282 | 0.001007 | 10 |
| DOID:4905 | pancreatic carcinoma | 20/282 | 0.001023 | 20 |
| DOID:11934 | head and neck cancer | 20/282 | 0.001139 | 20 |
| DOID:2513 | basal cell carcinoma | 6/282 | 0.001164 | 6 |
| DOID:13580 | cholestasis | 9/282 | 0.001168 | 9 |
| DOID:349 | systemic mastocytosis | 7/282 | 0.00124 | 7 |
| DOID:8778 | Crohn's disease | 8/282 | 0.001273 | 8 |
| DOID:76 | stomach disease | 10/282 | 0.001359 | 10 |
| DOID:10286 | prostate carcinoma | 13/282 | 0.001362 | 13 |
| DOID:2001 | neuroma | 6/282 | 0.001401 | 6 |
| DOID:4948 | gallbladder carcinoma | 8/282 | 0.001439 | 8 |
| DOID:2957 | pulmonary tuberculosis | 5/282 | 0.001582 | 5 |
| DOID:1586 | rheumatic fever | 4/282 | 0.001582 | 4 |
| DOID:2089 | constipation | 4/282 | 0.001582 | 4 |
| DOID:9779 | bowel dysfunction | 4/282 | 0.001582 | 4 |
| DOID:14512 | candidal paronychia | 3/282 | 0.001582 | 3 |
| DOID:3121 | gallbladder cancer | 8/282 | 0.001608 | 8 |
| DOID:127 | leiomyoma | 11/282 | 0.00183 | 11 |
| DOID:1024 | leprosy | 7/282 | 0.001909 | 7 |
| DOID:381 | arthropathy | 11/282 | 0.002172 | 11 |
| DOID:12120 | pulmonary alveolar proteinosis | 4/282 | 0.002232 | 4 |
| DOID:4305 | bone giant cell tumor | 4/282 | 0.002232 | 4 |
| DOID:614 | lymphopenia | 4/282 | 0.002232 | 4 |
| DOID:11162 | respiratory failure | 8/282 | 0.002307 | 8 |
| DOID:440 | neuromuscular disease | 10/282 | 0.002329 | 10 |
| DOID:0060180 | colitis | 9/282 | 0.002397 | 9 |
| DOID:8577 | ulcerative colitis | 9/282 | 0.002397 | 9 |
| DOID:784 | chronic kidney failure | 9/282 | 0.002657 | 9 |
| DOID:2942 | bronchiolitis | 3/282 | 0.00292 | 3 |
| DOID:11123 | Henoch-Schoenlein purpura | 5/282 | 0.003038 | 5 |
| DOID:1557 | hypersensitivity reaction type III disease | 5/282 | 0.003038 | 5 |
| DOID:9809 | hypersensitivity vasculitis | 5/282 | 0.003038 | 5 |
| DOID:0060115 | nervous system benign neoplasm | 6/282 | 0.003231 | 6 |
| DOID:289 | endometriosis | 10/282 | 0.003572 | 10 |
| DOID:11247 | disseminated intravascular coagulation | 4/282 | 0.004054 | 4 |
| DOID:12689 | acoustic neuroma | 4/282 | 0.004054 | 4 |
| DOID:5520 | head and neck squamous cell carcinoma | 15/282 | 0.004207 | 15 |
| DOID:1067 | open-angle glaucoma | 8/282 | 0.004398 | 8 |
| DOID:9119 | acute myeloid leukemia | 11/282 | 0.004596 | 11 |
| DOID:11168 | anogenital venereal wart | 3/282 | 0.004696 | 3 |
| DOID:13138 | acute proliferative glomerulonephritis | 3/282 | 0.004696 | 3 |
| DOID:13139 | crescentic glomerulonephritis | 3/282 | 0.004696 | 3 |
| DOID:4830 | adenosquamous carcinoma | 3/282 | 0.004696 | 3 |
| DOID:869 | cholesteatoma | 3/282 | 0.004696 | 3 |
| DOID:3969 | papillary thyroid carcinoma | 11/282 | 0.004849 | 11 |
| DOID:9952 | acute lymphocytic leukemia | 7/282 | 0.005156 | 7 |
| DOID:2621 | autonomic nervous system neoplasm | 25/282 | 0.005246 | 25 |
| DOID:769 | neuroblastoma | 25/282 | 0.005246 | 25 |
| DOID:1192 | peripheral nervous system neoplasm | 26/282 | 0.005249 | 26 |
| DOID:3405 | histiocytosis | 6/282 | 0.005649 | 6 |
| DOID:3376 | bone osteosarcoma | 7/282 | 0.005711 | 7 |
| DOID:9471 | meningitis | 5/282 | 0.006243 | 5 |
| DOID:986 | alopecia areata | 5/282 | 0.006243 | 5 |
| DOID:866 | vein disease | 6/282 | 0.006396 | 6 |
| DOID:883 | parasitic helminthiasis infectious disease | 6/282 | 0.006396 | 6 |
| DOID:3319 | lymphangioleiomyomatosis | 4/282 | 0.006468 | 4 |
| DOID:633 | myositis | 9/282 | 0.006815 | 9 |
| DOID:5683 | hereditary breast ovarian cancer | 17/282 | 0.006895 | 17 |
| DOID:1618 | breast fibroadenoma | 3/282 | 0.006927 | 3 |
| DOID:3498 | pancreatic ductal adenocarcinoma | 10/282 | 0.00739 | 10 |
| DOID:12306 | vitiligo | 8/282 | 0.007458 | 8 |
| DOID:2355 | anemia | 18/282 | 0.007669 | 18 |
| DOID:10652 | Alzheimer's disease | 27/282 | 0.007738 | 27 |
| DOID:0050736 | autosomal dominant disease | 26/282 | 0.007818 | 26 |
| DOID:0050938 | breast lobular carcinoma | 4/282 | 0.007857 | 4 |
| DOID:11971 | synostosis | 4/282 | 0.007857 | 4 |
| DOID:171 | neuroectodermal tumor | 4/282 | 0.007857 | 4 |
| DOID:3457 | invasive lobular carcinoma | 4/282 | 0.007857 | 4 |
| DOID:9675 | pulmonary emphysema | 4/282 | 0.007857 | 4 |
| DOID:576 | proteinuria | 8/282 | 0.007947 | 8 |
| DOID:936 | brain disease | 28/282 | 0.008132 | 28 |
| DOID:13141 | uveitis | 5/282 | 0.008235 | 5 |
| DOID:680 | tauopathy | 27/282 | 0.008514 | 27 |
| DOID:0060060 | non-Hodgkin lymphoma | 6/282 | 0.008893 | 6 |
| DOID:1790 | malignant mesothelioma | 6/282 | 0.008893 | 6 |
| DOID:1184 | nephrotic syndrome | 7/282 | 0.009119 | 7 |
| DOID:9588 | encephalitis | 7/282 | 0.009119 | 7 |
| DOID:999 | eosinophilia | 3/282 | 0.009404 | 3 |
| DOID:3262 | phagocyte bactericidal dysfunction | 4/282 | 0.009404 | 4 |
| DOID:3479 | uveal cancer | 4/282 | 0.009404 | 4 |
| DOID:4248 | coronary stenosis | 4/282 | 0.009404 | 4 |
| DOID:6039 | uveal melanoma | 4/282 | 0.009404 | 4 |
| DOID:2527 | nephrosis | 7/282 | 0.010935 | 7 |
| DOID:2645 | benign mesothelioma | 7/282 | 0.010935 | 7 |
| DOID:10825 | essential hypertension | 11/282 | 0.011112 | 11 |
| DOID:299 | adenocarcinoma | 13/282 | 0.011123 | 13 |
| DOID:2876 | laryngeal squamous cell carcinoma | 4/282 | 0.011397 | 4 |
| DOID:3744 | cervical squamous cell carcinoma | 8/282 | 0.011662 | 8 |
| DOID:6364 | migraine | 8/282 | 0.011662 | 8 |
| DOID:8850 | salivary gland cancer | 7/282 | 0.011838 | 7 |
| DOID:1555 | urticaria | 6/282 | 0.012128 | 6 |
| DOID:5100 | middle ear disease | 5/282 | 0.012134 | 5 |
| DOID:657 | adenoma | 22/282 | 0.012134 | 22 |
| DOID:0050598 | extrapulmonary tuberculosis | 3/282 | 0.012134 | 3 |
| DOID:106 | pleural tuberculosis | 3/282 | 0.012134 | 3 |
| DOID:1080 | filariasis | 3/282 | 0.012134 | 3 |
| DOID:2340 | craniosynostosis | 3/282 | 0.012134 | 3 |
| DOID:3179 | inverted papilloma | 3/282 | 0.012134 | 3 |
| DOID:3314 | angiomyolipoma | 3/282 | 0.012134 | 3 |
| DOID:4233 | clear cell sarcoma | 3/282 | 0.012134 | 3 |
| DOID:4449 | macular retinal edema | 3/282 | 0.012134 | 3 |
| DOID:5200 | urinary tract obstruction | 3/282 | 0.012134 | 3 |
| DOID:6929 | retinal edema | 3/282 | 0.012134 | 3 |
| DOID:8929 | atrophic gastritis | 3/282 | 0.012134 | 3 |
| DOID:3458 | breast adenocarcinoma | 6/282 | 0.012895 | 6 |
| DOID:1532 | pleural disease | 4/282 | 0.012895 | 4 |
| DOID:3302 | chordoma | 4/282 | 0.012895 | 4 |
| DOID:3303 | notochordal cancer | 4/282 | 0.012895 | 4 |
| DOID:452 | pleomorphic adenoma | 4/282 | 0.012895 | 4 |
| DOID:799 | varicose veins | 4/282 | 0.012895 | 4 |
| DOID:13375 | temporal arteritis | 5/282 | 0.013237 | 5 |
| DOID:525 | central nervous system vasculitis | 5/282 | 0.013237 | 5 |
| DOID:8618 | oral cavity cancer | 7/282 | 0.014626 | 7 |
| DOID:1483 | gingival disease | 5/282 | 0.015026 | 5 |
| DOID:3355 | fibrosarcoma | 5/282 | 0.015026 | 5 |
| DOID:10113 | trypanosomiasis | 4/282 | 0.015219 | 4 |
| DOID:11612 | polycystic ovary syndrome | 13/282 | 0.015341 | 13 |
| DOID:2566 | corneal dystrophy | 3/282 | 0.015582 | 3 |
| DOID:10124 | corneal disease | 7/282 | 0.015722 | 7 |
| DOID:12603 | acute leukemia | 10/282 | 0.016003 | 10 |
| DOID:13250 | diarrhea | 5/282 | 0.016771 | 5 |
| DOID:1927 | sphingolipidosis | 5/282 | 0.016771 | 5 |
| DOID:1307 | dementia | 12/282 | 0.017419 | 12 |
| DOID:1100 | ovarian disease | 7/282 | 0.018442 | 7 |
| DOID:4971 | myelofibrosis | 6/282 | 0.018715 | 6 |
| DOID:12716 | newborn respiratory distress syndrome | 5/282 | 0.018742 | 5 |
| DOID:1923 | sex differentiation disease | 5/282 | 0.018742 | 5 |
| DOID:0060082 | breast benign neoplasm | 3/282 | 0.019382 | 3 |
| DOID:0060097 | thoracic benign neoplasm | 3/282 | 0.019382 | 3 |
| DOID:12337 | varicocele | 3/282 | 0.019382 | 3 |
| DOID:14504 | Niemann-Pick disease | 3/282 | 0.019382 | 3 |
| DOID:3500 | gallbladder adenocarcinoma | 3/282 | 0.019382 | 3 |
| DOID:9742 | pelvic varices | 3/282 | 0.019382 | 3 |
| DOID:0050624 | gastrointestinal system benign neoplasm | 6/282 | 0.020132 | 6 |
| DOID:2048 | autoimmune hepatitis | 4/282 | 0.02017 | 4 |
| DOID:4928 | intrahepatic cholangiocarcinoma | 5/282 | 0.020504 | 5 |
| DOID:5158 | pleural cancer | 5/282 | 0.020504 | 5 |
| DOID:7474 | malignant pleural mesothelioma | 5/282 | 0.020504 | 5 |
| DOID:2742 | auditory system disease | 9/282 | 0.021508 | 9 |
| DOID:4079 | heart valve disease | 6/282 | 0.021791 | 6 |
| DOID:987 | alopecia | 6/282 | 0.023809 | 6 |
| DOID:13608 | biliary atresia | 3/282 | 0.023809 | 3 |
| DOID:6132 | bronchitis | 3/282 | 0.023809 | 3 |
| DOID:841 | extrinsic allergic alveolitis | 3/282 | 0.023809 | 3 |
| DOID:783 | end stage renal failure | 5/282 | 0.025294 | 5 |
| DOID:1934 | dysostosis | 4/282 | 0.026452 | 4 |
| DOID:2277 | gonadal disease | 7/282 | 0.027848 | 7 |
| DOID:3068 | glioblastoma multiforme | 7/282 | 0.027848 | 7 |
| DOID:10964 | cholesteatoma of middle ear | 2/282 | 0.028302 | 2 |
| DOID:11433 | middle ear cholesteatoma | 2/282 | 0.028302 | 2 |
| DOID:4706 | infratentorial cancer | 2/282 | 0.028302 | 2 |
| DOID:121 | vaginal disease | 3/282 | 0.028584 | 3 |
| DOID:2170 | vaginitis | 3/282 | 0.028584 | 3 |
| DOID:2945 | severe acute respiratory syndrome | 3/282 | 0.028584 | 3 |
| DOID:3385 | bacterial vaginosis | 3/282 | 0.028584 | 3 |
| DOID:4330 | non-langerhans-cell histiocytosis | 3/282 | 0.028584 | 3 |
| DOID:10588 | adrenoleukodystrophy | 4/282 | 0.029452 | 4 |
| DOID:2600 | laryngeal carcinoma | 4/282 | 0.029452 | 4 |
| DOID:0060119 | pharynx cancer | 5/282 | 0.030171 | 5 |
| DOID:3962 | follicular thyroid carcinoma | 6/282 | 0.03218 | 6 |
| DOID:5157 | benign pleural mesothelioma | 5/282 | 0.033225 | 5 |
| DOID:12176 | goiter | 3/282 | 0.034091 | 3 |
| DOID:14268 | sclerosing cholangitis | 3/282 | 0.034091 | 3 |
| DOID:2870 | endometrial adenocarcinoma | 3/282 | 0.034091 | 3 |
| DOID:0060031 | autoimmune disease of gastrointestinal tract | 9/282 | 0.03423 | 9 |
| DOID:0050904 | salivary gland carcinoma | 6/282 | 0.034395 | 6 |
| DOID:3146 | lipid metabolism disorder | 8/282 | 0.036451 | 8 |
| DOID:11201 | parathyroid gland disease | 4/282 | 0.037201 | 4 |
| DOID:0050700 | cardiomyopathy | 11/282 | 0.037313 | 11 |
| DOID:12930 | dilated cardiomyopathy | 8/282 | 0.03841 | 8 |
| DOID:0050567 | orofacial cleft | 5/282 | 0.038666 | 5 |
| DOID:674 | cleft palate | 5/282 | 0.038666 | 5 |
| DOID:12169 | carpal tunnel syndrome | 2/282 | 0.038666 | 2 |
| DOID:12309 | urticaria pigmentosa | 2/282 | 0.038666 | 2 |
| DOID:14735 | hereditary angioedema | 2/282 | 0.038666 | 2 |
| DOID:4682 | extrahepatic bile duct carcinoma | 2/282 | 0.038666 | 2 |
| DOID:573 | nerve compression syndrome | 2/282 | 0.038666 | 2 |
| DOID:5870 | eosinophilic pneumonia | 2/282 | 0.038666 | 2 |
| DOID:3069 | astrocytoma | 9/282 | 0.038674 | 9 |
| DOID:0080000 | muscular disease | 23/282 | 0.038674 | 23 |
| DOID:4535 | hypotrichosis | 6/282 | 0.038674 | 6 |
| DOID:10808 | gastric ulcer | 3/282 | 0.038674 | 3 |
| DOID:11729 | Lyme disease | 3/282 | 0.038674 | 3 |
| DOID:2615 | papilloma | 3/282 | 0.038674 | 3 |
| DOID:321 | tropical spastic paraparesis | 3/282 | 0.038674 | 3 |
| DOID:3265 | chronic granulomatous disease | 3/282 | 0.038674 | 3 |
| DOID:1389 | polyneuropathy | 4/282 | 0.039935 | 4 |
| DOID:4798 | aggressive systemic mastocytosis | 4/282 | 0.039935 | 4 |
| DOID:10603 | glucose intolerance | 5/282 | 0.041204 | 5 |
| DOID:341 | peripheral vascular disease | 6/282 | 0.044128 | 6 |
| DOID:5353 | colonic disease | 4/282 | 0.044451 | 4 |
| DOID:0060033 | autoimmune disease of peripheral nervous system | 3/282 | 0.044451 | 3 |
| DOID:10762 | portal hypertension | 3/282 | 0.044451 | 3 |
| DOID:10976 | membranous glomerulonephritis | 3/282 | 0.044451 | 3 |
| DOID:1273 | respiratory syncytial virus infectious disease | 3/282 | 0.044451 | 3 |
| DOID:12842 | Guillain-Barre syndrome | 3/282 | 0.044451 | 3 |
| DOID:13564 | aspergillosis | 3/282 | 0.044451 | 3 |
| DOID:1508 | candidiasis | 3/282 | 0.044451 | 3 |
| DOID:0060036 | intrinsic cardiomyopathy | 10/282 | 0.044801 | 10 |
| DOID:161 | keratosis | 5/282 | 0.047751 | 5 |
| DOID:3480 | uveal disease | 5/282 | 0.047751 | 5 |
| DOID:9744 | type 1 diabetes mellitus | 4/282 | 0.048434 | 4 |
| DOID:3748 | esophagus squamous cell carcinoma | 6/282 | 0.048958 | 6 |
| DOID:11030 | corneal edema | 2/282 | 0.048958 | 2 |
| DOID:11031 | bullous keratopathy | 2/282 | 0.048958 | 2 |
| DOID:11193 | syndactyly | 2/282 | 0.048958 | 2 |
| DOID:1727 | retinal vein occlusion | 2/282 | 0.048958 | 2 |
| DOID:3284 | thymic carcinoma | 2/282 | 0.048958 | 2 |
| DOID:4331 | burning mouth syndrome | 2/282 | 0.048958 | 2 |
| DOID:5425 | ovarian hyperstimulation syndrome | 2/282 | 0.048958 | 2 |
| DOID:423 | myopathy | 22/282 | 0.04984 | 22 |
| DOID:66 | muscle tissue disease | 22/282 | 0.04984 | 22 |
| DOID:0050743 | mature T-cell and NK-cell lymphoma | 3/282 | 0.04984 | 3 |
| DOID:0050749 | peripheral T-cell lymphoma | 3/282 | 0.04984 | 3 |
| DOID:0060061 | cutaneous T cell lymphoma | 3/282 | 0.04984 | 3 |
| DOID:8719 | in situ carcinoma | 3/282 | 0.04984 | 3 |
