# Supplementary material for: Identification of an immune-related risk signature for predicting prognosis in clear cell renal cell carcinoma
Source: Aging (Albany NY). 2020 Feb 6;12(3):2302–32. doi: 10.18632/aging.102746 (PMC7041771; doi:10.18632/aging.102746)
Supplement: Supplementary Table 7 [file aging-12-102746-s003..docx]

Supplementary Table 7. Disease ontology analysis based on 47 immune-related hub genes.

| ID | Description | GeneRatio | p.adjust | Count |
| --- | --- | --- | --- | --- |
| DOID:850 | lung disease | 27/45 | 5.26E-19 | 27 |
| DOID:3083 | chronic obstructive pulmonary disease | 17/45 | 2.65E-13 | 17 |
| DOID:2320 | obstructive lung disease | 18/45 | 2.81E-12 | 18 |
| DOID:104 | bacterial infectious disease | 17/45 | 4.35E-12 | 17 |
| DOID:612 | primary immunodeficiency disease | 14/45 | 1.81E-10 | 14 |
| DOID:2237 | hepatitis | 18/45 | 4.57E-10 | 18 |
| DOID:0050338 | primary bacterial infectious disease | 14/45 | 1.83E-09 | 14 |
| DOID:526 | Human immunodeficiency virus infectious disease | 12/45 | 8.28E-09 | 12 |
| DOID:1883 | hepatitis C | 13/45 | 1.65E-08 | 13 |
| DOID:557 | kidney disease | 16/45 | 8.56E-08 | 16 |
| DOID:3082 | interstitial lung disease | 11/45 | 8.56E-08 | 11 |
| DOID:5082 | liver cirrhosis | 12/45 | 8.96E-08 | 12 |
| DOID:18 | urinary system disease | 16/45 | 1.17E-07 | 16 |
| DOID:1936 | atherosclerosis | 14/45 | 1.17E-07 | 14 |
| DOID:2348 | arteriosclerotic cardiovascular disease | 14/45 | 1.17E-07 | 14 |
| DOID:3393 | coronary artery disease | 14/45 | 1.23E-07 | 14 |
| DOID:635 | acquired immunodeficiency syndrome | 8/45 | 1.46E-07 | 8 |
| DOID:2349 | arteriosclerosis | 14/45 | 1.46E-07 | 14 |
| DOID:7166 | thyroiditis | 7/45 | 4.41E-07 | 7 |
| DOID:11335 | sarcoidosis | 8/45 | 4.98E-07 | 8 |
| DOID:2916 | hypersensitivity reaction type IV disease | 8/45 | 6.88E-07 | 8 |
| DOID:5844 | myocardial infarction | 12/45 | 6.89E-07 | 12 |
| DOID:2789 | parasitic protozoa infectious disease | 9/45 | 6.89E-07 | 9 |
| DOID:1247 | blood coagulation disease | 10/45 | 1.05E-06 | 10 |
| DOID:5679 | retinal disease | 13/45 | 1.11E-06 | 13 |
| DOID:3388 | periodontal disease | 9/45 | 1.34E-06 | 9 |
| DOID:4481 | allergic rhinitis | 8/45 | 1.36E-06 | 8 |
| DOID:2163 | nasal cavity disease | 8/45 | 1.44E-06 | 8 |
| DOID:2825 | nose disease | 8/45 | 1.44E-06 | 8 |
| DOID:4483 | rhinitis | 8/45 | 1.44E-06 | 8 |
| DOID:12365 | malaria | 8/45 | 1.62E-06 | 8 |
| DOID:5327 | retinal detachment | 4/45 | 2.04E-06 | 4 |
| DOID:1398 | parasitic infectious disease | 9/45 | 2.05E-06 | 9 |
| DOID:2213 | hemorrhagic disease | 9/45 | 2.23E-06 | 9 |
| DOID:399 | tuberculosis | 9/45 | 2.71E-06 | 9 |
| DOID:5295 | intestinal disease | 9/45 | 2.78E-06 | 9 |
| DOID:50 | thyroid gland disease | 9/45 | 3.01E-06 | 9 |
| DOID:1091 | tooth disease | 9/45 | 3.44E-06 | 9 |
| DOID:12361 | Graves' disease | 7/45 | 3.96E-06 | 7 |
| DOID:865 | vasculitis | 8/45 | 4.65E-06 | 8 |
| DOID:37 | skin disease | 12/45 | 4.65E-06 | 12 |
| DOID:3770 | pulmonary fibrosis | 8/45 | 4.77E-06 | 8 |
| DOID:974 | upper respiratory tract disease | 8/45 | 5.48E-06 | 8 |
| DOID:9446 | cholangitis | 5/45 | 5.48E-06 | 5 |
| DOID:3213 | demyelinating disease | 9/45 | 5.53E-06 | 9 |
| DOID:4138 | bile duct disease | 7/45 | 5.53E-06 | 7 |
| DOID:0060005 | autoimmune disease of endocrine system | 7/45 | 5.74E-06 | 7 |
| DOID:9741 | biliary tract disease | 7/45 | 5.74E-06 | 7 |
| DOID:0050589 | inflammatory bowel disease | 7/45 | 7.10E-06 | 7 |
| DOID:2462 | retinal vascular disease | 6/45 | 8.62E-06 | 6 |
| DOID:8947 | diabetic retinopathy | 6/45 | 8.62E-06 | 6 |
| DOID:403 | mouth disease | 9/45 | 8.92E-06 | 9 |
| DOID:7998 | hyperthyroidism | 7/45 | 1.01E-05 | 7 |
| DOID:15 | reproductive system disease | 12/45 | 1.01E-05 | 12 |
| DOID:229 | female reproductive system disease | 9/45 | 1.05E-05 | 9 |
| DOID:16 | integumentary system disease | 12/45 | 1.21E-05 | 12 |
| DOID:0070004 | myeloma | 11/45 | 1.22E-05 | 11 |
| DOID:74 | hematopoietic system disease | 13/45 | 1.24E-05 | 13 |
| DOID:13207 | proliferative diabetic retinopathy | 5/45 | 1.31E-05 | 5 |
| DOID:4960 | bone marrow cancer | 11/45 | 1.35E-05 | 11 |
| DOID:9538 | multiple myeloma | 10/45 | 1.46E-05 | 10 |
| DOID:1176 | bronchial disease | 8/45 | 1.71E-05 | 8 |
| DOID:1074 | kidney failure | 8/45 | 1.95E-05 | 8 |
| DOID:10534 | stomach cancer | 10/45 | 1.96E-05 | 10 |
| DOID:3996 | urinary system cancer | 13/45 | 2.01E-05 | 13 |
| DOID:9352 | type 2 diabetes mellitus | 9/45 | 2.16E-05 | 9 |
| DOID:10871 | age related macular degeneration | 6/45 | 2.35E-05 | 6 |
| DOID:2007 | degeneration of macula and posterior pole | 6/45 | 2.35E-05 | 6 |
| DOID:4448 | macular degeneration | 6/45 | 2.73E-05 | 6 |
| DOID:263 | kidney cancer | 12/45 | 2.88E-05 | 12 |
| DOID:2377 | multiple sclerosis | 8/45 | 3.16E-05 | 8 |
| DOID:13406 | pulmonary sarcoidosis | 4/45 | 3.32E-05 | 4 |
| DOID:824 | periodontitis | 7/45 | 3.35E-05 | 7 |
| DOID:3702 | cervical adenocarcinoma | 4/45 | 3.98E-05 | 4 |
| DOID:3620 | central nervous system cancer | 7/45 | 4.06E-05 | 7 |
| DOID:4905 | pancreatic carcinoma | 9/45 | 4.18E-05 | 9 |
| DOID:4451 | renal carcinoma | 11/45 | 4.38E-05 | 11 |
| DOID:11168 | anogenital venereal wart | 3/45 | 4.38E-05 | 3 |
| DOID:869 | cholesteatoma | 3/45 | 4.38E-05 | 3 |
| DOID:640 | encephalomyelitis | 4/45 | 4.48E-05 | 4 |
| DOID:3459 | breast carcinoma | 11/45 | 4.53E-05 | 11 |
| DOID:8778 | Crohn's disease | 5/45 | 5.68E-05 | 5 |
| DOID:8398 | osteoarthritis | 8/45 | 5.68E-05 | 8 |
| DOID:1793 | pancreatic cancer | 10/45 | 5.68E-05 | 10 |
| DOID:1575 | rheumatic disease | 8/45 | 5.68E-05 | 8 |
| DOID:418 | systemic scleroderma | 8/45 | 5.68E-05 | 8 |
| DOID:419 | scleroderma | 8/45 | 5.68E-05 | 8 |
| DOID:2841 | asthma | 7/45 | 5.74E-05 | 7 |
| DOID:2723 | dermatitis | 8/45 | 5.74E-05 | 8 |
| DOID:2452 | thrombophilia | 4/45 | 5.74E-05 | 4 |
| DOID:4079 | heart valve disease | 5/45 | 5.76E-05 | 5 |
| DOID:28 | endocrine system disease | 11/45 | 5.90E-05 | 11 |
| DOID:870 | neuropathy | 8/45 | 6.69E-05 | 8 |
| DOID:9471 | meningitis | 4/45 | 7.67E-05 | 4 |
| DOID:854 | collagen disease | 8/45 | 8.49E-05 | 8 |
| DOID:326 | ischemia | 8/45 | 9.36E-05 | 8 |
| DOID:10952 | nephritis | 7/45 | 9.63E-05 | 7 |
| DOID:10591 | pre-eclampsia | 9/45 | 0.000116 | 9 |
| DOID:3310 | atopic dermatitis | 7/45 | 0.000116 | 7 |
| DOID:1080 | filariasis | 3/45 | 0.000116 | 3 |
| DOID:3314 | angiomyolipoma | 3/45 | 0.000116 | 3 |
| DOID:5517 | stomach carcinoma | 7/45 | 0.000119 | 7 |
| DOID:11394 | adult respiratory distress syndrome | 4/45 | 0.000144 | 4 |
| DOID:4074 | pancreas adenocarcinoma | 7/45 | 0.00016 | 7 |
| DOID:13375 | temporal arteritis | 4/45 | 0.00016 | 4 |
| DOID:525 | central nervous system vasculitis | 4/45 | 0.00016 | 4 |
| DOID:4989 | pancreatitis | 6/45 | 0.000247 | 6 |
| DOID:3326 | purpura | 5/45 | 0.000247 | 5 |
| DOID:5409 | lung small cell carcinoma | 5/45 | 0.000262 | 5 |
| DOID:6364 | migraine | 5/45 | 0.000278 | 5 |
| DOID:3910 | lung adenocarcinoma | 7/45 | 0.000289 | 7 |
| DOID:883 | parasitic helminthiasis infectious disease | 4/45 | 0.000302 | 4 |
| DOID:12206 | dengue hemorrhagic fever | 3/45 | 0.00031 | 3 |
| DOID:9778 | irritable bowel syndrome | 5/45 | 0.000326 | 5 |
| DOID:14268 | sclerosing cholangitis | 3/45 | 0.000376 | 3 |
| DOID:4896 | bile duct adenocarcinoma | 6/45 | 0.000376 | 6 |
| DOID:4947 | cholangiocarcinoma | 6/45 | 0.000376 | 6 |
| DOID:3247 | rhabdomyosarcoma | 5/45 | 0.000379 | 5 |
| DOID:437 | myasthenia gravis | 4/45 | 0.000386 | 4 |
| DOID:9074 | systemic lupus erythematosus | 5/45 | 0.000414 | 5 |
| DOID:11077 | brucellosis | 4/45 | 0.000414 | 4 |
| DOID:439 | neuromuscular junction disease | 4/45 | 0.000414 | 4 |
| DOID:8466 | retinal degeneration | 8/45 | 0.000426 | 8 |
| DOID:10808 | gastric ulcer | 3/45 | 0.000426 | 3 |
| DOID:12205 | dengue disease | 3/45 | 0.000426 | 3 |
| DOID:1115 | sarcoma | 7/45 | 0.000426 | 7 |
| DOID:4043 | skeletal muscle cancer | 5/45 | 0.000443 | 5 |
| DOID:440 | neuromuscular disease | 5/45 | 0.000443 | 5 |
| DOID:10283 | prostate cancer | 10/45 | 0.000447 | 10 |
| DOID:4450 | renal cell carcinoma | 9/45 | 0.00045 | 9 |
| DOID:3963 | thyroid carcinoma | 7/45 | 0.000509 | 7 |
| DOID:13241 | Behcet's disease | 5/45 | 0.000509 | 5 |
| DOID:4195 | hyperglycemia | 6/45 | 0.000522 | 6 |
| DOID:3856 | male reproductive organ cancer | 10/45 | 0.000523 | 10 |
| DOID:8857 | lupus erythematosus | 5/45 | 0.000525 | 5 |
| DOID:4606 | bile duct cancer | 6/45 | 0.000528 | 6 |
| DOID:4897 | bile duct carcinoma | 6/45 | 0.000528 | 6 |
| DOID:4248 | coronary stenosis | 3/45 | 0.00056 | 3 |
| DOID:1037 | lymphoblastic leukemia | 10/45 | 0.000586 | 10 |
| DOID:1781 | thyroid cancer | 7/45 | 0.000708 | 7 |
| DOID:936 | brain disease | 10/45 | 0.000708 | 10 |
| DOID:3347 | osteosarcoma | 7/45 | 0.000742 | 7 |
| DOID:2218 | blood platelet disease | 5/45 | 0.00079 | 5 |
| DOID:10113 | trypanosomiasis | 3/45 | 0.000861 | 3 |
| DOID:1040 | chronic lymphocytic leukemia | 7/45 | 0.000861 | 7 |
| DOID:0060089 | endocrine organ benign neoplasm | 5/45 | 0.000895 | 5 |
| DOID:1036 | chronic leukemia | 7/45 | 0.000898 | 7 |
| DOID:11162 | respiratory failure | 4/45 | 0.00098 | 4 |
| DOID:438 | autoimmune disease of the nervous system | 4/45 | 0.00098 | 4 |
| DOID:6000 | congestive heart failure | 7/45 | 0.001036 | 7 |
| DOID:3829 | pituitary adenoma | 4/45 | 0.001187 | 4 |
| DOID:1542 | head and neck carcinoma | 7/45 | 0.001187 | 7 |
| DOID:9970 | obesity | 8/45 | 0.001187 | 8 |
| DOID:10964 | cholesteatoma of middle ear | 2/45 | 0.001187 | 2 |
| DOID:11433 | middle ear cholesteatoma | 2/45 | 0.001187 | 2 |
| DOID:14512 | candidal paronychia | 2/45 | 0.001187 | 2 |
| DOID:4045 | muscle cancer | 5/45 | 0.001196 | 5 |
| DOID:11934 | head and neck cancer | 7/45 | 0.001277 | 7 |
| DOID:4007 | bladder carcinoma | 4/45 | 0.001286 | 4 |
| DOID:14069 | cerebral malaria | 3/45 | 0.001325 | 3 |
| DOID:26 | pancreas disease | 6/45 | 0.001335 | 6 |
| DOID:654 | overnutrition | 8/45 | 0.001369 | 8 |
| DOID:184 | bone cancer | 7/45 | 0.001369 | 7 |
| DOID:4607 | biliary tract cancer | 6/45 | 0.001625 | 6 |
| DOID:655 | inherited metabolic disorder | 8/45 | 0.001625 | 8 |
| DOID:2942 | bronchiolitis | 2/45 | 0.001667 | 2 |
| DOID:374 | nutrition disease | 8/45 | 0.001846 | 8 |
| DOID:1588 | thrombocytopenia | 4/45 | 0.00194 | 4 |
| DOID:3908 | non-small cell lung carcinoma | 9/45 | 0.00194 | 9 |
| DOID:12177 | common variable immunodeficiency | 3/45 | 0.00194 | 3 |
| DOID:4798 | aggressive systemic mastocytosis | 3/45 | 0.00194 | 3 |
| DOID:0060085 | organ system benign neoplasm | 7/45 | 0.002042 | 7 |
| DOID:784 | chronic kidney failure | 4/45 | 0.002134 | 4 |
| DOID:0060100 | musculoskeletal system cancer | 9/45 | 0.002174 | 9 |
| DOID:4830 | adenosquamous carcinoma | 2/45 | 0.002206 | 2 |
| DOID:2583 | agammaglobulinemia | 3/45 | 0.002261 | 3 |
| DOID:5100 | middle ear disease | 3/45 | 0.002261 | 3 |
| DOID:620 | blood protein disease | 3/45 | 0.002261 | 3 |
| DOID:9744 | type 1 diabetes mellitus | 3/45 | 0.002261 | 3 |
| DOID:10747 | lymphoid leukemia | 4/45 | 0.002282 | 4 |
| DOID:0060049 | autoimmune disease of urogenital tract | 4/45 | 0.002378 | 4 |
| DOID:12236 | primary biliary cirrhosis | 4/45 | 0.002378 | 4 |
| DOID:2043 | hepatitis B | 6/45 | 0.002492 | 6 |
| DOID:1483 | gingival disease | 3/45 | 0.002634 | 3 |
| DOID:2115 | B cell deficiency | 3/45 | 0.002634 | 3 |
| DOID:120 | female reproductive organ cancer | 9/45 | 0.002634 | 9 |
| DOID:10247 | pleurisy | 2/45 | 0.0027 | 2 |
| DOID:10264 | mumps | 2/45 | 0.0027 | 2 |
| DOID:12155 | lymphocytic choriomeningitis | 2/45 | 0.0027 | 2 |
| DOID:12918 | thromboangiitis obliterans | 2/45 | 0.0027 | 2 |
| DOID:6432 | pulmonary hypertension | 4/45 | 0.002772 | 4 |
| DOID:62 | aortic valve disease | 3/45 | 0.002774 | 3 |
| DOID:201 | connective tissue cancer | 8/45 | 0.002901 | 8 |
| DOID:1116 | pertussis | 3/45 | 0.002991 | 3 |
| DOID:2151 | malignant ovarian surface epithelial-stromal neoplasm | 7/45 | 0.002998 | 7 |
| DOID:2152 | ovary epithelial cancer | 7/45 | 0.002998 | 7 |
| DOID:4001 | ovarian carcinoma | 7/45 | 0.002998 | 7 |
| DOID:5158 | pleural cancer | 3/45 | 0.003168 | 3 |
| DOID:7474 | malignant pleural mesothelioma | 3/45 | 0.003168 | 3 |
| DOID:3234 | central nervous system lymphoma | 2/45 | 0.003286 | 2 |
| DOID:1168 | familial hyperlipidemia | 4/45 | 0.003319 | 4 |
| DOID:3612 | retinitis | 4/45 | 0.003319 | 4 |
| DOID:349 | systemic mastocytosis | 3/45 | 0.003641 | 3 |
| DOID:10155 | intestinal cancer | 7/45 | 0.003827 | 7 |
| DOID:0050598 | extrapulmonary tuberculosis | 2/45 | 0.003916 | 2 |
| DOID:106 | pleural tuberculosis | 2/45 | 0.003916 | 2 |
| DOID:13025 | retinopathy of prematurity | 2/45 | 0.003916 | 2 |
| DOID:4449 | macular retinal edema | 2/45 | 0.003916 | 2 |
| DOID:6929 | retinal edema | 2/45 | 0.003916 | 2 |
| DOID:0050339 | commensal bacterial infectious disease | 3/45 | 0.00409 | 3 |
| DOID:289 | endometriosis | 4/45 | 0.004134 | 4 |
| DOID:5157 | benign pleural mesothelioma | 3/45 | 0.004354 | 3 |
| DOID:2394 | ovarian cancer | 7/45 | 0.004598 | 7 |
| DOID:1319 | brain cancer | 3/45 | 0.004615 | 3 |
| DOID:1586 | rheumatic fever | 2/45 | 0.004615 | 2 |
| DOID:4724 | brain edema | 2/45 | 0.004615 | 2 |
| DOID:3146 | lipid metabolism disorder | 4/45 | 0.004717 | 4 |
| DOID:9408 | acute myocardial infarction | 4/45 | 0.004717 | 4 |
| DOID:3717 | gastric adenocarcinoma | 4/45 | 0.005083 | 4 |
| DOID:350 | mastocytosis | 3/45 | 0.005152 | 3 |
| DOID:3500 | gallbladder adenocarcinoma | 2/45 | 0.005369 | 2 |
| DOID:6196 | reactive arthritis | 2/45 | 0.005369 | 2 |
| DOID:161 | keratosis | 3/45 | 0.00578 | 3 |
| DOID:13608 | biliary atresia | 2/45 | 0.006238 | 2 |
| DOID:6132 | bronchitis | 2/45 | 0.006238 | 2 |
| DOID:10652 | Alzheimer's disease | 8/45 | 0.006476 | 8 |
| DOID:4971 | myelofibrosis | 3/45 | 0.006801 | 3 |
| DOID:680 | tauopathy | 8/45 | 0.006801 | 8 |
| DOID:657 | adenoma | 7/45 | 0.006977 | 7 |
| DOID:2893 | cervix carcinoma | 4/45 | 0.007006 | 4 |
| DOID:2945 | severe acute respiratory syndrome | 2/45 | 0.007064 | 2 |
| DOID:4362 | cervical cancer | 4/45 | 0.007191 | 4 |
| DOID:5520 | head and neck squamous cell carcinoma | 5/45 | 0.007195 | 5 |
| DOID:4948 | gallbladder carcinoma | 3/45 | 0.007411 | 3 |
| DOID:11054 | urinary bladder cancer | 4/45 | 0.007602 | 4 |
| DOID:3121 | gallbladder cancer | 3/45 | 0.007767 | 3 |
| DOID:3969 | papillary thyroid carcinoma | 4/45 | 0.007796 | 4 |
| DOID:11383 | cryptorchidism | 2/45 | 0.007882 | 2 |
| DOID:13378 | Kawasaki disease | 3/45 | 0.008541 | 3 |
| DOID:12603 | acute leukemia | 4/45 | 0.008778 | 4 |
| DOID:11729 | Lyme disease | 2/45 | 0.008865 | 2 |
| DOID:8469 | influenza | 4/45 | 0.008988 | 4 |
| DOID:3962 | follicular thyroid carcinoma | 3/45 | 0.009315 | 3 |
| DOID:2645 | benign mesothelioma | 3/45 | 0.009754 | 3 |
| DOID:1508 | candidiasis | 2/45 | 0.009847 | 2 |
| DOID:1602 | lymphadenitis | 3/45 | 0.010121 | 3 |
| DOID:9942 | lymph node disease | 3/45 | 0.010121 | 3 |
| DOID:0060084 | cell type benign neoplasm | 8/45 | 0.010893 | 8 |
| DOID:219 | colon cancer | 6/45 | 0.01129 | 6 |
| DOID:10286 | prostate carcinoma | 4/45 | 0.011442 | 4 |
| DOID:341 | peripheral vascular disease | 3/45 | 0.011482 | 3 |
| DOID:2957 | pulmonary tuberculosis | 2/45 | 0.011924 | 2 |
| DOID:13580 | cholestasis | 3/45 | 0.011924 | 3 |
| DOID:9256 | colorectal cancer | 6/45 | 0.012055 | 6 |
| DOID:5672 | large intestine cancer | 6/45 | 0.012212 | 6 |
| DOID:715 | T-cell leukemia | 3/45 | 0.012325 | 3 |
| DOID:643 | progressive multifocal leukoencephalopathy | 3/45 | 0.012825 | 3 |
| DOID:12140 | Chagas disease | 2/45 | 0.012825 | 2 |
| DOID:1532 | pleural disease | 2/45 | 0.012825 | 2 |
| DOID:4676 | uremia | 2/45 | 0.012825 | 2 |
| DOID:3070 | malignant glioma | 5/45 | 0.01347 | 5 |
| DOID:750 | peptic ulcer disease | 3/45 | 0.013716 | 3 |
| DOID:3087 | gingivitis | 2/45 | 0.013911 | 2 |
| DOID:9743 | diabetic neuropathy | 2/45 | 0.013911 | 2 |
| DOID:0060180 | colitis | 3/45 | 0.015288 | 3 |
| DOID:8577 | ulcerative colitis | 3/45 | 0.015288 | 3 |
| DOID:1485 | cystic fibrosis | 4/45 | 0.01653 | 4 |
| DOID:2994 | germ cell cancer | 7/45 | 0.016638 | 7 |
| DOID:5683 | hereditary breast ovarian cancer | 5/45 | 0.017481 | 5 |
| DOID:3565 | meningioma | 3/45 | 0.020377 | 3 |
| DOID:0002116 | pterygium | 2/45 | 0.020377 | 2 |
| DOID:10139 | conjunctival degeneration | 2/45 | 0.020377 | 2 |
| DOID:10526 | conjunctival pterygium | 2/45 | 0.020377 | 2 |
| DOID:3192 | neurilemmoma | 2/45 | 0.020377 | 2 |
| DOID:1307 | dementia | 4/45 | 0.021414 | 4 |
| DOID:2513 | basal cell carcinoma | 2/45 | 0.02177 | 2 |
| DOID:633 | myositis | 3/45 | 0.022816 | 3 |
| DOID:13141 | uveitis | 2/45 | 0.023111 | 2 |
| DOID:2001 | neuroma | 2/45 | 0.023111 | 2 |
| DOID:332 | amyotrophic lateral sclerosis | 4/45 | 0.023528 | 4 |
| DOID:1712 | aortic valve stenosis | 2/45 | 0.024559 | 2 |
| DOID:319 | spinal cord disease | 2/45 | 0.027731 | 2 |
| DOID:8029 | sporadic breast cancer | 2/45 | 0.027731 | 2 |
| DOID:0060058 | lymphoma | 3/45 | 0.027854 | 3 |
| DOID:2706 | synovium cancer | 2/45 | 0.029166 | 2 |
| DOID:5485 | synovial sarcoma | 2/45 | 0.029166 | 2 |
| DOID:0060115 | nervous system benign neoplasm | 2/45 | 0.030511 | 2 |
| DOID:2228 | thrombocytosis | 2/45 | 0.030511 | 2 |
| DOID:3277 | thymus cancer | 2/45 | 0.030511 | 2 |
| DOID:3443 | mammary Paget's disease | 2/45 | 0.030511 | 2 |
| DOID:9500 | leukocyte disease | 3/45 | 0.030629 | 3 |
| DOID:10223 | dermatomyositis | 2/45 | 0.031972 | 2 |
| DOID:4251 | conjunctival disease | 2/45 | 0.031972 | 2 |
| DOID:127 | leiomyoma | 3/45 | 0.032917 | 3 |
| DOID:9452 | fatty liver disease | 3/45 | 0.032917 | 3 |
| DOID:12716 | newborn respiratory distress syndrome | 2/45 | 0.033447 | 2 |
| DOID:688 | embryonal cancer | 6/45 | 0.03507 | 6 |
| DOID:0060122 | integumentary system cancer | 3/45 | 0.035156 | 3 |
| DOID:3498 | pancreatic ductal adenocarcinoma | 3/45 | 0.035156 | 3 |
| DOID:4159 | skin cancer | 3/45 | 0.035156 | 3 |
| DOID:2621 | autonomic nervous system neoplasm | 6/45 | 0.035324 | 6 |
| DOID:769 | neuroblastoma | 6/45 | 0.035324 | 6 |
| DOID:255 | hemangioma | 2/45 | 0.036315 | 2 |
| DOID:2742 | auditory system disease | 3/45 | 0.036599 | 3 |
| DOID:75 | lymphatic system disease | 3/45 | 0.037451 | 3 |
| DOID:3713 | ovary adenocarcinoma | 2/45 | 0.037455 | 2 |
| DOID:3973 | thyroid medullary carcinoma | 2/45 | 0.037455 | 2 |
| DOID:783 | end stage renal failure | 2/45 | 0.037455 | 2 |
| DOID:866 | vein disease | 2/45 | 0.037455 | 2 |
| DOID:2473 | opportunistic mycosis | 2/45 | 0.039214 | 2 |
| DOID:231 | motor neuron disease | 4/45 | 0.039692 | 4 |
| DOID:3111 | cystadenocarcinoma | 2/45 | 0.040737 | 2 |
| DOID:3114 | serous cystadenocarcinoma | 2/45 | 0.040737 | 2 |
| DOID:1024 | leprosy | 2/45 | 0.042404 | 2 |
| DOID:8432 | polycythemia | 2/45 | 0.042404 | 2 |
| DOID:1192 | peripheral nervous system neoplasm | 6/45 | 0.042962 | 6 |
| DOID:552 | pneumonia | 3/45 | 0.042962 | 3 |
| DOID:423 | myopathy | 6/45 | 0.04351 | 6 |
| DOID:66 | muscle tissue disease | 6/45 | 0.04351 | 6 |
| DOID:0050136 | systemic mycosis | 2/45 | 0.043674 | 2 |
| DOID:4961 | bone marrow disease | 2/45 | 0.045501 | 2 |
| DOID:1459 | hypothyroidism | 2/45 | 0.047352 | 2 |
| DOID:0080000 | muscular disease | 6/45 | 0.04787 | 6 |
| DOID:0050736 | autosomal dominant disease | 6/45 | 0.048231 | 6 |
| DOID:3113 | papillary carcinoma | 2/45 | 0.048774 | 2 |
| DOID:3458 | breast adenocarcinoma | 2/45 | 0.048774 | 2 |
