# Supplementary material for: Identification of an immune-related risk signature for predicting prognosis in clear cell renal cell carcinoma
Source: Aging (Albany NY). 2020 Feb 6;12(3):2302–32. doi: 10.18632/aging.102746 (PMC7041771; doi:10.18632/aging.102746)
Supplement: Supplementary Table 9 [file aging-12-102746-s005..docx]

Supplementary Table 9. Immune gene sets from ImmPort and InnateDB databases.

| AZGP1 | B2M | CALR | CANX | CD1A |
| --- | --- | --- | --- | --- |
| CD8A | CD8B | CD74 | CREB1 | CTSB |
| PDIA3 | HFE | HLA-A | HLA-B | HLA-C |
| HLA-DPB1 | HLA-DQA1 | HLA-DQA2 | HLA-DQB1 | HLA-DRA |
| HLA-F | HLA-G | HLA-H | MR1 | HSPA1B |
| HSPA8 | HSP90AA1 | HSP90AB1 | ICAM1 | IFNA2 |
| IFNA10 | IFNA13 | IFNA14 | IFNA16 | IFNA17 |
| KIR2DL4 | KIR2DS1 | KIR2DS3 | KIR2DS4 | KIR2DS5 |
| KLRD1 | LTA | CIITA | MICA | MICB |
| PSMC1 | PSMC2 | PSMC3 | PSMC4 | PSMC5 |
| PSMD5 | PSMD7 | PSMD8 | PSMD10 | PSMD11 |
| RFXAP | SLC10A2 | TAP1 | TAP2 | TAPBP |
| PSMD6 | PSME3 | PSMD14 | CLEC4M | IFI30 |
| UBXN1 | ERAP1 | TAPBPL | KIR2DL5A | ERAP2 |
| RAET1L | UBR1 | RAET1G | PDIA2 | CD79A |
| VAV3 | VAV1 | VAV2 | RAC1 | RAC2 |
| PPP3R1 | PPP3R2 | CHP2 | NFAT5 | NFATC1 |
| NRAS | FOS | JUN | CARD11 | BCL10 |
| RELA | NFKBIA | NFKBIB | NFKBIE | CD81 |
| PIK3R3 | PIK3CA | PIK3CB | PIK3CD | PIK3CG |
| CD22 | CD72 | PTPN6 | LILRB3 | FCGR2B |
| IGHA2 | IGHD | IGHE | IGHG1 | IGHG2 |
| IGKC | IGKV1-5 | IGKV2-40 | IGKV3-20 | IGKV3D-11 |
| C5 | CAMP | CCL1 | CCL11 | CCL13 |
| CCL19 | CCL2 | CCL20 | CCL21 | CCL22 |
| CCL28 | CCL3 | CCL3L3 | CCL4 | CCL4L1 |
| CTSG | CX3CL1 | CXCL1 | CXCL10 | CXCL11 |
| CXCL2 | CXCL3 | CXCL5 | CXCL6 | CXCL9 |
| DEFB104A | DEFB4 | EDN1 | EDN2 | EDN3 |
| PF4 | PF4V1 | PLAU | PPBP | PROK2 |
| SEMA3C | SEMA3D | SEMA3E | SEMA3F | SEMA3G |
| CD1B | CD1C | CD1D | CD1E | CD4 |
| CTSE | CTSL1 | CTSS | FCER1G | FCGRT |
| HLA-DMA | HLA-DMB | HLA-DOA | HLA-DOB | HLA-DPA1 |
| HLA-DRB1 | HLA-DRB3 | HLA-DRB4 | HLA-DRB5 | HLA-E |
| HSPA1L | HSPA2 | HSPA4 | HSPA5 | HSPA6 |
| IFNA4 | IFNA5 | IFNA6 | IFNA7 | IFNA8 |
| IFNA21 | IFNG | KIR2DL1 | KIR2DL2 | KIR2DL3 |
| KIR3DL1 | KIR3DL2 | KLRC1 | KLRC2 | KLRC3 |
| NFYA | NFYB | NFYC | LGMN | PSMB8 |
| PSMC6 | PSMD1 | PSMD2 | PSMD3 | PSMD4 |
| PSMD13 | PSME1 | PSME2 | RELB | RFX5 |
| THBS1 | SHFM1 | KLRC4 | AP3B1 | RFXANK |
| PROCR | ADRM1 | KIAA0368 | TRPC4AP | CD209 |
| ULBP3 | ULBP2 | ULBP1 | KIR3DL3 | RAET1E |
| CD79B | LYN | SYK | BTK | BLNK |
| RAC3 | PPP3CA | PPP3CB | PPP3CC | CHP |
| NFATC2 | NFATC3 | NFATC4 | HRAS | KRAS |
| MALT1 | CHUK | IKBKB | IKBKG | NFKB1 |
| CD19 | CR2 | PIK3R5 | PIK3R1 | PIK3R2 |
| AKT3 | AKT1 | AKT2 | GSK3B | INPP5D |
| RASGRP3 | PLCG2 | PRKCB | IFITM1 | IGHA1 |
| IGHG3 | IGHG4 | IGHM | IGHV2-70 | IGHV3-23 |
| IGKV3D-20 | IGKV4-1 | IGLC3 | IGLV7-43 | C3 |
| CCL14 | CCL15 | CCL16 | CCL17 | CCL18 |
| CCL23 | CCL24 | CCL25 | CCL26 | CCL27 |
| CCL5 | CCL7 | CCL8 | CKLF | CMA1 |
| CXCL12 | CXCL13 | CXCL14 | CXCL16 | CXCL17 |
| CYR61 | DEFA1 | DEFA3 | DEFA5 | DEFB1 |
| FGF10 | FGF2 | HTN3 | IL8 | LECT2 |
| RNASE2 | SAA1 | SBDS | SEMA3A | SEMA3B |
| SEMA4A | SEMA4B | SEMA4C | SEMA4D | SEMA4F |
| SEMA4G | SEMA5A | SEMA5B | SEMA6A | SEMA6B |
| TNC | TYMP | XCL1 | XCL2 | C5AR1 |
| CCR5 | CCR6 | CCR7 | CCR8 | CCR9 |
| CXCR4 | CXCR5 | CXCR6 | CXCR7 | CYSLTR1 |
| FPR2 | GPR17 | GPR32 | GPR33 | GPR44 |
| PLAUR | PLXNA1 | PLXNA2 | PLXNA3 | PLXNA4 |
| PTAFR | ROBO1 | ROBO2 | ROBO3 | RXFP3 |
| AGT | AMBN | AMELX | AMH | ANGPTL5 |
| ARTN | AVP | AZU1 | BDNF | BMP1 |
| BMP5 | BMP6 | BMP7 | BMP8A | BMP8B |
| CCK | CD320 | CD40LG | CD70 | CECR1 |
| CHGB | CLCF1 | CLEC11A | CMTM1 | CMTM2 |
| CMTM8 | CNTF | CORT | CRH | CSF1 |
| CTF1 | CTGF | DKK1 | EBI3 | EGF |
| FAM3C | FAM3D | FASLG | FGF1 | FGF11 |
| FGF18 | FGF19 | FGF20 | FGF21 | FGF22 |
| FGF7 | FGF8 | FGF9 | FIGF | FIGNL2 |
| GCG | GDF1 | GDF10 | GDF11 | GDF15 |
| GDF9 | GDNF | GH1 | GH2 | GHRH |
| GNRH1 | GNRH2 | GPHA2 | GPHB5 | GPI |
| HAMP | HBEGF | HDGF | HDGFRP3 | HGF |
| IGF1 | IGF2 | IL10 | IL11 | IL12A |
| IL17B | IL17C | IL17D | IL17F | IL18 |
| IL1F6 | IL1F7 | IL1F8 | IL1F9 | IL1RN |
| IL24 | IL25 | IL26 | IL27 | IL28A |
| IL33 | IL34 | IL4 | IL5 | IL6 |
| INHBB | INHBC | INHBE | INS | INSL3 |
| KGFLP1 | KGFLP2 | KITLG | KL | LACRT |
| LRSAM1 | LTB | LTBP1 | LTBP2 | LTBP3 |
| MSTN | NAMPT | NDP | NENF | NGF |
| NPPB | NPPC | NPY | NRG1 | NRG2 |
| NTS | NUDT6 | OGN | OSGIN1 | OSM |
| SEMA6C | SEMA6D | SEMA7A | SLIT1 | SLIT2 |
| CCBP2 | CCR1 | CCR10 | CCR3 | CCR4 |
| CCRL1 | CCRL2 | CMKLR1 | CX3CR1 | CXCR3 |
| CYSLTR2 | DARC | EDNRA | EDNRB | FPR1 |
| GPR77 | IL8RA | IL8RB | LTB4R | LTB4R2 |
| PLXNB1 | PLXNB2 | PLXNB3 | PLXNC1 | PLXND1 |
| XCR1 | ADIPOQ | ADM | ADM2 | AGRP |
| ANGPTL7 | APLN | AREG | ARMET | ARMETL1 |
| BMP10 | BMP15 | BMP2 | BMP3 | BMP4 |
| BTC | C19orf10 | CALCA | CALCB | CAT |
| CER1 | CGA | CGB | CGB2 | CHGA |
| CMTM3 | CMTM4 | CMTM5 | CMTM6 | CMTM7 |
| CSF2 | CSF3 | CSH1 | CSHL1 | CSPG5 |
| EPGN | EPO | EREG | ESM1 | FAM3B |
| FGF12 | FGF13 | FGF14 | FGF16 | FGF17 |
| FGF23 | FGF3 | FGF4 | FGF5 | FGF6 |
| FLT3LG | FSHB | GAL | GALP | GAST |
| GDF2 | GDF3 | GDF5 | GDF6 | GDF7 |
| GHRL | GIP | GKN1 | GMFB | GMFG |
| GREM1 | GREM2 | GRN | GRP | GUCA2A |
| IAPP | IFNB1 | IFNE | IFNK | IFNW1 |
| IL12B | IL13 | IL15 | IL16 | IL17A |
| IL19 | IL1A | IL1B | IL1F10 | IL1F5 |
| IL2 | IL20 | IL21 | IL22 | IL23A |
| IL28B | IL29 | IL3 | IL31 | IL32 |
| IL6ST | IL7 | IL9 | INHA | INHBA |
| INSL4 | INSL5 | INSL6 | JAG1 | JAG2 |
| LEFTY1 | LEFTY2 | LEP | LHB | LIF |
| LTBP4 | MDK | MIA | MIF | MLN |
| NMB | NODAL | NOV | NPFF | NPPA |
| NRG3 | NRG4 | NRTN | NTF3 | NTF4 |
| OSTN | OXT | P11 | PDGFA | PDGFB |
| PDGFC | PDGFD | PDGFRA | PDGFRB | PDGFRL |
| PSPN | PTH | PTH2 | PTHLH | PTN |
| S100A6 | SCG2 | SCGB3A1 | SCT | SCYE1 |
| TGFA | TGFB1 | TGFB2 | TGFB3 | THPO |
| TNFSF4 | TNFSF8 | TNFSF9 | TOR2A | TRH |
| VEGFC | VGF | VIP | ACVR1B | ACVR1C |
| AMHR2 | ANGPT1 | ANGPT4 | ANGPTL1 | ANGPTL2 |
| BMPR2 | BRD8 | C3AR1 | CALCR | CALCRL |
| CSF2RB | CSF3R | EGFR | ENG | EPOR |
| FLT1 | FLT3 | FLT4 | FSHR | GALR2 |
| HNF4A | HNF4G | HTR3A | HTR3B | HTR3C |
| IL11RA | IL12RB1 | IL12RB2 | IL13RA1 | IL13RA2 |
| IL1RAP | IL1RL1 | IL1RL2 | IL20RA | IL20RB |
| IL3RA | IL4R | IL5RA | IL6R | IL7R |
| MC1R | MC2R | MC3R | MC4R | MCHR1 |
| NR0B1 | NR0B2 | NR1D1 | NR1D2 | NR1H2 |
| NR2F6 | NR3C1 | NR3C2 | NR4A1 | NR4A2 |
| OPRM1 | OSMR | OXTR | PGR | PGRMC2 |
| PTGER4 | PTGFR | PTH1R | PTH2R | RARA |
| S1PR1 | S1PR2 | SCTR | SDC1 | SDC2 |
| TGFBR3 | THRA | THRB | TIE1 | TNFRSF10A |
| TNFRSF19 | TNFRSF1A | TNFRSF1B | TNFRSF21 | TNFRSF25 |
| PTPN11 | ICAM2 | ITGAL | ITGB2 | PTK2B |
| NCR1 | NCR3 | CD247 | ZAP70 | LCP2 |
| SOS2 | ARAF | BRAF | RAF1 | KLRK1 |
| CASP3 | BID | CD3D | CD3E | CD3G |
| PAK7 | RHOA | CDC42 | CD28 | ICOS |
| PRKCQ | TRBC1 | TRBV12-3 | TRGV3 | COLEC12 |
| ELP2 | SLC2A11 | PIK3C3 | RCAN1 | SIGLEC15 |
| MX1 | PMAIP1 | PRKD1 | ABCG1 | PHLPP1 |
| PDYN | PENK | PGF | PMCH | PNOC |
| PYY | QRFP | RABEP1 | RABEP2 | REG1A |
| SECTM1 | SLURP1 | SPP1 | SST | STC1 |
| TNF | TNFRSF11B | TNFSF10 | TNFSF11 | TNFSF12 |
| TSHB | TSLP | TXLNA | UCN | UCN2 |
| ACVR2A | ACVR2B | ACVRL1 | ADCYAP1R1 | ADIPOR1 |
| ANGPTL3 | ANGPTL4 | ANGPTL6 | APLNR | AR |
| CD40 | CNTFR | CRHR1 | CRHR2 | CRIM1 |
| ESR1 | ESR2 | ESRRA | ESRRB | ESRRG |
| GALR3 | GCGR | GHR | GHRHR | GHSR |
| HTR3D | HTR3E | IFNAR1 | IFNAR2 | IFNGR1 |
| IL15RA | IL17RA | IL17RB | IL17RC | IL17RD |
| IL21R | IL22RA1 | IL22RA2 | IL23R | IL27RA |
| IL9R | INSR | KDR | LEPR | LGR4 |
| MCHR2 | MET | MLNR | MPL | MTNR1A |
| NR1H3 | NR1H4 | NR1I2 | NR1I3 | NR2C1 |
| NR4A3 | NR5A1 | NR5A2 | NR6A1 | NRP1 |
| PPARA | PPARD | PPARG | PRLHR | PRLR |
| RARB | RARG | RORA | RORB | RORC |
| SDC3 | SDC4 | SORT1 | SSTR1 | SSTR2 |
| TNFRSF10B | TNFRSF10C | TNFRSF10D | TNFRSF11A | TNFRSF12A |
| TNFRSF4 | TNFRSF6B | TNFRSF8 | TNFRSF9 | TRHR |
| PAK1 | MAP2K1 | MAP2K2 | MAPK1 | MAPK3 |
| LAT | PLCG1 | SH3BP2 | FYN | SHC2 |
| HCST | CD48 | CD244 | PRKCA | PRKCG |
| PTPRC | ITK | TEC | NCK1 | NCK2 |
| MAP3K8 | MAP3K14 | PDCD1 | CTLA4 | CBLC |
| YES1 | NRIP1 | APP | PTPN2 | GATA6 |
| PIAS2 | CEBPE | SMAD7 | XBP1 | SMAD4 |
| BCL2 | SOCS6 | FOXA1 | TTC6 | AIRE |
| POMC | PPY | PRL | PRLH | PROK1 |
| RETN | RETNLB | RLN1 | RLN2 | RLN3 |
| STC2 | TAC1 | TDGF1 | TDGF3 | TG |
| TNFSF13 | TNFSF13B | TNFSF14 | TNFSF15 | TNFSF18 |
| UCN3 | UTS2 | UTS2D | VEGFA | VEGFB |
| ADIPOR2 | ADRB1 | ADRB2 | AGTR1 | AGTR2 |
| AVPR1A | AVPR1B | AVPR2 | BMPR1A | BMPR1B |
| CRLF1 | CRLF2 | CRLF3 | CSF1R | CSF2RA |
| FGFR1 | FGFR2 | FGFR3 | FGFR4 | FGFRL1 |
| GIPR | GLP1R | GLP2R | GNRHR | GPER |
| IFNGR2 | IGF1R | IGF2R | IL10RA | IL10RB |
| IL17RE | IL18R1 | IL18RAP | IL1R1 | IL1R2 |
| IL28RA | IL2RA | IL2RB | IL2RG | IL31RA |
| LGR5 | LGR6 | LHCGR | LIFR | LTBR |
| MTNR1B | NGFR | NMBR | NPR1 | NPR3 |
| NR2C2 | NR2E1 | NR2E3 | NR2F1 | NR2F2 |
| NRP2 | OGFR | OPRD1 | OPRK1 | OPRL1 |
| PTGDR | PTGDS | PTGER1 | PTGER2 | PTGER3 |
| RXFP1 | RXFP2 | RXRA | RXRB | RXRG |
| SSTR5 | TACR1 | TEK | TGFBR1 | TGFBR2 |
| TNFRSF13B | TNFRSF13C | TNFRSF14 | TNFRSF17 | TNFRSF18 |
| TSHR | TUBB3 | VDR | VIPR1 | VIPR2 |
| NCR2 | TYROBP | LCK | FCGR3A | FCGR3B |
| SHC4 | SHC3 | SHC1 | GRB2 | SOS1 |
| SH2D1B | SH2D1A | FAS | GZMB | PRF1 |
| GRAP2 | PAK2 | PAK3 | PAK4 | PAK6 |
| CBL | CBLB | CDK4 | RASGRP1 | PDK1 |
| HRH4 | CRKL | RNF125 | RNASE3 | RNASE7 |
| RNF31 | TCF4 | ADCY4 | RIPK3 | MX2 |
| TRPM2 | HMOX1 | ADARB1 | APOL1 | MYH9 |
| ARF6 | HIF1A | CREBBP | PPIA | PIAS4 |
| DEFA6 | TPSB2 | GZMM | UCHL1 | SMAD6 |
| DEFA4 | TNRC6B | ADCY9 | DUSP4 | CLEC1B |
| PRKAR1B | AGR2 | ELMO1 | ADCY1 | SMAD3 |
| DEFB4B | EP300 | PRTN3 | C1S | CLEC9A |
| DEFB103B | AHR | FAM105B | CALM1 | CLEC7A |
| SPON2 | ZBTB1 | ELANE | C1R | CARD6 |
| TRIM9 | POLR3H | MAPK11 | C1RL | POLR2L |
| S100B | XRCC6 | CFD | NLRP6 | C7 |
| DEFB103A | SEPX1 | MYO10 | RPS6KA5 | DICER1 |
| LGALS2 | SREBF2 | TLR10 | IFITM2 | CHID1 |
| DEFB4A | LZTS1 | TLR1 | IFITM3 | C6 |
| POLR3K | TSC2 | TLR6 | SKP2 | TPST1 |
| LGALS1 | DUOX2 | TRIM35 | CLEC4C | IDO1 |
| POLR2F | MLST8 | POLR2E | SIGIRR | STAP2 |
| TYRO3 | DUOX1 | USP7 | CLEC4A | PIAS1 |
| LGALS3 | HOXA9 | CD27 | CLEC6A | KIT |
| PLA2G6 | PDPK1 | ADAM10 | CLEC4D | BDKRB2 |
| GATA4 | TAX1BP1 | KLB | CLEC4E | TOLLIP |
| PELI2 | POLR3D | ITPR1 | IRF7 | ARRB2 |
| ACTB | WIPF3 | CLU | AICDA | POLR1D |
| APOBEC3A | TCEB2 | NXN | KLRG1 | TICAM1 |
| APOBEC3B | NOD1 | SOCS1 | CACTIN | CTSD |
| SNAP23 | KCNIP4 | YWHAE | RICTOR | HDAC11 |
| APOBEC3F | PDE1C | CRK | RAB11A | PLD2 |
| STUB1 | MEFV | ANXA2 | OTUB2 | TRIM50 |
| APOBEC3H | PPARGC1A | ING4 | C9 | POLR3E |
| TAB1 | ADCY2 | MYO1C | IRAK2 | YY1 |
| MSR1 | NLRC3 | C1QTNF3 | IFI27 | DUSP16 |
| ATF4 | SLX4 | SCARF1 | GP2 | CDKN1B |
| DLK1 | PPP4C | CCNT1 | USP4 | DUSP7 |
| REST | ACAA1 | ADCY6 | MAPK7 | DCD |
| PLK1 | BCL2A1 | TRIB2 | PGLYRP2 | CD59 |
| LIMK1 | MYD88 | NLRP10 | SRPK2 | ELMOD2 |
| LAT2 | UBE2W | MYH2 | AKAP10 | WASL |
| HMGB1 | TCEB1 | ADCY7 | RGMB | CD63 |
| UBE2V2 | CORO1A | POLR2K | PRKAR2B | CAPRIN1 |
| RCOR1 | LY96 | NOD2 | MRGPRX2 | GAB1 |
| TNRC6A | FZD1 | CYLD | IP6K1 | RNF135 |
| TRAF3 | SCN5A | TRIM66 | MYC | PLEC |
| RFTN1 | PIN1 | DDX1 | UBA7 | ERBB3 |
| KHSRP | DNM1L | EIF4E | MAP2K3 | NDUFA13 |
| PML | TP53 | MAP2K4 | TRAIP | SHARPIN |
| C1QBP | FOXO1 | IPO7 | SUGT1 | PRKCD |
| DHX33 | RASGEF1B | LPCAT2 | MST1R | TRAF6 |
| EPS8 | CDK6 | SWAP70 | ADCY8 | RAG1 |
| NCF1 | ELF1 | RAD23A | LGALS9 | RNF41 |
| KAT2B | RSAD2 | MEF2A | F2RL3 | SIRPB1 |
| NLRP1 | TYK2 | AMFR | NOS2 | IRF5 |
| MAP3K1 | LRRK2 | GNB2 | TRADD | HSF1 |
| NKIRAS1 | TRIM6 | RB1 | TNIP3 | SELK |
| CSK | CTNNB1 | UBE2D3 | GNAI2 | SIRPA |
| IGJ | TRIM5 | ZFPM2 | OLFM4 | STAT2 |
| SIVA1 | TRIM22 | UBB | CCNA2 | PHLPP2 |
| SCAMP5 | RIPK2 | TRPV2 | SLC25A46 | PDE12 |
| KCNJ8 | ILF3 | ACHE | SEBOX | PRKX |
| CLEC10A | PYCARD | EDIL3 | SARM1 | TLR2 |
| AZI2 | IRAK4 | TBC1D27 | BST2 | STAT6 |
| ANKRD17 | PYDC1 | ATRIP | DEFB127 | IRF1 |
| MTA1 | ITGAM | NLRC5 | CAMK4 | BCAR1 |
| TUFM | ITGAX | ATF1 | PCBP2 | GLI1 |
| TRIM55 | ANO6 | PALM3 | IFRD1 | CNOT4 |
| TRIM71 | MAPK10 | PRKACA | ADAD1 | XDH |
| MAP2K7 | SCAF11 | TRIM56 | MAP3K12 | DDIT3 |
| CD36 | TECPR1 | SERPINE1 | CTCF | MID1 |
| TRIM23 | SMARCA4 | MEF2C | EIF2C2 | TRIM24 |
| PSTPIP1 | ISG20 | CD97 | PTK2 | SPI1 |
| ELAVL1 | TPT1 | POLR3G | TRIB3 | NLRC4 |
| ITPR2 | PRKCSH | SREBF1 | CISH | TRIM61 |
| COPS5 | ECSIT | AIMP1 | JAK3 | TRIM60 |
| ART1 | F2RL2 | PKN1 | MAPKAPK3 | ZC3HAV1 |
| ERBB2IP | NTN1 | RAD21 | RBCK1 | ZMYND11 |
| ACAP1 | F2R | PRKAR2A | CAV1 | DDX60 |
| STIM1 | F2RL1 | CASP6 | CAPZA2 | SMARCA2 |
| LRRFIP2 | E2F6 | CFI | TRIM36 | TLR7 |
| TNK1 | HERC5 | GLRX | CFTR | FFAR2 |
| TRIM21 | NLRP14 | ADCY3 | TMED7 | TLR8 |
| CD180 | SNCA | IMPDH2 | ATG12 | HMGB2 |
| POSTN | SIAH1 | TRIM13 | NCKAP1L | VLDLR |
| CTSH | CCR2 | TIFA | PDE1B | EIF2AK2 |
| TBK1 | POLR3B | BPIFA1 | NCKAP1 | TSC1 |
| IRF8 | ANXA4 | MAP2K6 | YWHAB | IRAK1 |
| RPL19 | CNOT8 | IL37 | FANCC | CASP9 |
| IRAK3 | PCBP1 | FXR1 | ITGAV | FBXO9 |
| PROS1 | MAP4K2 | TRIM38 | PTCH1 | GRK5 |
| ERBB2 | HAVCR2 | E2F1 | WFDC12 | PPAPDC1A |
| ZFPM1 | CYFIP2 | CD300A | STAT1 | PADI4 |
| WDR62 | IRF4 | CD300LB | STAT4 | FCN2 |
| IRF2 | WNT9B | NOX4 | HSPD1 | FCN1 |
| CYBA | CYBB | CD300E | LST1 | DMBT1 |
| BMX | DDX58 | ACTR3 | PLCL1 | C6orf57 |
| MDM2 | HSPA14 | CD300LF | TRIM14 | GPSM1 |
| ACE2 | TBKBP1 | TRIM49 | CORO2A | CARD9 |
| GSDMB | PIK3R4 | NLRP12 | CFLAR | MB21D1 |
| ORMDL3 | BCL3 | MARCO | CASP10 | NOTCH1 |
| GSDMA | SERPINB9 | ITCH | CASP8 | PLA2G2A |
| UBE2L6 | ARPC3 | PANX1 | PLTP | TRAF2 |
| SERPING1 | POLR3F | POLR2H | MMP9 | FBXW5 |
| JAK2 | CALCOCO2 | MRE11A | ELMO2 | C8G |
| FRS2 | PPP1CC | ITGB1 | HSPA1A | MUL1 |
| EGR1 | RIPK1 | LILRA5 | NOX1 | DOCK1 |
| TLR3 | VASP | LILRA4 | NEU1 | IRAK1BP1 |
| WIPF2 | DUSP1 | SENP2 | ABCA1 | NOXA1 |
| TOMM70A | CFL1 | LAIR1 | EHMT2 | VENTX |
| ADAM33 | TRAFD1 | AHSG | CFB | C1QA |
| CD274 | AQP3 | PARD3 | POLR3A | C1QC |
| PDCD1LG2 | ITGA3 | HRG | ERBB4 | C1QB |
| F11 | OAS1 | BIRC3 | CTNNAL1 | KDM1A |
| SIGLEC1 | IRF2BP1 | TRIM47 | SFTPA2 | AKIRIN2 |
| SMARCE1 | OAS3 | BIRC2 | SFTPA1 | RUNX3 |
| RARRES2 | OAS2 | TRIM65 | SFTPD | MAP3K7 |
| TMEM173 | PGLYRP1 | MMP7 | TXN | TRIM63 |
| UBE2D2 | PRSS3 | BTN3A2 | RNF5 | ASCC3 |
| DTX4 | CALM3 | MMP8 | AGER | HMGN2 |
| NFKBIZ | TRIM42 | PRKACG | XRCC5 | RPS6KA1 |
| PURA | PELI3 | MASP1 | CXCR2 | HACE1 |
| TPP2 | FOXA2 | BTN3A1 | CXCR1 | ATG5 |
| MS4A2 | LY86 | BTN3A3 | ARPC2 | FCN3 |
| MAVS | ADRBK1 | LPP | CEBPB | WASF2 |
| TRAT1 | F12 | TP63 | AAMP | FGR |
| CD14 | PLSCR1 | CASP12 | ATG9A | IFI6 |
| MS4A8B | PPP1CA | CASP4 | PTEN | FOXO3 |
| LGALS4 | TRIM25 | CASP1 | TSC22D3 | HDAC2 |
| HNRNPL | RPS6KB2 | SPHK1 | MID2 | HDAC1 |
| SIRT2 | DOK3 | CARD16 | IFIT2 | GOPC |
| CD200 | DDX41 | RHBDF2 | IFIT3 | TRIM62 |
| DAK | AIP | NLRP7 | IFIT1 | GJA1 |
| PRKCE | GSTP1 | NLRP2 | IFIT5 | EIF2C4 |
| NKIRAS2 | UNC93B1 | TNRC6C | AKNA | EIF2C1 |
| IRS2 | P2RX7 | HSPBP1 | ZBP1 | EIF2C3 |
| DHX58 | SIAH2 | BIRC5 | IRS1 | ZC3H12A |
| INSIG1 | CAMKK2 | SOCS3 | 5-Mar | ARG1 |
| DUSP6 | P2RY14 | SSC5D | MFF | VNN1 |
| STAT5B | TRIM37 | ANXA1 | AGFG1 | SMAP2 |
| STAT5A | CLTC | NLRP9 | TRIM32 | MAP3K5 |
| SOCS5 | CFP | NLRP11 | TLR4 | TNFAIP3 |
| STAT3 | SQSTM1 | NLRP4 | ITPR3 | TAB2 |
| CALM2 | ELK1 | NLRP13 | PTMA | MKNK1 |
| RPS6KA3 | DHX36 | NLRP8 | TRAF1 | SLC22A3 |
| FREM1 | FADD | LRRC33 | ATG16L1 | PLG |
| DCN | MAPK9 | NLRP5 | PACSIN1 | MAP3K4 |
| UBE2N | COX5B | ARHGAP15 | DAB2IP | PARK2 |
| CD80 | CD37 | KIAA0226 | PSMA7 | RPS6KA2 |
| SOCS2 | RANBP9 | BAIAP2 | PIK3AP1 | DHCR24 |
| BECN1 | DEFB118 | ZBTB16 | COPS8 | C8A |
| FSTL1 | TRIM7 | TRIM27 | ISG15 | C8B |
| NUMBL | PTX3 | ACTG1 | LRRFIP1 | JAK1 |
| SCGB3A2 | GNB2L1 | APOA1 | SRPK1 | PRKACB |
| CD86 | IRF3 | CYTIP | MAPK14 | GBP2 |
| SPINK5 | PRMT1 | TRIM28 | CDKN1A | DR1 |
| RPS27A | NUP153 | MAPK8 | XIAP | GNAI3 |
| CCDC88A | MAP3K3 | TANK | MAPKAP1 | GSTM1 |
| KPNA1 | BCL2L1 | UBQLN1 | ELF4 | CD53 |
| GATA3 | AKT1S1 | SAMHD1 | CDK9 | WNT2B |
| AXL | WAS | IFIH1 | UNC5CL | CAPZA1 |
| DUSP3 | SIGLEC11 | SRC | TREML1 | MOV10 |
| ADCY5 | SCARB1 | NLRX1 | TREM2 | SIKE1 |
| OTUB1 | ABI1 | USP2 | TREML2 | TRIM45 |
| PPARGC1B | UBC | BPI | HIF1AN | POLR3GL |
| REL | UCP2 | MBL2 | TREM1 | PIAS3 |
| RPS19 | PQBP1 | LBP | TP73 | POLR3C |
| BAD | SLC15A4 | FOXO4 | PTGES2 | FCGR1A |
| CAMK2A | HCK | UBE2D1 | CNPY3 | VPS45 |
| IL1RAPL1 | ERN1 | MAFB | LCN2 | CTSK |
| GAS6 | OTUD5 | CDK1 | PPP2R5D | TNFAIP8L2 |
| PELI1 | KLK1 | WIPF1 | BRS3 | SNX27 |
| RPS6KA4 | ULK1 | ATF2 | NFKB2 | THEM4 |
| ITGB5 | APOH | TRIM31 | TRIM8 | S100A10 |
| GSK3A | SIGLEC9 | NFIL3 | POLR1C | PGLYRP3 |
| TAB3 | SIGLEC7 | TRIM10 | WDR34 | PGLYRP4 |
| HSP90B1 | SYP | TRIM15 | HMGB3 | S100A9 |
| TNIP1 | SIGLEC10 | TIRAP | MASP2 | S100A12 |
| ACTR2 | PRKAR1A | SIRT1 | PTGES | S100A8 |
| IFNA1 | BPIFB3 | NFE2L2 | MTOR | S100A7 |
| CEACAM1 | SIGLEC8 | ETS1 | BGN | ADAR |
| EFTUD2 | GAB2 | FLI1 | ABL1 | MUC1 |
| OPTN | MERTK | PRKRA | NUP214 | PKLR |
| CDKN2A | SIGLEC6 | DDX21 | MFN2 | RUSC1 |
| CEACAM8 | MFN1 | JAM3 | CASP7 | ARHGEF2 |
| G3BP1 | SIGLEC5 | PDE1A | CRISP3 | CD5L |
| IFI16 | CFH | C2 | MIR23A | TREX1 |
| AIM2 | MAPKAPK2 | C4A | MIR378A | NCKIPSD |
| FCER1A | C4BPB | MIR148B | MIR499A | MIR208B |
| APCS | C4BPA | MIR122 | MIR16-1 | MIR1275 |
| CRP | CD55 | MIR107 | MIR372 | CEBPD |
| SLAMF8 | CR1 | MIR125B2 | MIR23B | UBA52 |
| SLAMF9 | CD46 | MIR373 | MIR485 | MIR548G |
| SLAMF6 | IRF6 | MIRLET7B | MIR200C | DEFT1P2 |
| SLAMF1 | TRAF5 | MIRLET7E | MIR125B1 | USP17L11 |
| SLAMF7 | ATF3 | MIR141 | MIR517A | TRIM26 |
| LY9 | DUSP10 | MIR130A | MIR223 | C4B |
| FCGR2A | TLR5 | MIRLET7C | MIR517C | IRGM |
| SELE | WNT3A | MIR16-2 | MIR10B | NAIP |
| RNASEL | TRIM11 | MIR203 | MIR15B | DEFA1B |
| DHX9 | TRIM67 | MIR21 | MIR187 | APOBEC3D |
| NCF2 | LGALS8 | MIR328 | MIR146B | APOBEC3C |
| ARPC5 | MAP1LC3C | MIR181A2 | MIR152 | APOBEC3G |
| PTGS2 | NLRP3 | MIR362 | DDX3X | IGKV1-8 |
| PLA2G4A | TRIM58 | MIR148A | IRF9 | IGKV3-11 |
| RGS2 | IGKV5-2 | MIR126 | UBD | TICAM2 |
| ARPC4 | UBE2V1 | LILRA2 | IGLL5 | MIR29A |
| TLR9 | CEBPA | SIGLEC14 | ITGB3 | MIR155 |
| CRCP | DDOST | AC005013 | MIR3148 | MIR146A |
| PRKDC | ARPC1A | BRK1 | EGLN2 | PRSS3P2 |
| IKBKE | TXNIP | CLEC5A |  |  |
